# eHistology image and annotation data from the Kaufman Atlas of Mouse Development

**DOI:** 10.1093/gigascience/gix131

**Published:** 2017-12-20

**Authors:** Richard A Baldock, Chris Armit

**Affiliations:** MRC Human Genetics Unit, Institute of Genomic and Molecular Medicine, University of Edinburgh, Crewe Road, Edinburgh EH4 2XU, UK

**Keywords:** embryo, mouse, section, atlas, anatomy, imaging

## Abstract

“The Atlas of Mouse Development” by Kaufman is a classic paper atlas that is the *de facto* standard for the definition of mouse embryo anatomy in the context of standard histological images. We have redigitized the original haematoxylin and eosin–stained tissue sections used for the book at high resolution and transferred the hand-drawn annotations to digital form. We have augmented the annotations with standard ontological assignments (EMAPA anatomy) and made the data freely available via an online viewer (eHistology) and from the University of Edinburgh DataShare archive. The dataset captures and preserves the definitive anatomical knowledge of the original atlas, provides a core image set for deeper community annotation and teaching, and delivers a unique high-quality set of high-resolution histological images through mammalian development for manual and automated analysis.

## Data Description

“The Atlas of Mouse Development” [1] is a book detailing the anatomy of mouse embryo development and stands as the definitive work in the field. The atlas is based on a lifetime of work by Kaufman, who established a unique set of histological sections of about 450 mouse embryos, many of which are full serial section-series, from which he selected carefully staged samples for the histological images within the book. The combination of the histological section series and the printed book represent a unique resource and captures the current understanding of classical mouse anatomy. In taxonomic terms, these physical sections are the *reference specimens* for the definition of mouse embryo anatomy, and the digitized images with the associated annotations are a digital *holotype* for the definition of anatomical terms and the progression of mouse embryo development. In addition, the paper atlas has given rise to the Mouse Atlas programme in Edinburgh [2] and to the EMAPA mouse anatomy ontology [3,4]. The original index for the book was used to develop the primary list of anatomical terms in the ontology, and EMAPA is now recognized as the standard mouse embryo ontology used to annotate mouse embryo data including embryo phenotype data [5,6].

**Figure 1:**
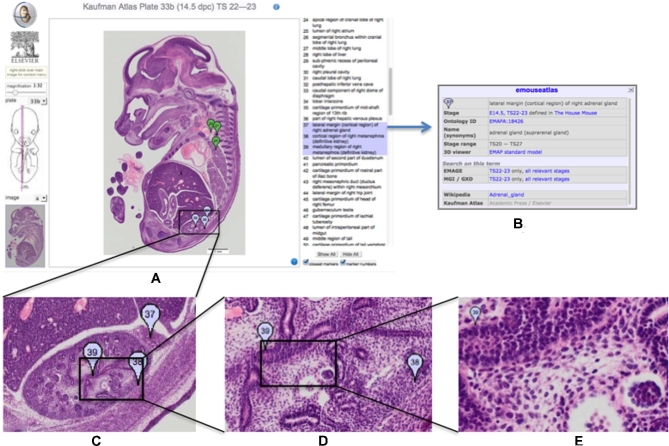
Kaufman Atlas eHistology Viewer. A, Screen shot showing the user interface with annotation list. The 3 selected terms showing as a blue “flags” numbers 37, 38, 39 are over the developing kidney. B, The pop-up dialog with extra information on selecting the “37.” C–E, Progressively higher-resolution images corresponding to zooming-in on the image. At full resolution, the pixel spacing is 0.34 × 0.34 microns and reveals cellular architecture and arrangements.

In generating the eHistology Atlas, new images of the histological sections were acquired at high resolution, and the annotations have been transferred to a database. These images and annotations are now freely available from the eMouseAtlas web resource as eHistology (Fig. 1) and have been described by Graham et al. [7]. The new high-resolution images and the associated image coordinates for each annotation are fully freely available under a Creative Commons CC BY 4.0 licence. In addition, we have an agreement with Elsevier to present the images in a form similar to the original atlas plate layout (the web resource), and Elsevier is able to use the new images for their own version of an online version if they want to. Here we describe the dataset of the 937 high-resolution histology images with anatomy annotations and how they have been made available for further study and analysis.

**Figure 2:**
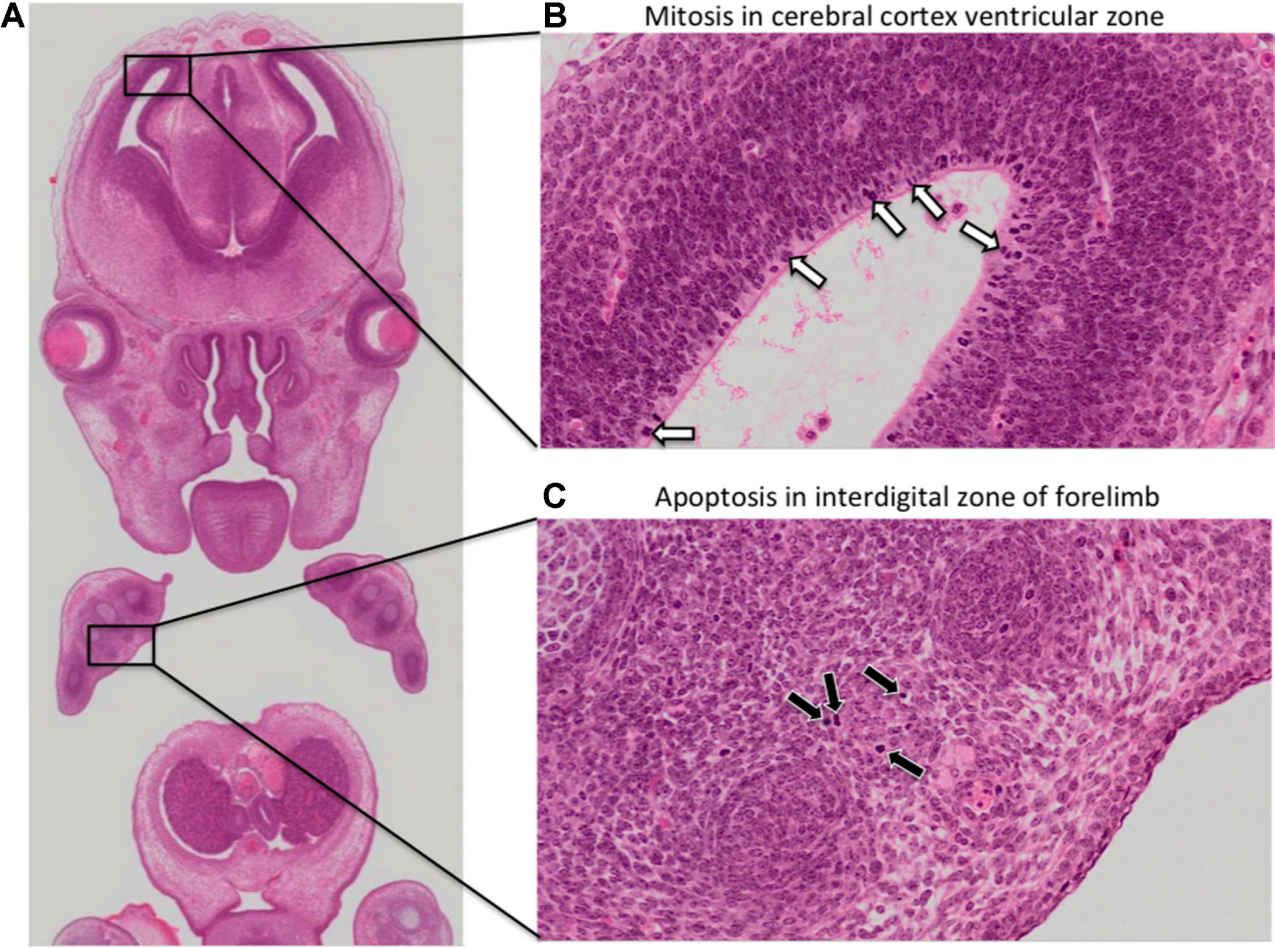
Observing mitosis and apoptosis in cellular resolution eHistology atlas images. A key advantage of capturing histology images at high resolution is the ability to morphologically identify mitotically dividing cells and apoptotic cells in embryo atlas images. A, Zoomed-out view of a coronal image of an E14.5 embryo. B, On the zoomed-in view, neuroblasts in the ventricular zone of the cerebral cortex show intense haematoxylin staining (white arrows), a morphological feature associated with chromosome condensation in mitotically dividing cells. C, On the zoomed-in view, scattered cells in the interdigital zone of the forelimb are pyknotic (black arrows), a morphological feature associated with nuclear condensation. The pyknotic cells additionally show signs of cell shrinkage. These are morphological hallmarks of apoptosis.

The motivation for the eHistology resource was to capture the anatomical knowledge in a permanently accessible open and digital form delivered with a viewer providing a view of the underlying histology data not possible in the printed atlas. The high-resolution images provide a rich resource of carefully staged mouse histology, which could be used for deeper analysis of tissue development and as a teaching resource. See Fig. 1 for an illustration of the resolution now available for these images. Embryogenesis is a highly dynamic process, and in Fig. 2 we highlight some of the advantages of capturing images at cellular resolution, e.g., the ability to zoom in and morphologically identify mitotically dividing cells and apoptotic cells undergoing programmed cell death. This is simply not possible in the print version of the atlas and represents a significant contribution to the community.

All the data are available under a creative commons licence (CC BY 4.0). In the future, we envisage the annotations being extended on a tissue-by-tissue basis through community curation. The eHistology viewer is open source and is available from the Mouse Atlas technical GitHub repository (github.com/ma-tech) (eHistology; RRID:SCR_015887).

Providing secure and long-term accessibility for research data is a difficult problem. A recent study of the longevity of 375 biomedical resources/databases [8] available on the web in 1997 found that 62.3% had ceased to be available, 14.4% were static, and only 23.3% were available as an active resource. The authors concluded that survival depended primarily on institutional interest and that a strategy dependent on external funding will very likely fail. To ensure long-term preservation of the image data and supporting annotations, we have therefore registered this dataset with the University of Edinburgh *DataShare* [9] repository [10], with policies registered in OpenDOAR (Directory of Open Access Repositories) [11]. Specifically, the preservation policy includes indefinite preservation of the original data with format migration to ensure continued readability and accessibility. In addition, and for convenience, these data are also hosted in the *Giga*DB repository [12].

## Methods

### Histology

Details of the mouse strains used, histological sectioning, and staining are provided by Kaufman (1994) [1]. Briefly, the embryos were “isolated from spontaneously cycling (C57BL X CBA) F1 hybrid females that had been previously mated to genetically similar F1 hybrid males.” The embryos were fixed, dehydrated, embedded in paraffin wax, and sectioned at 7-micron thickness. The mounted sections were then stained with haematoxylin and eosin.

### Slide digitization

Digitization of the original histology slides was accomplished using the Olympus DotSlide slide scanner system. Using a × 20 objective lens, this generated full-colour images with a pixel resolution of 0.34 microns. Calibration was accomplished as part of the digitization process, allowing the inclusion of scale bars and the option to measure the distance between 2 points. In 2 instances (Plate 5 and Plate 14), the original sections could not be sourced and were presumed lost. In these instances, the original photographic negatives were used in place of the original slides to generate cellular-resolution grey-scale images.

### Annotation and linking to the EMAPA ontology

Annotation was accomplished using a manual procedure whereby “flags” were positioned on points corresponding to the matching points as used in each plate in the book. The flags were placed using an editor’s version of the eHistology interface [7]. Each flag was linked to the anatomical term or phrase used in the book and also an EMAPA ontology term and an associated Wikipedia link. There were more than 10 000 flag labels used to annotate the eHistology sections, and linking them to EMAPA IDs was achieved through a combination of string matching and manual assignment of terms [13]. Linking to Wikipedia was accomplished using a manual process that utilized parent terms in the *partonomic* ontology tree to find the closest match for a given anatomical term or tissue.

### eHistology viewer

Each eHistology image is described in an Edinburgh DataShare Digital Object Identifier (DOI), and this description includes the URL link to the eHistology viewer for that image. In this way, we provide a persistent means of accessing the zoom viewer for that image. An example DOI for a single high-resolution image is dx.doi.org/10.7488/ds/1232. This link resolves to a specific page at the Edinburgh Datashare web resource[14], [15], which in turn provides a link through to the current URL for the eHistology viewer.

By starting with a fully persistent DOI, the user will always be able to locate the data and is protected from any change to the hosting domain and URL of the eHistology viewer [16]. For convenience, we also provide an interactive index to the new images based on the plate and image designations of the original atlas.

### Code availability

The data are provided in open-standard tif or jpeg image formats. All metadata are in plain txt format, and the Supplementary Data are in the Microsoft Excel open xml format xlsx. The code used for the online histology viewer is provided at the ma-tech GitHub archive, and specifically we use the WlzIIPSrv tiled image server and the eAtlasViewer javascript application.

### Data records

Each record has an assigned DOI that resolves to a set of data files comprising a jpeg or tiff encoded image, Dublin core and other metadata files, and the set of annotations associated with the image. The image data volumes range up to 2 Gb, with a total volume of 118 GB for the full series in compressed “zip” format. Table 1 lists the files with each dataset. Each University of Edinburgh DataShare submission requires a subset of the Dublin Core (dublincore.org) data elements to be completed and allows a further set of optional elements; these are detailed in Table 2. Table 3 provides a partial listing of the datasets available as an example of the data content. The full listing of all 937 images is provided in the Supplementary Excel formatted data file SciDataKaufmanTable3.xlsx and corresponds to all of the histology section images of the original atlas for Plate numbers 2–41.

**Table 1:** Listing of the data files available with each dataset

File	Description
license.txt	Licence agreement for the data deposited at DataShare providing permissions for distribution and migration as needed—CC BY 4.0.
README.txt	Short description of the data and data files.
citation.txt	How to cite use of this particular image.
details.txt	The text describing the embryo taken from the matching page of the printed atlas and provided in tab-delimited form for reading into a spreadsheet programme.
image.tif/image.jpg	Full resolution tiff or jpeg formatted image of the histological section.
Image.txt	Image pixel dimensions and pixel size in microns.
terms.txt	A tab-delimited table of the annotations for this image providing the Kaufman annotations, location in the image, annotation number, EMAPA ID, and EMAPA term, with synonyms in brackets.
url.txt	Text providing the URL for the image on the emousetlas.org web resource.

**Table 2:** DataShare Dublin Core elements used for the Kaufman datasets

Element	Qualifier	DCMI	Label	Input type	Mandatory
Contributor		contributor	Depositor	name	true
Contributor	other	contributor	Funder	name	true
Creator		creator	Data Creator	name	false
Date	accessioned	Date	Date Accessioned	date	true
date	available	Date	Date Available	date	true
Identifier	citation	identifier	Citation	citation	false
Identifier	uri	identifier	Persistent Identifier	DOI/handle	true
Description	abstract	decription	Data Description (abstract)	text	false
Description	tableofcontents	decription	Data Description (TOC)	text	false
Publisher		publisher	Publisher	text	true
Relation	isversionof	isVersionOf	Relation (Is Version Of)	text	false
Relation	isreferencedby	isReferencedBy	Relation (Is Referenced By)	text	false
Subject		subject	Subject Keywords	text	false
Subject	classification	subject	Subject Classification	Controlled text	false
Title		Title	Title	text	true
Title	alternative	Title	Alternative Title	text	false
Type		Type	Type	Controlled text	true

The DCMI column provides the official Dublin Core term for the element, and Label is the heading for these data on the DataShare metadata listing.

**Table 3:** Partial list of data records for the Kaufman Atlas image set

Kaufman image	Age	Stage	Orientation	Position	Stain	DOI
Plate 02 image a	E5.5	7–8	Sagittal		H&E	http://dx.doi.org/10.7488/ds/393
Plate 02 image b	E5.5	7–8	Sagittal		H&E	http://dx.doi.org/10.7488/ds/394
Plate 02 image c	E5.5	7–8	Sagittal		H&E	http://dx.doi.org/10.7488/ds/395
Plate 02 image d	E5.5	7–8	Sagittal		H&E	http://dx.doi.org/10.7488/ds/396
Plate 03 image a	E6.5,7	9,10	Transverse	0.210	H&E	http://dx.doi.org/10.7488/ds/397
Plate 03 image b	E6.5,7	9,10	Transverse	0.370	H&E	http://dx.doi.org/10.7488/ds/398
Plate 03 image c	E6.5,7	9,10	Transverse	0.590	H&E	http://dx.doi.org/10.7488/ds/399
.	.	.	.	.	.	.
.	.	.	.	.	.	.
Full table in Supplementary Excel file SciDataKaufmanTable3.xlsx						
.	.	.	.	.	.	.
.	.	.	.	.	.	.
.	.	.	.	.	.	.
Plate 40l image b	E17.5	26	Transverse	0.888	H&E	http://dx.doi.org/10.7488/ds/1301
Plate 40l image c	E17.5	26	Transverse	0.903	H&E	http://dx.doi.org/10.7488/ds/1302
Plate 40l image d	E17.5	26	Transverse	0.921	H&E	http://dx.doi.org/10.7488/ds/1303
Plate 40l image e	E17.5	26	Transverse	0.948	H&E	http://dx.doi.org/10.7488/ds/1304
Plate 41 image 41	E17.5	26	Sagittal	0.496	H&E	http://dx.doi.org/10.7488/ds/1305

The DOI resolves to a dataset of image data, metadata, and annotations, which can be downloaded individually or combined. Image volumes range up to about 2 Gb, with a total volume for the full set of compressed zip files of 118 Gb. The Position column gives an estimate of the relative distance through the embryo of the individual histology section. The values are between 0 and 1, corresponding to the proportionate distance left-right (sagittal sections), cranial-caudal (transverse sections), and dorsal-ventral (coronal sections).

## Technical Validation

The Images and associated data are all validated against the published atlas, which provides the detail of the genotype, defines the histological protocols, and establishes the correct staging of each embryo against the Theiler criteria. The section images used in the book are from specific tissue sections identified on the sets of microscope slides stored at the MRC Human Genetics Unit at the IGMM, University of Edinburgh. Each section was scanned digitally, then checked by a second curator to ensure validity. The annotations were originally captured using optical character recognition, and the text and spelling were checked by a second curator. All the end-point locations for the annotation terms have been double-checked, and a series of quality control steps have meant that inspection of the whole dataset has not revealed any errors.

## Usage Notes

There are no constraints on the use of the images and associated data. The Supplementary Data file lists all samples and assays—1 for each section image—and also a “source,” which is the embryonic mouse specimen used by Kaufman in producing the histological sections. The “source” can be used to identify the set of physical glass slides, archived with the Centre for Research Collections of the University of Edinburgh, on which each histological section can be found. In principle, it is possible to obtain further images of the same or other sections in the series. “Age” is defined in embryonic days post-coitum, and “stage” refers to Theiler stage, a morphological staging system used to further define mouse embryo development. “Position” describes the relative position of the section in the embryo, with 0 representing, e.g., the most cranial section in a transverse series and 1 denoting the most caudal section. We additionally include details of the pixel resolution of each image, enabling accurate measurements to be made on each high-resolution embryo atlas image.

## Availability of source code and requirements

Project name: eHistology

Project home page: http://www.emouseatlas.org/emap/eHistology/ [RRID:SCR_015887]; https://github.com/ma-tech/eHistologyWebapp

Operating system(s): platform independent

Programming language: The IIP Image server is a Fast CGI module written in C^++^.

Other requirements: The IIP Image server can be embedded within a host web server such as Apache, Lighttpd, MyServer, or Nginx.

License: software—GNU General Public License v2.0; Image Data—CC BY 4.0

## Availability of supporting data

The image datasets described in this article are available at “Edinburgh DataShare” repository as Plates 02–15 (early gestation; E5.5-E8.5), Plates 16–29 (mid-gestation; E8.5-E13.0), Plates 30–41 (late gestation; E13.5-E17.5), and Plates S1-S6 (coronal supplement; E11.5-E15.5) [17]. Each image dataset is provided with all the information needed to generate the annotated views shown on the eHistology website. The data are provided as a series of files, including details.txt—a tab-delimited file with the embryo text description that appears at the top of the printed atlas page; image.jpg—full resolution jpeg image of the histological section (in some cases, this is a tif image); terms.txt—a tab-delimited file with the annotations for this image, including location of point annotation (x, y coordinates); and EMAPA ID and EMAPA terms associated with that point. Edinburgh DataShare has provided data DOIs for each of these image datasets, and links to these data DOIs are additionally hosted in *Giga*DB. An archival copy of the GitHub repository with software tools for the eHistology application is additionally available in *Giga*DB [12].

## Abbreviations

DOI: Digital Object Identifier; H&E: haematoxylin and eosin; MRC: Medical Research Council; EMAPA: Edinburgh Mouse Atlas Project Anatomy.

## Competing interests

The authors declare they have no competing interests in the publication of this data and manuscript. The new high-resolution images and the associated image coordinates for each annotation are fully freely available under a Creative Commons CC BY 4.0 licence. We have an agreement with Elsevier to present the images in a form similar to the original atlas plate layout on the eHistology web resource. However, this does not impact reuse of the image data and annotation described in this manuscript.

## Funding

The MRC Human Genetics Unit Mouse Atlas Programme was core-funded by the Medical Research Council (Awardee R.A. Baldock).

## Authors’ contributions

R.A.B. leads the Mouse Atlas programme that generated these datasets, designed the dataset submissions to the DataShare system, and wrote the scripts that provided the upload formats needed for batch ingest to DataShare. He also wrote the first draft of the manuscript.

C.A. is the senior editor for the Mouse Atlas databases, cowrote the manuscript, and has performed much of the quality control on the datasets.

## Supplementary Material

GIGA-D-17-00086_Original_Submission.pdf

GIGA-D-17-00086_Revision_1.pdf

Response_to_Reviewer_Comments_Original_Submission.pdf

Reviewer_1_Report_(Original_Submission).pdf -- Melissa Clarkson 03 May 2017 Reviewed

Reviewer_2_Report_(Original_Submission).pdf -- Michael Hortsch 09 May 2017 Reviewed

Supplemental material
