# Supplementary material for: eHistology image and annotation data from the Kaufman Atlas of Mouse Development
Source: Gigascience. 2017 Dec 20;7(2):gix131. doi: 10.1093/gigascience/gix131 (PMC5827353; doi:10.1093/gigascience/gix131)
Supplement: GIGA-D-17-00086_Original_Submission.pdf [file gix131_giga-d-17-00086_original_submission.pdf]

GigaScience

eHistology image and annotation data from the Kaufman Atlas of Mouse Development

--Manuscript Draft--

**Manuscript Number:**	GIGA-D-17-00086
**Full Title:**	eHistology image and annotation data from the Kaufman Atlas of Mouse Development
**Article Type:**	Data Note
**Funding Information:**	Medical Research Council / Prof Richard A Baldock
**Abstract:**	The "Atlas of Mouse Development" by Kaufman is a classic paper atlas that is the de facto standard for the definition of mouse embryo anatomy in the context of standard histological images. We have re-digitised the original H&E stained tissue sections used for the book at high resolution and transferred the hand-drawn annotations to digital form. We have augmented the annotations with standard ontological assignments (EMAPA anatomy) and made the data freely available via an online viewer (eHistology) and from the University of Edinburgh DataShare archive. The dataset captures and preserves the definitive anatomical knowledge of the original atlas, provides a core image set for deeper community annotation and teaching, and delivers a unique high-quality set of high-resolution histological images through mammalian development for manual and automated analysis.
**Corresponding Author:**	Chris Armit<br><br>UNITED KINGDOM
**Corresponding Author Secondary Information:**	
**Corresponding Author's Institution:**	
**Corresponding Author's Secondary Institution:**	
**First Author:**	Chris Armit
**First Author Secondary Information:**	
**Order of Authors:**	Chris Armit
	Richard A Baldock
**Order of Authors Secondary Information:**	
**Opposed Reviewers:**	
**Additional Information:**	
**Question**	**Response**
Are you submitting this manuscript to a special series or article collection?	No
**Experimental design and statistics**<br><br>Full details of the experimental design and statistical methods used should be given in the Methods section, as detailed in our Minimum Standards Reporting Checklist. Information essential to interpreting the data presented should be made available in the figure legends.<br><br>Have you included all the information requested in your manuscript?	Yes

Powered by Editorial Manager® and ProduXion Manager® from Aries Systems Corporation

**Resources**<br><br>A description of all resources used, including antibodies, cell lines, animals and software tools, with enough information to allow them to be uniquely identified, should be included in the Methods section. Authors are strongly encouraged to cite Research Resource Identifiers (RRIDs) for antibodies, model organisms and tools, where possible.<br><br>Have you included the information requested as detailed in our Minimum Standards Reporting Checklist?	Yes
**Availability of data and materials**<br><br>All datasets and code on which the conclusions of the paper rely must be either included in your submission or deposited in publicly available repositories (where available and ethically appropriate), referencing such data using a unique identifier in the references and in the "Availability of Data and Materials" section of your manuscript.<br><br>Have you have met the above requirement as detailed in our Minimum Standards Reporting Checklist?	Yes

Manuscript
Click here to download Manuscript GigaScience_01_Kaufman_Final.pdf

Title

eHistology image and annotation data from the Kaufman Atlas of Mouse Development

Authors

Richard A Baldock[1] and Chris Armit[1]

Affiliations

1. MRC Human Genetics Unit, Institute of Genomic and Molecular Medicine, University of Edinburgh, Crewe Road, Edinburgh EH4 2XU, UK

corresponding author: Richard Baldock (Richard.Baldock@igmm.ed.ac.uk)

Abstract

The "Atlas of Mouse Development" by Kaufman is a classic paper atlas that is the *de facto* standard for the definition of mouse embryo anatomy in the context of standard histological images. We have re-digitised the original H&E stained tissue sections used for the book at high resolution and transferred the hand-drawn annotations to digital form. We have augmented the annotations with standard ontological assignments (EMAPA anatomy) and made the data freely available via an online viewer (eHistology) and from the University of Edinburgh DataShare archive. The dataset captures and preserves the definitive anatomical knowledge of the original atlas, provides a core image set for deeper community annotation and teaching, and delivers a unique high-quality set of high-resolution histological images through mammalian development for manual and automated analysis.

Background & Summary

The "Atlas of Mouse Development" (Kaufman 1994) is a book detailing the anatomy of mouse embryo development and stands as the definitive work in the field. The atlas is based on a lifetime of work by Kaufman who established a unique set of histological sections of about 450 mouse embryos, many of which are full serial section-series, from which he selected carefully staged samples for the histological images within the book. The combination of the histological section series and the printed book represent a unique resource and captures the current understanding of classical mouse anatomy. In taxonomic terms these physical sections are the *reference specimens* for the definition of mouse embryo anatomy and the digitised images with the associated annotations are a digital *holotype* for the definition of anatomical terms and the progression of mouse embryo development. In addition the paper atlas has given rise to the Mouse Atlas programme in Edinburgh (Richardson *et al*, 2014) and to the EMAPA mouse anatomy ontology (Hayamizu *et al*, 2013; Hayamizu *et al*, 2015). The original index for the book was used to develop the primary list of anatomical terms in the ontology, and EMAPA is now recognised as the standard mouse embryo ontology used to annotate mouse embryo data including embryo phenotype data (Brown and Moore, 2012; Mohun *et al*, 2013).

In generating the eHistology Atlas, new images of the histological sections were acquired at high resolution, and the annotations have been transferred to a database. These images and annotations are now freely available from the eMouseAtlas web resource as eHistology (see figure 1) and have been described by Graham et al (2015). Here we describe the dataset of the 937 high-resolution histology images with anatomy annotations and how they have been made available for further study and analysis.

The motivation for the eHistology resource was to capture the anatomical knowledge in a permanently accessible open and digital form delivered with a viewer providing a view of the underlying histology data not possible in the printed atlas. The high-resolution images

provide a rich resource of carefully staged mouse histology, which could be used for deeper analysis of tissue development and as a teaching resource. See figure 1 for an illustration of the resolution now available for these images.

All the data is available under a creative commons licence (CC BY 4.0). In the future, we envisage the annotations being extended on a tissue-by-tissue basis through community curation. The histology viewer is open-source and is available from the Mouse Atlas technical GitHub repository (github.com/ma-tech).

Providing for a secure (in terms of data preservation) and long-term accessibility for research data is a difficult problem. A recent study of the longevity of 375 biomedical resources/databases (Attwood et al, 2015) available on the web in 1997 found that 62.3% had ceased to be available, 14.4% were static and only 23.3% available as an active resource. The authors concluded that survival depended primarily on Institutional interest and that a strategy dependent on external funding will very likely fail. To ensure long-term preservation of the image data and supporting annotations, we have therefore registered this dataset with the University of Edinburgh *DataShare* (http://datashare.is.ed.ac.uk/) repository (Rice 2014) with policies registered in OpenDOAR (Directory of Open Access Repositories, http://www.opendoar.org/find.php?rID=1176&format=full). Specifically the preservation policy includes indefinite preservation of the original data with format migration to ensure continued readability and accessibility. We suggest that the longevity provided by the University of Edinburgh will exceed any other option not associated with a similar institution.

Methods

Histology

Details of the mouse strains used, histological sectioning and staining are provided by Kaufman (1994). Briefly the embryos were "isolated from spontaneously cycling (C57BL X CBA) F1 hybrid females that had been previously mated to genetically similar F1 hybrid males". The embryos were fixed, dehydrated, embedded in paraffin wax and sectioned at seven-micron thickness. The mounted sections were then stained with haematoxylin and eosin.

Slide digitization

Digitization of the original histology slides was accomplished using the Olympus DotSlide slide scanner system. Using a ×20 objective lens, this generated full colour images with a pixel resolution of 0.34 microns. Calibration was accomplished as part of the digitization process, allowing the inclusion of scale-bars and the option to measure the distance between two points. In two instances (Plate 5 and Plate 14) the original sections could not be sourced and were presumed lost. In these instances the original photographic negatives were used in place of the original slides to generate cellular-resolution grey-scale images.

Annotation and linking to the EMAPA ontology

Annotation was accomplished using a manual procedure whereby "flags" were positioned on points corresponding to the matching points as used in each plate in the book. The flags were placed using a editors version of the eHistology interface (Graham *et al*, 2015). Each flag was linked to the anatomical term or phrase used in the book and also an EMAPA ontology term and an associated Wikipedia link. There were over 10,000 flag labels used to annotate the eHistology sections, and linking them to EMAPA IDs was achieved through a combination of string matching and manual assignment of terms (Richardson et al., 2015). Linking to Wikipedia was accomplished using a manual process that utilized parent terms in the *partonomic* ontology tree to find the closest match for a given anatomical term or tissue.

Code availability

All code used to generate this dataset is available open-source from the ma-tech repository at GitHub.

Data Records

Each record has an assigned Digital Object Identifier (DOI) that resolves to a set of data files comprising a jpeg or tiff encoded image, Dublin core and other metadata files and the set of annotations associated with the image. The image data volumes range up to 2Gb with a total volume of 118GB for the full series in compressed "zip" format. Table 1 lists the files with each dataset. Each University of Edinburgh DataShare submission requires a subset of the Dublin Core [dublincore.org] data elements to be completed and allows a further set of optional elements these are detailed in table 2. Table 3 provides a partial listing of the datasets available as an example of the data content. The full listing of all 937 images is provided in the supplementary Excel formatted data file SciDataKaufmanTable3.xlsx and corresponds to all of the histology section images of the original atlas for Plate numbers 2-41.

Table 1. Listing of the data files available with each dataset.

File	Description
**license.txt**	Licence agreement for the data deposited at DataShare providing permissions for distribution and migration as needed - CC BY 4.0.
**README.txt**	Short description of the data and data files.
**citation.txt**	How to cite use of this particular image.
**details.txt**	The text describing the embryo taken from the matching page of the printed atlas and provided in tab-delimited form for reading into a spreadsheet programme.
**image.tif/image.jpg**	Full resolution tiff or jpeg formatted image of the histological section.
**Image.txt**	Image pixel dimensions and pixel size in microns.
**terms.txt**	A tab-delimited table of the annotations for this image providing the Kaufman annotations, location in the image, annotation number, EMAPA ID and EMAPA term with synonyms in brackets.
**url.txt**	Text providing the URL for the image on the emousetlas.org web resource.

Table 2. DataShare Dublin Core elements used for the Kaufman datasets. The DCMI column provides the official Dublin Core term for the element, the Label is the heading for these data on the DataShare metadata listing.

Element	Qualifier	DCMI	Label	Input Type	Mandatory
**contributor**		contributor	Depositor	name	true
**contributor**	other	contributor	Funder	name	true
**creator**		creator	Data Creator	name	false
**date**	accessioned	date	Date Accessioned	date	true
**date**	available	date	Date Available	date	true
**identifier**	citation	identifier	Citation	citation	false
**identifier**	uri	identifier	Persistent Identifier	DOI/handle	true
**description**	abstract	decription	Data Description (abstract)	text	false
**description**	tableofcontents	decription	Data Description (TOC)	text	false
**publisher**		publisher	Publisher	text	true
**relation**	isversionof	isVersionOf	Relation (Is Version Of)	text	false
**relation**	isreferencedby	isReferencedBy	Relation (Is Referenced By)	text	false
**subject**		subject	Subject Keywords	text	false
**subject**	classification	subject	Subject Classification	Controlled text	false
**title**		title	Title	text	true
**title**	alternative	title	Alternative Title	text	false
**type**		type	Type	Controlled text	true

Table 3. Partial list of data records for the Kaufman Atlas image set. The DOI resolves to a data set of image data, metadata and annotations, which can be downloaded individually or combined. Image volumes range up to about 2Gb with a total volume for the full set of compressed zip files 118Gb. The Position column gives an estimate of the relative distance through the embryo of the individual histology section. The values are between 0 (zero) and 1 (one) corresponding to the proportionate distance left-right (sagittal sections), cranial-caudal (transverse sections) and dorsal-ventral (coronal sections).

Kaufman Image	Age	Stage	Orientation	Position	Stain	DOI
Plate 02 image a	E5.5	7-8	Sagittal		H&E	http://dx.doi.org/10.7488/ds/393
Plate 02 image b	E5.5	7-8	Sagittal		H&E	http://dx.doi.org/10.7488/ds/394
Plate 02 image c	E5.5	7-8	Sagittal		H&E	http://dx.doi.org/10.7488/ds/395
Plate 02 image d	E5.5	7-8	Sagittal		H&E	http://dx.doi.org/10.7488/ds/396
Plate 03 image a	E6.5,7	9,10	Transverse	0.210	H&E	http://dx.doi.org/10.7488/ds/397
Plate 03 image b	E6.5,7	9,10	Transverse	0.370	H&E	http://dx.doi.org/10.7488/ds/398
Plate 03 image c	E6.5,7	9,10	Transverse	0.590	H&E	http://dx.doi.org/10.7488/ds/399
.	.	.	.	.	.	.
.	.	.	.	.	.	.
Full table in supplementary Excel file SciDataKaufmanTable3.xlsx						
.	.	.	.	.	.	.
.	.	.	.	.	.	.
.	.	.	.	.	.	.
Plate 40l image b	E17.5	26	Transverse	0.888	H&E	http://dx.doi.org/10.7488/ds/1301
Plate 40l image c	E17.5	26	Transverse	0.903	H&E	http://dx.doi.org/10.7488/ds/1302
Plate 40l image d	E17.5	26	Transverse	0.921	H&E	http://dx.doi.org/10.7488/ds/1303
Plate 40l image e	E17.5	26	Transverse	0.948	H&E	http://dx.doi.org/10.7488/ds/1304
Plate 41 image 41	E17.5	26	Sagittal	0.496	H&E	http://dx.doi.org/10.7488/ds/1305

Technical Validation

The Images and associated data are all validated against the published atlas which provides the detail of the genotype, defines the histological protocols, and establishes the correct staging of each embryo against the Theiler criteria. The section images used in the book are from specific tissue sections identified on the sets of microscope slides stored at the MRC Human Genetics Unit at the IGMM, University of Edinburgh. Each section was scanned digitally then checked by a second curator to ensure validity. The annotations were originally captured using optical character recognition (OCR) and the text and spelling checked by a second curator. All the end-point locations for the annotation terms have been double-checked and a series of quality control steps have meant that inspection of the whole data set has not revealed any errors.

Usage Notes

There are no constraints on the use of the images and associated data. The data descriptors file lists all samples and assays, one for each section image, and also a “source” which for this material is the original embryo sample used by Kaufman in producing the histological sections. The data descriptor file lists the embryo code that Kaufman provided in his paper atlas and which can be used to identify the set of physical glass slides help by the University of Edinburgh on which each histological section can be found. In principle it is possible to obtain further images of the same or other sections in the series.

Acknowledgements

The authors would like to acknowledge the critical contribution to the collection, collation and curation of these data by Liz Graham and Julie Moss and to the development of the data visualisation aspects by Nick Burton. All these are co-authors on the key citation for this work [Graham et al, 2015]. The authors would also like to thank the support of the Edina DataShare team, in particular Pauline Ward and Robin Rice. Finally this work has been funded as part of the MRC Funded core-funded Mouse Atlas Programme at the MRC Human Genetics Unit Edinburgh.

Author contributions

RAB leads the Mouse Atlas programme that generated these datasets, designed the dataset submissions to the DataShare system and wrote the scripts providing the upload formats needed for batch ingest to DataShare. He also wrote the first draft of the manuscript.

CA is the senior editor for the Mouse Atlas databases, co-wrote the manuscript and has performed much of the quality control on the datasets.

Competing interests

The authors declare they have no competing interests in the publication of this data and manuscript.

Figures (optional)

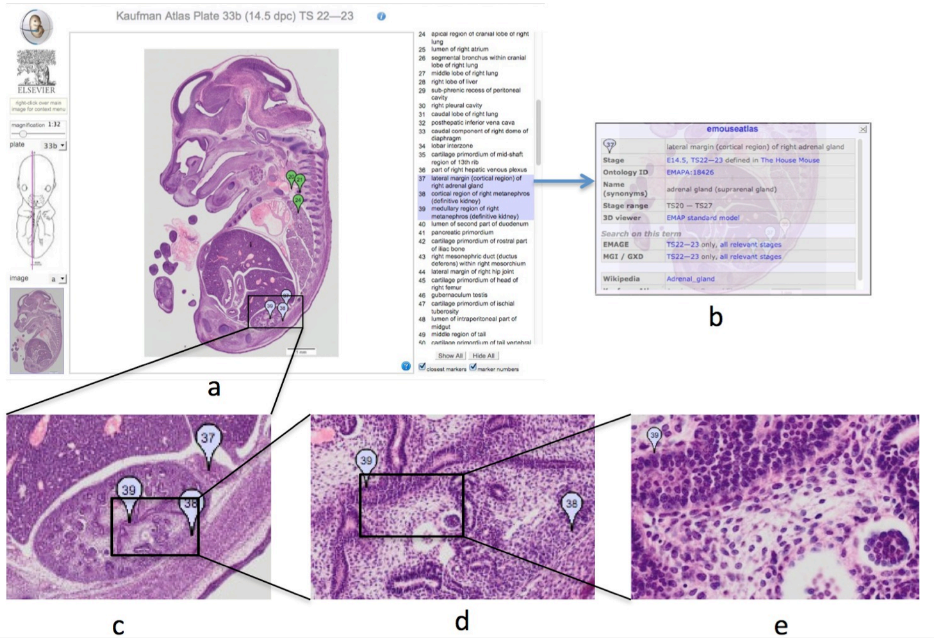

Figure 1. Kaufman Atlas eHistology Viewer. a) screen shot showing the user interface with annotation list. The 3 selected terms showing as a blue "flags" number 37, 38, 39 are over the developing kidney. b) the pop-up dialogue with extra information on selecting the "37". c), d), e) progressively higher resolution images corresponding to zooming-in on the image. At full resolution the pixel spacing is 0.34 x 0.34 microns and reveals cellular architecture and arrangements.

Figure Legends (optional)

Figure 1. Kaufman Atlas eHistology Viewer. a) screen shot showing the user interface with annotation list. The 3 selected terms showing as a blue "flags" number 37, 38, 39 are over the developing kidney. b) The pop-up dialog with extra information on selecting the "37". c), d), e) progressively higher resolution images corresponding to zooming-in on the image. At full resolution the pixel spacing is 0.34 x 0.34 microns and reveals cellular architecture and arrangements.

Tables

Table legends from above:

Table 1. Listing of the data files available with each dataset.

Table 2. DataShare Dublin Core elements used for the Kaufman datasets. The DCMI column provides the official Dublin Core term for the element, the Label is the heading for this data on the DataShare metadata listing.

Table 3. Complete list of data records for the Kaufman Atlas image set. The DOI resolves to a data set of image data, metadata and annotations, which can be downloaded individually or combined. Image volumes range up to about 2Gb with a total volume for the full set of compressed zip files 118Gb. The Position column gives an estimate of the relative distance through the embryo of the individual histology section. The values are between 0 (zero) and 1 (one) corresponding to the proportionate distance left-right (sagittal sections), cranial-caudal (transverse sections) and dorsal-ventral (coronal sections).

References

1. Graham, E., Moss, J., Burton, N., Roochun, Y., Armit, C., Lorna Richardson, L. & Baldock, R. The atlas of mouse development eHistology resource. *Development* **142**, 1909-1911 (2015).
2. Richardson L, Moss J, Graham E, Burton N, Armit C, Baldock R (2015) Developing the eHistology Atlas. *Database (Oxford) 2015.* . **bav105** doi: 10.1093/database/bav105
3. Kaufman, MH. The Atlas of Mouse Development**.** *Elsevier Academic Press, Amsterdam, The Netherlands*. (1994).
4. Hayamizu, TF., Wicks, MN., Davidson, DR., Burger, A., Ringwald, M. & Baldock, RA. EMAP/EMAPA ontology of mouse developmental anatomy: 2013 update*, Journal of Biomedical Semantics.* **4:15**. (2013).
5. Hayamizu, TF., Baldock, RA. & Ringwald, M., Mouse anatomy ontologies: enhancements and tools for exploring and integrating biomedical data, *Mamm Genome*. **26**: 422–430 (2015).
6. Brown, SD. & Moore, MW. Towards an encyclopaedia of mammalian gene function: the International Mouse Phenotyping Consortium*. Dis Model Mech* **5**(3), 289–92 (2012).
7. Mohun, T., Adams, DJ., Baldock, R., Bhattacharya, S., Copp, AJ., Hemberger, M., Houart, C., Hurles, ME., Robertson, E., Smith, JC., Weaver, T. & Weninger, W. Deciphering the Mechanisms of Developmental Disorders (DMDD): a new programme for phenotyping embryonic lethal mice. *Disease Models & Mechanisms* **6**, 562-566 (2013).
8. Attwood, TK., Agit, B. & Ellis, ABM. Longevity of Biological Databases. *EMBnet.journal* **21,** e803 (2015). http://dx.doi.org/10.14806/ej.21.0.803
9. Rice, R. Edinburgh datashare – reflections from a data repository manager. *Bull. Assoc. Information Science and technology* **40(2)** 39-40 (2014). DOI: 10.1002/bult.2014.1720400212

10. Richardson, L., Venkataraman, S., Stevenson, P., Yang, Y., Moss, J., Graham, L., Burton, N., Hill, B., Rao, J., Baldock, RA. & Armit, C. EMAGE mouse embryo spatial gene expression database: (2014 update). *Nucleic Acids Res*. **42(1)** D835-44 (2014). doi: 10.1093/nar/gkt1155

Data Citations

Bibliographic information for the data records described in the manuscript.

1. Graham, E., Moss, J., Burton, N., Roochun, Y., Armit, C., Lorna Richardson, L. & Baldock, R. *DataShare* http://dx.doi.org/10.7488/ds/393 to http://dx.doi.org/10.7488/ds/1305

 Click here to download Table SciDataKaufmanTable3.xlsx

eHistology image and annotation data from the Kaufman Atlas of Mouse D

Kaufman Image	Age	Stage	Orientation	Position	Stain
Plate 02 image a	E5.5	7	Sagittal		H&E
Plate 02 image b	E5.5	7	Sagittal		H&E
Plate 02 image c	E5.5	7	Sagittal		H&E
Plate 02 image d	E5.5	7	Sagittal		H&E
Plate 03 image a	E6.5,7	9,10	Transverse	0.21	H&E
Plate 03 image b	E6.5,7	9,10	Transverse	0.37	H&E
Plate 03 image c	E6.5,7	9,10	Transverse	0.59	H&E
Plate 03 image d	E6.5,7	9,10	Transverse	0.75	H&E
Plate 03 image e	E6.5,7	9,10	Transverse	0.91	H&E
Plate 03 image f	E6.5,7	9,10	Transverse	0.136	H&E
Plate 03 image g	E6.5,7	9,10	Transverse	0.297	H&E
Plate 03 image h	E6.5,7	9,10	Transverse	0.466	H&E
Plate 03 image i	E6.5,7	9,10	Transverse	0.661	H&E
Plate 03 image j	E6.5,7	9,10	Transverse	0.847	H&E
Plate 03 image k	E6.5,7	9,10	Transverse	0.221	H&E
Plate 03 image l	E6.5,7	9,10	Transverse	0.371	H&E
Plate 03 image m	E6.5,7	9,10	Transverse	0.614	H&E
Plate 03 image n	E6.5,7	9,10	Transverse	0.729	H&E
Plate 03 image o	E6.5,7	9,10	Transverse	0.8	H&E
Plate 04 image a	E7	10	Transverse	0	H&E
Plate 04 image b	E7	10	Transverse	0.11	H&E
Plate 04 image c	E7	10	Transverse	0.294	H&E
Plate 04 image d	E7	10	Transverse	0.412	H&E
Plate 04 image e	E7	10	Transverse	0.515	H&E
Plate 04 image f	E7	10	Transverse	0.61	H&E
Plate 04 image g	E7	10	Transverse	0.706	H&E
Plate 04 image h	E7	10	Transverse	0.794	H&E
Plate 04 image i	E7	10	Transverse	0.136	H&E
Plate 04 image j	E7	10	Transverse	0.235	H&E
Plate 04 image k	E7	10	Transverse	0.341	H&E
Plate 04 image l	E7	10	Transverse	0.455	H&E
Plate 04 image m	E7	10	Transverse	0.545	H&E
Plate 04 image n	E7	10	Transverse	0.644	H&E
Plate 04 image o	E7	10	Transverse	0.75	H&E
Plate 04 image p	E7	10	Transverse	0.856	H&E
Plate 05 image a	E7	10-Nov	Sagittal	0.31	H&E
Plate 05 image b	E7	10-Nov	Sagittal	0.345	H&E

Plate 05 image c	E7	10-Nov	Sagittal	0.493	H&E
Plate 05 image d	E7	10-Nov	Sagittal	0.599	H&E
Plate 05 image e	E7	10-Nov	Sagittal	0.655	H&E
Plate 05 image f	E7	10-Nov	Sagittal	0.31	H&E
Plate 05 image g	E7	10-Nov	Sagittal	0.366	H&E
Plate 05 image h	E7	10-Nov	Sagittal	0.493	H&E
Plate 05 image i	E7	10-Nov	Sagittal	0.599	H&E
Plate 05 image j	E7	10-Nov	Sagittal	0.662	H&E
Plate 06 image a	E7	11	Transverse	0.065	H&E
Plate 06 image b	E7	11	Transverse	0.153	H&E
Plate 06 image c	E7	11	Transverse	0.218	H&E
Plate 06 image d	E7	11	Transverse	0.282	H&E
Plate 06 image e	E7	11	Transverse	0.398	H&E
Plate 06 image f	E7	11	Transverse	0.509	H&E
Plate 06 image g	E7	11	Transverse	0.606	H&E
Plate 06 image h	E7	11	Transverse	0.639	H&E
Plate 06 image i	E7	11	Transverse	0.694	H&E
Plate 06 image j	E7	11	Transverse	0.792	H&E
Plate 06 image k	E7	11	Transverse	0.861	H&E
Plate 06 image l	E7	11	Transverse	0.935	H&E
Plate 07 image a	E7.5	11	Transverse	0.021	H&E
Plate 07 image b	E7.5	11	Transverse	0.063	H&E
Plate 07 image c	E7.5	11	Transverse	0.141	H&E
Plate 07 image d	E7.5	11	Transverse	0.229	H&E
Plate 07 image e	E7.5	11	Transverse	0.307	H&E
Plate 07 image f	E7.5	11	Transverse	0.359	H&E
Plate 07 image g	E7.5	11	Transverse	0.406	H&E
Plate 07 image h	E7.5	11	Transverse	0.453	H&E
Plate 07 image i	E7.5	11	Transverse	0.49	H&E
Plate 07 image j	E7.5	11	Transverse	0.552	H&E
Plate 07 image k	E7.5	11	Transverse	0.589	H&E
Plate 07 image l	E7.5	11	Transverse	0.63	H&E
Plate 07 image m	E7.5	11	Transverse	0.682	H&E
Plate 07 image n	E7.5	11	Transverse	0.719	H&E
Plate 07 image o	E7.5	11	Transverse	0.755	H&E
Plate 07 image p	E7.5	11	Transverse	0.807	H&E
Plate 08 image a	E7.5	11	Transverse	0.057	H&E
Plate 08 image b	E7.5	11	Transverse	0.131	H&E
Plate 08 image c	E7.5	11	Transverse	0.245	H&E
Plate 08 image d	E7.5	11	Transverse	0.314	H&E
Plate 08 image e	E7.5	11	Transverse	0.367	H&E

Plate 08 image f	E7.5	11	Transverse	0.42	H&E
Plate 08 image g	E7.5	11	Transverse	0.543	H&E
Plate 08 image i	E7.5	11	Transverse	0.633	H&E
Plate 08 image j	E7.5	11	Transverse	0.735	H&E
Plate 08 image k	E7.5	11	Transverse	0.8	H&E
Plate 08 image l	E7.5	11	Transverse	0.857	H&E
Plate 08 image l	E7.5	11	Transverse	0.927	H&E
Plate 09 image a	E7.5-8	12	Transverse	0.041	H&E
Plate 09 image b	E7.5-8	12	Transverse	0.208	H&E
Plate 09 image c	E7.5-8	12	Transverse	0.282	H&E
Plate 09 image d	E7.5-8	12	Transverse	0.347	H&E
Plate 09 image e	E7.5-8	12	Transverse	0.392	H&E
Plate 09 image f	E7.5-8	12	Transverse	0.42	H&E
Plate 09 image g	E7.5-8	12	Transverse	0.457	H&E
Plate 09 image h	E7.5-8	12	Transverse	0.49	H&E
Plate 09 image i	E7.5-8	12	Transverse	0.563	H&E
Plate 09 image j	E7.5-8	12	Transverse	0.629	H&E
Plate 09 image k	E7.5-8	12	Transverse	0.682	H&E
Plate 09 image l	E7.5-8	12	Transverse	0.735	H&E
Plate 09 image m	E7.5-8	12	Transverse	0.784	H&E
Plate 09 image n	E7.5-8	12	Transverse	0.841	H&E
Plate 09 image o	E7.5-8	12	Transverse	0.898	H&E
Plate 09 image p	E7.5-8	12	Transverse	0.943	H&E
Plate 10 image a	E8	12	Transverse	0.014	H&E
Plate 10 image b	E8	12	Transverse	0.079	H&E
Plate 10 image c	E8	12	Transverse	0.116	H&E
Plate 10 image d	E8	12	Transverse	0.191	H&E
Plate 10 image e	E8	12	Transverse	0.247	H&E
Plate 10 image f	E8	12	Transverse	0.312	H&E
Plate 10 image g	E8	12	Transverse	0.377	H&E
Plate 10 image h	E8	12	Transverse	0.433	H&E
Plate 10 image i	E8	12	Transverse	0.465	H&E
Plate 10 image j	E8	12	Transverse	0.516	H&E
Plate 10 image k	E8	12	Transverse	0.553	H&E
Plate 10 image l	E8	12	Transverse	0.614	H&E
Plate 10 image m	E8	12	Transverse	0.642	H&E
Plate 10 image n	E8	12	Transverse	0.684	H&E
Plate 10 image o	E8	12	Transverse	0.721	H&E
Plate 10 image p	E8	12	Transverse	0.777	H&E
Plate 10 image q	E8	12	Transverse	0.819	H&E
Plate 10 image r	E8	12	Transverse	0.851	H&E

Plate 10 image s	E8	12	Transverse	0.893	H&E
Plate 10 image t	E8	12	Transverse	0.94	H&E
Plate 11 image a	E8	12	Transverse	0.081	H&E
Plate 11 image b	E8	12	Transverse	0.154	H&E
Plate 11 image c	E8	12	Transverse	0.201	H&E
Plate 11 image d	E8	12	Transverse	0.268	H&E
Plate 11 image e	E8	12	Transverse	0.302	H&E
Plate 11 image f	E8	12	Transverse	0.376	H&E
Plate 11 image g	E8	12	Transverse	0.416	H&E
Plate 11 image h	E8	12	Transverse	0.49	H&E
Plate 11 image i	E8	12	Transverse	0.597	H&E
Plate 11 image j	E8	12	Transverse	0.671	H&E
Plate 11 image k	E8	12	Transverse	0.711	H&E
Plate 11 image l	E8	12	Transverse	0.745	H&E
Plate 11 image m	E8	12	Transverse	0.846	H&E
Plate 11 image n	E8	12	Transverse	0.899	H&E
Plate 11 image o	E8	12	Transverse	0.94	H&E
Plate 12 image a	E8-8.5	13	Transverse	0.085	H&E
Plate 12 image b	E8-8.5	13	Transverse	0.174	H&E
Plate 12 image c	E8-8.5	13	Transverse	0.239	H&E
Plate 12 image d	E8-8.5	13	Transverse	0.277	H&E
Plate 12 image e	E8-8.5	13	Transverse	0.315	H&E
Plate 12 image f	E8-8.5	13	Transverse	0.347	H&E
Plate 12 image g	E8-8.5	13	Transverse	0.376	H&E
Plate 12 image h	E8-8.5	13	Transverse	0.404	H&E
Plate 12 image i	E8-8.5	13	Transverse	0.432	H&E
Plate 12 image j	E8-8.5	13	Transverse	0.469	H&E
Plate 12 image k	E8-8.5	13	Transverse	0.516	H&E
Plate 12 image l	E8-8.5	13	Transverse	0.545	H&E
Plate 12 image m	E8-8.5	13	Transverse	0.573	H&E
Plate 12 image n	E8-8.5	13	Transverse	0.606	H&E
Plate 12 image o	E8-8.5	13	Transverse	0.638	H&E
Plate 12 image p	E8-8.5	13	Transverse	0.676	H&E
Plate 12 image q	E8-8.5	13	Transverse	0.746	H&E
Plate 12 image r	E8-8.5	13	Transverse	0.831	H&E
Plate 12 image s	E8-8.5	13	Transverse	0.887	H&E
Plate 12 image t	E8-8.5	13	Transverse	0.934	H&E
Plate 13a image a	E8-8.5	13	Transverse	0.064	H&E
Plate 13a image b	E8-8.5	13	Transverse	0.092	H&E
Plate 13a image c	E8-8.5	13	Transverse	0.161	H&E
Plate 13a image d	E8-8.5	13	Transverse	0.197	H&E

Plate 13a image e	E8-8.5	13	Transverse	0.257	H&E
Plate 13a image f	E8-8.5	13	Transverse	0.294	H&E
Plate 13a image g	E8-8.5	13	Transverse	0.339	H&E
Plate 13a image h	E8-8.5	13	Transverse	0.381	H&E
Plate 13a image i	E8-8.5	13	Transverse	0.422	H&E
Plate 13a image j	E8-8.5	13	Transverse	0.454	H&E
Plate 13a image k	E8-8.5	13	Transverse	0.486	H&E
Plate 13a image l	E8-8.5	13	Transverse	0.518	H&E
Plate 13b image a	E8-8.5	13	Transverse	0.555	H&E
Plate 13b image b	E8-8.5	13	Transverse	0.587	H&E
Plate 13b image c	E8-8.5	13	Transverse	0.624	H&E
Plate 13b image d	E8-8.5	13	Transverse	0.67	H&E
Plate 13b image e	E8-8.5	13	Transverse	0.702	H&E
Plate 13b image f	E8-8.5	13	Transverse	0.729	H&E
Plate 13b image g	E8-8.5	13	Transverse	0.766	H&E
Plate 13b image h	E8-8.5	13	Transverse	0.784	H&E
Plate 13b image i	E8-8.5	13	Transverse	0.835	H&E
Plate 13b image j	E8-8.5	13	Transverse	0.89	H&E
Plate 13b image k	E8-8.5	13	Transverse	0.945	H&E
Plate 13b image l	E8-8.5	13	Transverse	0.982	H&E
Plate 14a image a	E8-8.5	13	Transverse	0.045	H&E
Plate 14a image b	E8-8.5	13	Transverse	0.07	H&E
Plate 14a image c	E8-8.5	13	Transverse	0.103	H&E
Plate 14a image d	E8-8.5	13	Transverse	0.157	H&E
Plate 14a image e	E8-8.5	13	Transverse	0.211	H&E
Plate 14a image f	E8-8.5	13	Transverse	0.26	H&E
Plate 14a image g	E8-8.5	13	Transverse	0.281	H&E
Plate 14a image h	E8-8.5	13	Transverse	0.318	H&E
Plate 14a image i	E8-8.5	13	Transverse	0.364	H&E
Plate 14a image j	E8-8.5	13	Transverse	0.388	H&E
Plate 14a image k	E8-8.5	13	Transverse	0.417	H&E
Plate 14a image l	E8-8.5	13	Transverse	0.455	H&E
Plate 14b image a	E8-8.5	13	Transverse	0.492	H&E
Plate 14b image b	E8-8.5	13	Transverse	0.517	H&E
Plate 14b image c	E8-8.5	13	Transverse	0.545	H&E
Plate 14b image d	E8-8.5	13	Transverse	0.574	H&E
Plate 14b image e	E8-8.5	13	Transverse	0.603	H&E
Plate 14b image f	E8-8.5	13	Transverse	0.628	H&E
Plate 14b image g	E8-8.5	13	Transverse	0.661	H&E
Plate 14b image h	E8-8.5	13	Transverse	0.707	H&E
Plate 14b image i	E8-8.5	13	Transverse	0.773	H&E

Plate 14b image j	E8-8.5	13	Transverse	0.843	H&E
Plate 14b image k	E8-8.5	13	Transverse	0.913	H&E
Plate 14b image l	E8-8.5	13	Transverse	0.959	H&E
Plate 15a image a	E8.5	13-14	Transverse	0.022	H&E
Plate 15a image b	E8.5	13-14	Transverse	0.065	H&E
Plate 15a image c	E8.5	13-14	Transverse	0.105	H&E
Plate 15a image d	E8.5	13-14	Transverse	0.135	H&E
Plate 15a image e	E8.5	13-14	Transverse	0.175	H&E
Plate 15a image f	E8.5	13-14	Transverse	0.244	H&E
Plate 15a image g	E8.5	13-14	Transverse	0.28	H&E
Plate 15a image h	E8.5	13-14	Transverse	0.316	H&E
Plate 15a image i	E8.5	13-14	Transverse	0.342	H&E
Plate 15a image j	E8.5	13-14	Transverse	0.385	H&E
Plate 15a image k	E8.5	13-14	Transverse	0.425	H&E
Plate 15a image l	E8.5	13-14	Transverse	0.476	H&E
Plate 15a image m	E8.5	13-14	Transverse	0.516	H&E
Plate 15a image n	E8.5	13-14	Transverse	0.549	H&E
Plate 15a image o	E8.5	13-14	Transverse	0.578	H&E
Plate 15a image p	E8.5	13-14	Transverse	0.625	H&E
Plate 15b image a	E8.5	13-14	Transverse	0.658	H&E
Plate 15b image b	E8.5	13-14	Transverse	0.716	H&E
Plate 15b image c	E8.5	13-14	Transverse	0.793	H&E
Plate 15b image d	E8.5	13-14	Transverse	0.815	H&E
Plate 15b image e	E8.5	13-14	Transverse	0.855	H&E
Plate 15b image f	E8.5	13-14	Transverse	0.887	H&E
Plate 15b image g	E8.5	13-14	Transverse	0.909	H&E
Plate 15b image h	E8.5	13-14	Transverse	0.935	H&E
Plate 15b image i	E8.5	13-14	Transverse	0.964	H&E
Plate 15b image j	E8.5	13-14	Transverse	0.982	H&E
Plate 16a image a	E8.5-9	13-14	Transverse	0.058	H&E
Plate 16a image b	E8.5-9	13-14	Transverse	0.127	H&E
Plate 16a image c	E8.5-9	13-14	Transverse	0.189	H&E
Plate 16a image d	E8.5-9	13-14	Transverse	0.243	H&E
Plate 16a image e	E8.5-9	13-14	Transverse	0.286	H&E
Plate 16a image f	E8.5-9	13-14	Transverse	0.324	H&E
Plate 16a image g	E8.5-9	13-14	Transverse	0.351	H&E
Plate 16a image h	E8.5-9	13-14	Transverse	0.378	H&E
Plate 16a image i	E8.5-9	13-14	Transverse	0.417	H&E
Plate 16a image j	E8.5-9	13-14	Transverse	0.44	H&E
Plate 16a image k	E8.5-9	13-14	Transverse	0.467	H&E
Plate 16a image l	E8.5-9	13-14	Transverse	0.494	H&E

Plate 16b image a	E8.5-9	13-14	Transverse	0.525	H&E
Plate 16b image b	E8.5-9	13-14	Transverse	0.556	H&E
Plate 16b image c	E8.5-9	13-14	Transverse	0.583	H&E
Plate 16b image d	E8.5-9	13-14	Transverse	0.614	H&E
Plate 16b image e	E8.5-9	13-14	Transverse	0.637	H&E
Plate 16b image f	E8.5-9	13-14	Transverse	0.664	H&E
Plate 16b image g	E8.5-9	13-14	Transverse	0.699	H&E
Plate 16b image h	E8.5-9	13-14	Transverse	0.726	H&E
Plate 16b image i	E8.5-9	13-14	Transverse	0.799	H&E
Plate 16b image j	E8.5-9	13-14	Transverse	0.83	H&E
Plate 16b image k	E8.5-9	13-14	Transverse	0.88	H&E
Plate 16b image l	E8.5-9	13-14	Transverse	0.934	H&E
Plate 17 image a	E9	14	Sagittal	0.712	H&E
Plate 17 image b	E9	14	Sagittal	0.648	H&E
Plate 17 image c	E9	14	Sagittal	0.576	H&E
Plate 17 image d	E9	14	Sagittal	0.536	H&E
Plate 17 image e	E9	14	Sagittal	0.488	H&E
Plate 17 image f	E9	14	Sagittal	0.432	H&E
Plate 17 image g	E9	14	Sagittal	0.344	H&E
Plate 17 image h	E9	14	Sagittal	0.288	H&E
Plate 17 image i	E9	14	Sagittal	0.24	H&E
Plate 18a image a	E9	14	Transverse	0.041	H&E
Plate 18a image b	E9	14	Transverse	0.116	H&E
Plate 18a image c	E9	14	Transverse	0.165	H&E
Plate 18a image d	E9	14	Transverse	0.217	H&E
Plate 18a image e	E9	14	Transverse	0.247	H&E
Plate 18a image f	E9	14	Transverse	0.296	H&E
Plate 18a image g	E9	14	Transverse	0.33	H&E
Plate 18a image h	E9	14	Transverse	0.356	H&E
Plate 18a image i	E9	14	Transverse	0.378	H&E
Plate 18a image j	E9	14	Transverse	0.412	H&E
Plate 18a image k	E9	14	Transverse	0.446	H&E
Plate 18a image l	E9	14	Transverse	0.468	H&E
Plate 18a image m	E9	14	Transverse	0.491	H&E
Plate 18a image n	E9	14	Transverse	0.513	H&E
Plate 18a image o	E9	14	Transverse	0.532	H&E
Plate 18b image a	E9	14	Transverse	0.566	H&E
Plate 18b image b	E9	14	Transverse	0.581	H&E
Plate 18b image c	E9	14	Transverse	0.603	H&E
Plate 18b image d	E9	14	Transverse	0.625	H&E
Plate 18b image e	E9	14	Transverse	0.644	H&E

Plate 18b image f	E9	14	Transverse	0.667	H&E
Plate 18b image g	E9	14	Transverse	0.689	H&E
Plate 18b image h	E9	14	Transverse	0.715	H&E
Plate 18b image i	E9	14	Transverse	0.734	H&E
Plate 18b image j	E9	14	Transverse	0.764	H&E
Plate 18b image k	E9	14	Transverse	0.79	H&E
Plate 18b image l	E9	14	Transverse	0.816	H&E
Plate 18b image m	E9	14	Transverse	0.854	H&E
Plate 18b image n	E9	14	Transverse	0.891	H&E
Plate 18b image o	E9	14	Transverse	0.978	H&E
Plate 19a image a	E9.5	15	Transverse	0.047	H&E
Plate 19a image b	E9.5	15	Transverse	0.086	H&E
Plate 19a image c	E9.5	15	Transverse	0.121	H&E
Plate 19a image d	E9.5	15	Transverse	0.152	H&E
Plate 19a image e	E9.5	15	Transverse	0.179	H&E
Plate 19a image f	E9.5	15	Transverse	0.198	H&E
Plate 19a image g	E9.5	15	Transverse	0.249	H&E
Plate 19a image h	E9.5	15	Transverse	0.276	H&E
Plate 19a image i	E9.5	15	Transverse	0.296	H&E
Plate 19a image j	E9.5	15	Transverse	0.315	H&E
Plate 19a image k	E9.5	15	Transverse	0.339	H&E
Plate 19a image l	E9.5	15	Transverse	0.362	H&E
Plate 19b image a	E9.5	15	Transverse	0.389	H&E
Plate 19b image b	E9.5	15	Transverse	0.412	H&E
Plate 19b image c	E9.5	15	Transverse	0.436	H&E
Plate 19b image d	E9.5	15	Transverse	0.459	H&E
Plate 19b image e	E9.5	15	Transverse	0.475	H&E
Plate 19b image f	E9.5	15	Transverse	0.498	H&E
Plate 19b image g	E9.5	15	Transverse	0.525	H&E
Plate 19b image h	E9.5	15	Transverse	0.549	H&E
Plate 19b image i	E9.5	15	Transverse	0.572	H&E
Plate 19b image j	E9.5	15	Transverse	0.595	H&E
Plate 19b image k	E9.5	15	Transverse	0.619	H&E
Plate 19b image l	E9.5	15	Transverse	0.646	H&E
Plate 19c image a	E9.5	15	Transverse	0.673	H&E
Plate 19c image b	E9.5	15	Transverse	0.693	H&E
Plate 19c image c	E9.5	15	Transverse	0.712	H&E
Plate 19c image d	E9.5	15	Transverse	0.735	H&E
Plate 19c image e	E9.5	15	Transverse	0.763	H&E
Plate 19c image f	E9.5	15	Transverse	0.786	H&E
Plate 19c image g	E9.5	15	Transverse	0.813	H&E

Plate 19c image h	E9.5	15	Transverse	0.833	H&E
Plate 19c image i	E9.5	15	Transverse	0.856	H&E
Plate 19c image j	E9.5	15	Transverse	0.887	H&E
Plate 19c image k	E9.5	15	Transverse	0.922	H&E
Plate 19c image l	E9.5	15	Transverse	0.934	H&E
Plate 20a image a	E10-10.25	15	Transverse	0.016	H&E
Plate 20a image b	E10-10.25	15	Transverse	0.055	H&E
Plate 20a image c	E10-10.25	15	Transverse	0.091	H&E
Plate 20a image d	E10-10.25	15	Transverse	0.114	H&E
Plate 20a image e	E10-10.25	15	Transverse	0.146	H&E
Plate 20a image f	E10-10.25	15	Transverse	0.173	H&E
Plate 20a image g	E10-10.25	15	Transverse	0.197	H&E
Plate 20a image h	E10-10.25	15	Transverse	0.24	H&E
Plate 20a image i	E10-10.25	15	Transverse	0.268	H&E
Plate 20a image j	E10-10.25	15	Transverse	0.291	H&E
Plate 20a image k	E10-10.25	15	Transverse	0.311	H&E
Plate 20a image l	E10-10.25	15	Transverse	0.335	H&E
Plate 20b image a	E10-10.25	15	Transverse	0.362	H&E
Plate 20b image b	E10-10.25	15	Transverse	0.382	H&E
Plate 20b image c	E10-10.25	15	Transverse	0.402	H&E
Plate 20b image d	E10-10.25	15	Transverse	0.425	H&E
Plate 20b image e	E10-10.25	15	Transverse	0.449	H&E
Plate 20b image f	E10-10.25	15	Transverse	0.469	H&E
Plate 20b image g	E10-10.25	15	Transverse	0.492	H&E
Plate 20b image h	E10-10.25	15	Transverse	0.512	H&E
Plate 20b image i	E10-10.25	15	Transverse	0.531	H&E
Plate 20b image j	E10-10.25	15	Transverse	0.551	H&E
Plate 20b image k	E10-10.25	15	Transverse	0.575	H&E
Plate 20b image l	E10-10.25	15	Transverse	0.598	H&E
Plate 20c image a	E10-10.25	15	Transverse	0.614	H&E
Plate 20c image b	E10-10.25	15	Transverse	0.638	H&E
Plate 20c image c	E10-10.25	15	Transverse	0.661	H&E
Plate 20c image d	E10-10.25	15	Transverse	0.685	H&E
Plate 20c image e	E10-10.25	15	Transverse	0.705	H&E
Plate 20c image f	E10-10.25	15	Transverse	0.728	H&E
Plate 20c image g	E10-10.25	15	Transverse	0.756	H&E
Plate 20c image h	E10-10.25	15	Transverse	0.783	H&E
Plate 20c image i	E10-10.25	15	Transverse	0.823	H&E
Plate 20c image j	E10-10.25	15	Transverse	0.87	H&E
Plate 20c image k	E10-10.25	15	Transverse	0.913	H&E
Plate 20c image l	E10-10.25	15	Transverse	0.957	H&E

Plate 21a image a	E10-10.25	15	Sagittal	0.154	H&E
Plate 21a image b	E10-10.25	15	Sagittal	0.231	H&E
Plate 21a image c	E10-10.25	15	Sagittal	0.298	H&E
Plate 21a image d	E10-10.25	15	Sagittal	0.346	H&E
Plate 21b image a	E10-10.25	15	Sagittal	0.413	H&E
Plate 21b image b	E10-10.25	15	Sagittal	0.49	H&E
Plate 21b image c	E10-10.25	15	Sagittal	0.558	H&E
Plate 21b image d	E10-10.25	15	Sagittal	0.635	H&E
Plate 22a image a	E10.25-10.5	16	Sagittal	0.298	H&E
Plate 22a image b	E10.25-10.5	16	Sagittal	0.387	H&E
Plate 22a image c	E10.25-10.5	16	Sagittal	0.435	H&E
Plate 22a image d	E10.25-10.5	16	Sagittal	0.5	H&E
Plate 22b image a	E10.25-10.5	16	Sagittal	0.54	H&E
Plate 22b image b	E10.25-10.5	16	Sagittal	0.597	H&E
Plate 22b image c	E10.25-10.5	16	Sagittal	0.629	H&E
Plate 22b image d	E10.25-10.5	16	Sagittal	0.645	H&E
Plate 22c image a	E10.25-10.5	16	Sagittal	0.677	H&E
Plate 22c image b	E10.25-10.5	16	Sagittal	0.71	H&E
Plate 22c image c	E10.25-10.5	16	Sagittal	0.758	H&E
Plate 22c image d	E10.25-10.5	16	Sagittal	0.79	H&E
Plate 23a image a	E10.25-10.5	16-17	Transverse	0.019	H&E
Plate 23a image b	E10.25-10.5	16-17	Transverse	0.054	H&E
Plate 23a image c	E10.25-10.5	16-17	Transverse	0.073	H&E
Plate 23a image d	E10.25-10.5	16-17	Transverse	0.108	H&E
Plate 23a image e	E10.25-10.5	16-17	Transverse	0.124	H&E
Plate 23a image f	E10.25-10.5	16-17	Transverse	0.143	H&E
Plate 23a image g	E10.25-10.5	16-17	Transverse	0.158	H&E
Plate 23a image h	E10.25-10.5	16-17	Transverse	0.178	H&E
Plate 23a image i	E10.25-10.5	16-17	Transverse	0.197	H&E
Plate 23a image j	E10.25-10.5	16-17	Transverse	0.232	H&E
Plate 23a image k	E10.25-10.5	16-17	Transverse	0.247	H&E
Plate 23a image l	E10.25-10.5	16-17	Transverse	0.263	H&E
Plate 23a image m	E10.25-10.5	16-17	Transverse	0.282	H&E
Plate 23a image n	E10.25-10.5	16-17	Transverse	0.297	H&E
Plate 23a image o	E10.25-10.5	16-17	Transverse	0.317	H&E
Plate 23b image a	E10.25-10.5	16-17	Transverse	0.34	H&E
Plate 23b image b	E10.25-10.5	16-17	Transverse	0.355	H&E
Plate 23b image c	E10.25-10.5	16-17	Transverse	0.378	H&E
Plate 23b image d	E10.25-10.5	16-17	Transverse	0.394	H&E
Plate 23b image e	E10.25-10.5	16-17	Transverse	0.409	H&E
Plate 23b image f	E10.25-10.5	16-17	Transverse	0.425	H&E

Plate 23b image g	E10.25-10.5	16-17	Transverse	0.436	H&E
Plate 23b image h	E10.25-10.5	16-17	Transverse	0.448	H&E
Plate 23b image i	E10.25-10.5	16-17	Transverse	0.471	H&E
Plate 23b image j	E10.25-10.5	16-17	Transverse	0.486	H&E
Plate 23b image k	E10.25-10.5	16-17	Transverse	0.502	H&E
Plate 23b image l	E10.25-10.5	16-17	Transverse	0.521	H&E
Plate 23c image a	E10.25-10.5	16-17	Transverse	0.537	H&E
Plate 23c image b	E10.25-10.5	16-17	Transverse	0.552	H&E
Plate 23c image c	E10.25-10.5	16-17	Transverse	0.575	H&E
Plate 23c image d	E10.25-10.5	16-17	Transverse	0.595	H&E
Plate 23c image e	E10.25-10.5	16-17	Transverse	0.61	H&E
Plate 23c image f	E10.25-10.5	16-17	Transverse	0.629	H&E
Plate 23c image g	E10.25-10.5	16-17	Transverse	0.649	H&E
Plate 23c image h	E10.25-10.5	16-17	Transverse	0.668	H&E
Plate 23c image i	E10.25-10.5	16-17	Transverse	0.691	H&E
Plate 23c image j	E10.25-10.5	16-17	Transverse	0.707	H&E
Plate 23c image k	E10.25-10.5	16-17	Transverse	0.726	H&E
Plate 23c image l	E10.25-10.5	16-17	Transverse	0.737	H&E
Plate 23d image a	E10.25-10.5	16-17	Transverse	0.753	H&E
Plate 23d image b	E10.25-10.5	16-17	Transverse	0.772	H&E
Plate 23d image c	E10.25-10.5	16-17	Transverse	0.795	H&E
Plate 23d image d	E10.25-10.5	16-17	Transverse	0.807	H&E
Plate 23d image e	E10.25-10.5	16-17	Transverse	0.822	H&E
Plate 23d image f	E10.25-10.5	16-17	Transverse	0.842	H&E
Plate 23d image g	E10.25-10.5	16-17	Transverse	0.857	H&E
Plate 23d image h	E10.25-10.5	16-17	Transverse	0.876	H&E
Plate 23d image i	E10.25-10.5	16-17	Transverse	0.9	H&E
Plate 23d image j	E10.25-10.5	16-17	Transverse	0.915	H&E
Plate 23d image k	E10.25-10.5	16-17	Transverse	0.934	H&E
Plate 23d image l	E10.25-10.5	16-17	Transverse	0.954	H&E
Plate 23d image m	E10.25-10.5	16-17	Transverse	0.973	H&E
Plate 24a image a	E10.5-11	17-18	Transverse	0.063	H&E
Plate 24a image b	E10.5-11	17-18	Transverse	0.095	H&E
Plate 24a image c	E10.5-11	17-18	Transverse	0.115	H&E
Plate 24a image d	E10.5-11	17-18	Transverse	0.138	H&E
Plate 24a image e	E10.5-11	17-18	Transverse	0.158	H&E
Plate 24a image f	E10.5-11	17-18	Transverse	0.17	H&E
Plate 24a image g	E10.5-11	17-18	Transverse	0.186	H&E
Plate 24a image h	E10.5-11	17-18	Transverse	0.202	H&E
Plate 24a image i	E10.5-11	17-18	Transverse	0.229	H&E
Plate 24a image j	E10.5-11	17-18	Transverse	0.249	H&E

Plate 24a image k	E10.5-11	17-18	Transverse	0.261	H&E
Plate 24a image l	E10.5-11	17-18	Transverse	0.277	H&E
Plate 24a image m	E10.5-11	17-18	Transverse	0.292	H&E
Plate 24a image n	E10.5-11	17-18	Transverse	0.304	H&E
Plate 24a image o	E10.5-11	17-18	Transverse	0.312	H&E
Plate 24b image a	E10.5-11	17-18	Transverse	0.32	H&E
Plate 24b image b	E10.5-11	17-18	Transverse	0.332	H&E
Plate 24b image c	E10.5-11	17-18	Transverse	0.348	H&E
Plate 24b image d	E10.5-11	17-18	Transverse	0.364	H&E
Plate 24b image e	E10.5-11	17-18	Transverse	0.375	H&E
Plate 24b image f	E10.5-11	17-18	Transverse	0.391	H&E
Plate 24b image g	E10.5-11	17-18	Transverse	0.407	H&E
Plate 24b image h	E10.5-11	17-18	Transverse	0.423	H&E
Plate 24b image i	E10.5-11	17-18	Transverse	0.439	H&E
Plate 24b image j	E10.5-11	17-18	Transverse	0.451	H&E
Plate 24b image k	E10.5-11	17-18	Transverse	0.466	H&E
Plate 24b image l	E10.5-11	17-18	Transverse	0.482	H&E
Plate 24c image a	E10.5-11	17-18	Transverse	0.498	H&E
Plate 24c image b	E10.5-11	17-18	Transverse	0.514	H&E
Plate 24c image c	E10.5-11	17-18	Transverse	0.53	H&E
Plate 24c image d	E10.5-11	17-18	Transverse	0.545	H&E
Plate 24c image e	E10.5-11	17-18	Transverse	0.561	H&E
Plate 24c image f	E10.5-11	17-18	Transverse	0.573	H&E
Plate 24c image g	E10.5-11	17-18	Transverse	0.589	H&E
Plate 24c image h	E10.5-11	17-18	Transverse	0.613	H&E
Plate 24c image i	E10.5-11	17-18	Transverse	0.628	H&E
Plate 24c image j	E10.5-11	17-18	Transverse	0.644	H&E
Plate 24c image k	E10.5-11	17-18	Transverse	0.66	H&E
Plate 24c image l	E10.5-11	17-18	Transverse	0.676	H&E
Plate 24c image m	E10.5-11	17-18	Transverse	0.692	H&E
Plate 24c image n	E10.5-11	17-18	Transverse	0.704	H&E
Plate 24c image o	E10.5-11	17-18	Transverse	0.719	H&E
Plate 24d image a	E10.5-11	17-18	Transverse	0.727	H&E
Plate 24d image b	E10.5-11	17-18	Transverse	0.743	H&E
Plate 24d image c	E10.5-11	17-18	Transverse	0.759	H&E
Plate 24d image d	E10.5-11	17-18	Transverse	0.775	H&E
Plate 24d image e	E10.5-11	17-18	Transverse	0.791	H&E
Plate 24d image f	E10.5-11	17-18	Transverse	0.806	H&E
Plate 24d image g	E10.5-11	17-18	Transverse	0.822	H&E
Plate 24d image h	E10.5-11	17-18	Transverse	0.838	H&E
Plate 24d image i	E10.5-11	17-18	Transverse	0.85	H&E

Plate 24d image j	E10.5-11	17-18	Transverse	0.866	H&E
Plate 24d image k	E10.5-11	17-18	Transverse	0.881	H&E
Plate 24d image l	E10.5-11	17-18	Transverse	0.893	H&E
Plate 24d image m	E10.5-11	17-18	Transverse	0.909	H&E
Plate 24d image n	E10.5-11	17-18	Transverse	0.929	H&E
Plate 24d image o	E10.5-11	17-18	Transverse	0.953	H&E
Plate 25a image a	E11-11.5	18	Sagittal	0.817	H&E
Plate 25a image b	E11-11.5	18	Sagittal	0.785	H&E
Plate 25a image c	E11-11.5	18	Sagittal	0.742	H&E
Plate 25a image d	E11-11.5	18	Sagittal	0.688	H&E
Plate 25b image a	E11-11.5	18	Sagittal	0.634	H&E
Plate 25b image b	E11-11.5	18	Sagittal	0.581	H&E
Plate 25b image c	E11-11.5	18	Sagittal	0.548	H&E
Plate 25b image d	E11-11.5	18	Sagittal	0.505	H&E
Plate 25c image a	E11-11.5	18	Sagittal	0.43	H&E
Plate 25c image b	E11-11.5	18	Sagittal	0.387	H&E
Plate 25c image c	E11-11.5	18	Sagittal	0.29	H&E
Plate 25c image d	E11-11.5	18	Sagittal	0.226	H&E
Plate 26a image a	E11.5-12	19	Transverse	0.026	H&E
Plate 26a image b	E11.5-12	19	Transverse	0.068	H&E
Plate 26a image c	E11.5-12	19	Transverse	0.098	H&E
Plate 26a image d	E11.5-12	19	Transverse	0.143	H&E
Plate 26a image e	E11.5-12	19	Transverse	0.177	H&E
Plate 26a image f	E11.5-12	19	Transverse	0.204	H&E
Plate 26a image g	E11.5-12	19	Transverse	0.219	H&E
Plate 26a image h	E11.5-12	19	Transverse	0.238	H&E
Plate 26a image i	E11.5-12	19	Transverse	0.249	H&E
Plate 26a image j	E11.5-12	19	Transverse	0.272	H&E
Plate 26b image a	E11.5-12	19	Transverse	0.287	H&E
Plate 26b image b	E11.5-12	19	Transverse	0.306	H&E
Plate 26b image c	E11.5-12	19	Transverse	0.321	H&E
Plate 26b image d	E11.5-12	19	Transverse	0.336	H&E
Plate 26b image e	E11.5-12	19	Transverse	0.358	H&E
Plate 26b image f	E11.5-12	19	Transverse	0.381	H&E
Plate 26b image g	E11.5-12	19	Transverse	0.404	H&E
Plate 26b image h	E11.5-12	19	Transverse	0.426	H&E
Plate 26b image i	E11.5-12	19	Transverse	0.457	H&E
Plate 26c image a	E11.5-12	19	Transverse	0.475	H&E
Plate 26c image b	E11.5-12	19	Transverse	0.494	H&E
Plate 26c image c	E11.5-12	19	Transverse	0.509	H&E
Plate 26c image d	E11.5-12	19	Transverse	0.525	H&E

Plate 26c image e	E11.5-12	19	Transverse	0.54	H&E
Plate 26c image f	E11.5-12	19	Transverse	0.558	H&E
Plate 26c image g	E11.5-12	19	Transverse	0.574	H&E
Plate 26c image h	E11.5-12	19	Transverse	0.589	H&E
Plate 26c image i	E11.5-12	19	Transverse	0.6	H&E
Plate 26d image a	E11.5-12	19	Transverse	0.619	H&E
Plate 26d image b	E11.5-12	19	Transverse	0.63	H&E
Plate 26d image c	E11.5-12	19	Transverse	0.645	H&E
Plate 26d image d	E11.5-12	19	Transverse	0.66	H&E
Plate 26d image e	E11.5-12	19	Transverse	0.679	H&E
Plate 26d image f	E11.5-12	19	Transverse	0.694	H&E
Plate 26d image g	E11.5-12	19	Transverse	0.709	H&E
Plate 26d image h	E11.5-12	19	Transverse	0.725	H&E
Plate 26d image i	E11.5-12	19	Transverse	0.74	H&E
Plate 26e image a	E11.5-12	19	Transverse	0.758	H&E
Plate 26e image b	E11.5-12	19	Transverse	0.785	H&E
Plate 26e image c	E11.5-12	19	Transverse	0.804	H&E
Plate 26e image d	E11.5-12	19	Transverse	0.826	H&E
Plate 26e image e	E11.5-12	19	Transverse	0.853	H&E
Plate 26e image f	E11.5-12	19	Transverse	0.875	H&E
Plate 26e image g	E11.5-12	19	Transverse	0.894	H&E
Plate 26e image h	E11.5-12	19	Transverse	0.925	H&E
Plate 26e image i	E11.5-12	19	Transverse	0.958	H&E
Plate 26e image j	E11.5-12	19	Transverse	0.985	H&E
Plate 27a image a	E12-12.5	20	Transverse	0.146	H&E
Plate 27a image b	E12-12.5	20	Transverse	0.174	H&E
Plate 27a image c	E12-12.5	20	Transverse	0.217	H&E
Plate 27a image d	E12-12.5	20	Transverse	0.257	H&E
Plate 27a image e	E12-12.5	20	Transverse	0.281	H&E
Plate 27a image f	E12-12.5	20	Transverse	0.312	H&E
Plate 27a image g	E12-12.5	20	Transverse	0.332	H&E
Plate 27a image h	E12-12.5	20	Transverse	0.348	H&E
Plate 27a image i	E12-12.5	20	Transverse	0.368	H&E
Plate 27a image j	E12-12.5	20	Transverse	0.391	H&E
Plate 27b image a	E12-12.5	20	Transverse	0.427	H&E
Plate 27b image b	E12-12.5	20	Transverse	0.443	H&E
Plate 27b image c	E12-12.5	20	Transverse	0.462	H&E
Plate 27b image d	E12-12.5	20	Transverse	0.478	H&E
Plate 27b image e	E12-12.5	20	Transverse	0.502	H&E
Plate 27b image f	E12-12.5	20	Transverse	0.518	H&E
Plate 27b image g	E12-12.5	20	Transverse	0.542	H&E

Plate 27b image h	E12-12.5	20	Transverse	0.569	H&E
Plate 27c image a	E12-12.5	20	Transverse	0.589	H&E
Plate 27c image b	E12-12.5	20	Transverse	0.609	H&E
Plate 27c image c	E12-12.5	20	Transverse	0.628	H&E
Plate 27c image d	E12-12.5	20	Transverse	0.648	H&E
Plate 27c image e	E12-12.5	20	Transverse	0.68	H&E
Plate 27c image f	E12-12.5	20	Transverse	0.704	H&E
Plate 27d image a	E12-12.5	20	Transverse	0.731	H&E
Plate 27d image b	E12-12.5	20	Transverse	0.751	H&E
Plate 27d image c	E12-12.5	20	Transverse	0.767	H&E
Plate 27d image d	E12-12.5	20	Transverse	0.791	H&E
Plate 27d image e	E12-12.5	20	Transverse	0.81	H&E
Plate 27d image f	E12-12.5	20	Transverse	0.83	H&E
Plate 27d image g	E12-12.5	20	Transverse	0.854	H&E
Plate 27d image h	E12-12.5	20	Transverse	0.897	H&E
Plate 27d image i	E12-12.5	20	Transverse	0.921	H&E
Plate 27d image j	E12-12.5	20	Transverse	0.949	H&E
Plate 28a image a	E12.5-13	21	Transverse	0.125	H&E
Plate 28a image b	E12.5-13	21	Transverse	0.166	H&E
Plate 28a image c	E12.5-13	21	Transverse	0.185	H&E
Plate 28a image d	E12.5-13	21	Transverse	0.211	H&E
Plate 28a image e	E12.5-13	21	Transverse	0.23	H&E
Plate 28a image f	E12.5-13	21	Transverse	0.249	H&E
Plate 28a image g	E12.5-13	21	Transverse	0.268	H&E
Plate 28a image h	E12.5-13	21	Transverse	0.287	H&E
Plate 28a image i	E12.5-13	21	Transverse	0.306	H&E
Plate 28b image a	E12.5-13	21	Transverse	0.325	H&E
Plate 28b image b	E12.5-13	21	Transverse	0.34	H&E
Plate 28b image c	E12.5-13	21	Transverse	0.351	H&E
Plate 28b image d	E12.5-13	21	Transverse	0.366	H&E
Plate 28b image e	E12.5-13	21	Transverse	0.385	H&E
Plate 28b image f	E12.5-13	21	Transverse	0.4	H&E
Plate 28b image g	E12.5-13	21	Transverse	0.419	H&E
Plate 28b image h	E12.5-13	21	Transverse	0.438	H&E
Plate 28b image i	E12.5-13	21	Transverse	0.464	H&E
Plate 28c image a	E12.5-13	21	Transverse	0.479	H&E
Plate 28c image b	E12.5-13	21	Transverse	0.498	H&E
Plate 28c image c	E12.5-13	21	Transverse	0.517	H&E
Plate 28c image d	E12.5-13	21	Transverse	0.536	H&E
Plate 28c image e	E12.5-13	21	Transverse	0.547	H&E
Plate 28c image f	E12.5-13	21	Transverse	0.558	H&E

Plate 28c image g	E12.5-13	21	Transverse	0.577	H&E
Plate 28d image a	E12.5-13	21	Transverse	0.592	H&E
Plate 28d image b	E12.5-13	21	Transverse	0.611	H&E
Plate 28d image c	E12.5-13	21	Transverse	0.626	H&E
Plate 28d image d	E12.5-13	21	Transverse	0.649	H&E
Plate 28d image e	E12.5-13	21	Transverse	0.668	H&E
Plate 28d image f	E12.5-13	21	Transverse	0.702	H&E
Plate 28d image g	E12.5-13	21	Transverse	0.721	H&E
Plate 28d image h	E12.5-13	21	Transverse	0.74	H&E
Plate 28d image i	E12.5-13	21	Transverse	0.758	H&E
Plate 28e image a	E12.5-13	21	Transverse	0.777	H&E
Plate 28e image b	E12.5-13	21	Transverse	0.792	H&E
Plate 28e image c	E12.5-13	21	Transverse	0.815	H&E
Plate 28e image d	E12.5-13	21	Transverse	0.826	H&E
Plate 28e image e	E12.5-13	21	Transverse	0.842	H&E
Plate 28e image f	E12.5-13	21	Transverse	0.853	H&E
Plate 28e image g	E12.5-13	21	Transverse	0.879	H&E
Plate 28e image h	E12.5-13	21	Transverse	0.894	H&E
Plate 28e image i	E12.5-13	21	Transverse	0.921	H&E
Plate 28e image j	E12.5-13	21	Transverse	0.943	H&E
Plate 29a image a	E12.5-13	21	Sagittal	0.447	H&E
Plate 29a image b	E12.5-13	21	Sagittal	0.545	H&E
Plate 29b image a	E12.5-13	21	Sagittal	0.602	H&E
Plate 29b image b	E12.5-13	21	Sagittal	0.642	H&E
Plate 30a image a	E13.5	22	Transverse	0.127	H&E
Plate 30a image b	E13.5	22	Transverse	0.15	H&E
Plate 30a image c	E13.5	22	Transverse	0.185	H&E
Plate 30a image d	E13.5	22	Transverse	0.204	H&E
Plate 30b image a	E13.5	22	Transverse	0.227	H&E
Plate 30b image b	E13.5	22	Transverse	0.238	H&E
Plate 30b image c	E13.5	22	Transverse	0.254	H&E
Plate 30b image d	E13.5	22	Transverse	0.273	H&E
Plate 30c image a	E13.5	22	Transverse	0.285	H&E
Plate 30c image b	E13.5	22	Transverse	0.3	H&E
Plate 30c image c	E13.5	22	Transverse	0.312	H&E
Plate 30c image d	E13.5	22	Transverse	0.323	H&E
Plate 30d image a	E13.5	22	Transverse	0.335	H&E
Plate 30d image b	E13.5	22	Transverse	0.354	H&E
Plate 30d image c	E13.5	22	Transverse	0.365	H&E
Plate 30d image d	E13.5	22	Transverse	0.381	H&E
Plate 30e image a	E13.5	22	Transverse	0.392	H&E

Plate 30e image b	E13.5	22	Transverse	0.408	H&E
Plate 30e image c	E13.5	22	Transverse	0.419	H&E
Plate 30e image d	E13.5	22	Transverse	0.435	H&E
Plate 30f image a	E13.5	22	Transverse	0.45	H&E
Plate 30f image b	E13.5	22	Transverse	0.465	H&E
Plate 30f image c	E13.5	22	Transverse	0.481	H&E
Plate 30f image d	E13.5	22	Transverse	0.492	H&E
Plate 30g image a	E13.5	22	Transverse	0.508	H&E
Plate 30g image b	E13.5	22	Transverse	0.523	H&E
Plate 30g image c	E13.5	22	Transverse	0.538	H&E
Plate 30g image d	E13.5	22	Transverse	0.55	H&E
Plate 30h image a	E13.5	22	Transverse	0.562	H&E
Plate 30h image b	E13.5	22	Transverse	0.573	H&E
Plate 30h image c	E13.5	22	Transverse	0.592	H&E
Plate 30h image d	E13.5	22	Transverse	0.608	H&E
Plate 30i image a	E13.5	22	Transverse	0.627	H&E
Plate 30i image b	E13.5	22	Transverse	0.642	H&E
Plate 30i image c	E13.5	22	Transverse	0.662	H&E
Plate 30i image d	E13.5	22	Transverse	0.685	H&E
Plate 30j image a	E13.5	22	Transverse	0.712	H&E
Plate 30j image b	E13.5	22	Transverse	0.735	H&E
Plate 30j image c	E13.5	22	Transverse	0.75	H&E
Plate 30j image d	E13.5	22	Transverse	0.765	H&E
Plate 30k image a	E13.5	22	Transverse	0.777	H&E
Plate 30k image b	E13.5	22	Transverse	0.788	H&E
Plate 30k image c	E13.5	22	Transverse	0.8	H&E
Plate 30k image d	E13.5	22	Transverse	0.812	H&E
Plate 30l image a	E13.5	22	Transverse	0.831	H&E
Plate 30l image b	E13.5	22	Transverse	0.846	H&E
Plate 30l image c	E13.5	22	Transverse	0.862	H&E
Plate 30l image d	E13.5	22	Transverse	0.881	H&E
Plate 31a image a	E13.5	22	Sagittal	0.593	H&E
Plate 31a image b	E13.5	22	Sagittal	0.512	H&E
Plate 31b image a	E13.5	22	Sagittal	0.455	H&E
Plate 31b image b	E13.5	22	Sagittal	0.39	H&E
Plate 32a image a	E14.5	22-23	Transverse	0.064	H&E
Plate 32a image b	E14.5	22-23	Transverse	0.094	H&E
Plate 32a image c	E14.5	22-23	Transverse	0.124	H&E
Plate 32a image d	E14.5	22-23	Transverse	0.15	H&E
Plate 32b image a	E14.5	22-23	Transverse	0.173	H&E
Plate 32b image b	E14.5	22-23	Transverse	0.195	H&E

Plate 32b image c	E14.5	22-23	Transverse	0.218	H&E
Plate 32b image d	E14.5	22-23	Transverse	0.241	H&E
Plate 32c image a	E14.5	22-23	Transverse	0.256	H&E
Plate 32c image b	E14.5	22-23	Transverse	0.271	H&E
Plate 32c image c	E14.5	22-23	Transverse	0.286	H&E
Plate 32c image d	E14.5	22-23	Transverse	0.301	H&E
Plate 32d image a	E14.5	22-23	Transverse	0.32	H&E
Plate 32d image b	E14.5	22-23	Transverse	0.335	H&E
Plate 32d image c	E14.5	22-23	Transverse	0.346	H&E
Plate 32d image d	E14.5	22-23	Transverse	0.361	H&E
Plate 32e image a	E14.5	22-23	Transverse	0.376	H&E
Plate 32e image b	E14.5	22-23	Transverse	0.391	H&E
Plate 32e image c	E14.5	22-23	Transverse	0.41	H&E
Plate 32e image d	E14.5	22-23	Transverse	0.432	H&E
Plate 32f image a	E14.5	22-23	Transverse	0.455	H&E
Plate 32f image b	E14.5	22-23	Transverse	0.466	H&E
Plate 32f image c	E14.5	22-23	Transverse	0.481	H&E
Plate 32f image d	E14.5	22-23	Transverse	0.496	H&E
Plate 32g image a	E14.5	22-23	Transverse	0.519	H&E
Plate 32g image b	E14.5	22-23	Transverse	0.534	H&E
Plate 32g image c	E14.5	22-23	Transverse	0.549	H&E
Plate 32g image d	E14.5	22-23	Transverse	0.56	H&E
Plate 32h image a	E14.5	22-23	Transverse	0.575	H&E
Plate 32h image b	E14.5	22-23	Transverse	0.594	H&E
Plate 32h image c	E14.5	22-23	Transverse	0.632	H&E
Plate 32h image d	E14.5	22-23	Transverse	0.658	H&E
Plate 32i image a	E14.5	22-23	Transverse	0.688	H&E
Plate 32i image b	E14.5	22-23	Transverse	0.714	H&E
Plate 32i image c	E14.5	22-23	Transverse	0.748	H&E
Plate 32i image d	E14.5	22-23	Transverse	0.771	H&E
Plate 32j image a	E14.5	22-23	Transverse	0.789	H&E
Plate 32j image b	E14.5	22-23	Transverse	0.805	H&E
Plate 32j image c	E14.5	22-23	Transverse	0.82	H&E
Plate 32j image d	E14.5	22-23	Transverse	0.838	H&E
Plate 32k image a	E14.5	22-23	Transverse	0.85	H&E
Plate 32k image b	E14.5	22-23	Transverse	0.861	H&E
Plate 32k image c	E14.5	22-23	Transverse	0.883	H&E
Plate 32k image d	E14.5	22-23	Transverse	0.898	H&E
Plate 32k image e	E14.5	22-23	Transverse	0.936	H&E
Plate 33a image a	E14.5	22-23	Sagittal	0.393	H&E
Plate 33a image b	E14.5	22-23	Sagittal	0.446	H&E

Plate 33b image a	E14.5	22-23	Sagittal	0.473	H&E
Plate 33b image b	E14.5	22-23	Sagittal	0.509	H&E
Plate 34a image a	E14.5	22-23	Coronal	0.12	H&E
Plate 34a image b	E14.5	22-23	Coronal	0.174	H&E
Plate 34a image c	E14.5	22-23	Coronal	0.257	H&E
Plate 34a image d	E14.5	22-23	Coronal	0.317	H&E
Plate 34a image e	E14.5	22-23	Coronal	0.371	H&E
Plate 34a image f	E14.5	22-23	Coronal	0.425	H&E
Plate 34a image g	E14.5	22-23	Coronal	0.485	H&E
Plate 34a image h	E14.5	22-23	Coronal	0.551	H&E
Plate 34b image a	E14.5	22-23	Coronal	0.593	H&E
Plate 34b image b	E14.5	22-23	Coronal	0.665	H&E
Plate 34b image c	E14.5	22-23	Coronal	0.707	H&E
Plate 34b image d	E14.5	22-23	Coronal	0.731	H&E
Plate 34b image e	E14.5	22-23	Coronal	0.766	H&E
Plate 34b image f	E14.5	22-23	Coronal	0.844	H&E
Plate 34b image g	E14.5	22-23	Coronal	0.886	H&E
Plate 34b image h	E14.5	22-23	Coronal	0.94	H&E
Plate 35a image a	E15.5	24	Transverse	0.069	H&E
Plate 35a image b	E15.5	24	Transverse	0.096	H&E
Plate 35a image c	E15.5	24	Transverse	0.119	H&E
Plate 35a image d	E15.5	24	Transverse	0.138	H&E
Plate 35a image e	E15.5	24	Transverse	0.158	H&E
Plate 35b image a	E15.5	24	Transverse	0.181	H&E
Plate 35b image b	E15.5	24	Transverse	0.196	H&E
Plate 35b image c	E15.5	24	Transverse	0.215	H&E
Plate 35b image d	E15.5	24	Transverse	0.227	H&E
Plate 35c image a	E15.5	24	Transverse	0.242	H&E
Plate 35c image b	E15.5	24	Transverse	0.258	H&E
Plate 35c image c	E15.5	24	Transverse	0.269	H&E
Plate 35c image d	E15.5	24	Transverse	0.285	H&E
Plate 35d image a	E15.5	24	Transverse	0.3	H&E
Plate 35d image b	E15.5	24	Transverse	0.312	H&E
Plate 35d image c	E15.5	24	Transverse	0.327	H&E
Plate 35d image d	E15.5	24	Transverse	0.342	H&E
Plate 35e image a	E15.5	24	Transverse	0.354	H&E
Plate 35e image b	E15.5	24	Transverse	0.369	H&E
Plate 35e image c	E15.5	24	Transverse	0.392	H&E
Plate 35e image d	E15.5	24	Transverse	0.404	H&E
Plate 35f image a	E15.5	24	Transverse	0.415	H&E
Plate 35f image b	E15.5	24	Transverse	0.431	H&E

Plate 35f image c	E15.5	24	Transverse	0.446	H&E
Plate 35f image d	E15.5	24	Transverse	0.462	H&E
Plate 35g image a	E15.5	24	Transverse	0.477	H&E
Plate 35g image b	E15.5	24	Transverse	0.496	H&E
Plate 35g image c	E15.5	24	Transverse	0.512	H&E
Plate 35g image d	E15.5	24	Transverse	0.527	H&E
Plate 35h image a	E15.5	24	Transverse	0.542	H&E
Plate 35h image b	E15.5	24	Transverse	0.558	H&E
Plate 35h image c	E15.5	24	Transverse	0.573	H&E
Plate 35h image d	E15.5	24	Transverse	0.596	H&E
Plate 35i image a	E15.5	24	Transverse	0.623	H&E
Plate 35i image b	E15.5	24	Transverse	0.65	H&E
Plate 35i image c	E15.5	24	Transverse	0.681	H&E
Plate 35i image d	E15.5	24	Transverse	0.708	H&E
Plate 35j image a	E15.5	24	Transverse	0.742	H&E
Plate 35j image b	E15.5	24	Transverse	0.758	H&E
Plate 35j image c	E15.5	24	Transverse	0.777	H&E
Plate 35j image d	E15.5	24	Transverse	0.8	H&E
Plate 35k image a	E15.5	24	Transverse	0.815	H&E
Plate 35k image b	E15.5	24	Transverse	0.827	H&E
Plate 35k image c	E15.5	24	Transverse	0.842	H&E
Plate 35k image d	E15.5	24	Transverse	0.858	H&E
Plate 35l image a	E15.5	24	Transverse	0.873	H&E
Plate 35l image b	E15.5	24	Transverse	0.892	H&E
Plate 35l image c	E15.5	24	Transverse	0.912	H&E
Plate 35l image d	E15.5	24	Transverse	0.927	H&E
Plate 35l image e	E15.5	24	Transverse	0.946	H&E
Plate 36a image a	E15.5	24	Sagittal	0.483	H&E
Plate 36a image b	E15.5	24	Sagittal	0.533	H&E
Plate 36b image a	E15.5	24	Sagittal	0.592	H&E
Plate 36b image b	E15.5	24	Sagittal	0.625	H&E
Plate 36c image a	E15.5	24	Sagittal	0.65	H&E
Plate 36c image b	E15.5	24	Sagittal	0.7	H&E
Plate 36d image a	E15.5	24	Sagittal	0.717	H&E
Plate 36d image b	E15.5	24	Sagittal	0.75	H&E
Plate 36e image a	E15.5	24	Sagittal	0.783	H&E
Plate 36e image b	E15.5	24	Sagittal	0.808	H&E
Plate 37a image a	E16.5	25	Transverse	0.059	H&E
Plate 37a image b	E16.5	25	Transverse	0.085	H&E
Plate 37a image c	E16.5	25	Transverse	0.115	H&E
Plate 37a image d	E16.5	25	Transverse	0.144	H&E

Plate 37a image e	E16.5	25	Transverse	0.167	H&E
Plate 37b image a	E16.5	25	Transverse	0.2	H&E
Plate 37b image b	E16.5	25	Transverse	0.219	H&E
Plate 37b image c	E16.5	25	Transverse	0.237	H&E
Plate 37b image d	E16.5	25	Transverse	0.256	H&E
Plate 37c image a	E16.5	25	Transverse	0.281	H&E
Plate 37c image b	E16.5	25	Transverse	0.304	H&E
Plate 37c image c	E16.5	25	Transverse	0.319	H&E
Plate 37c image d	E16.5	25	Transverse	0.337	H&E
Plate 37d image a	E16.5	25	Transverse	0.352	H&E
Plate 37d image b	E16.5	25	Transverse	0.37	H&E
Plate 37d image c	E16.5	25	Transverse	0.385	H&E
Plate 37d image d	E16.5	25	Transverse	0.407	H&E
Plate 37e image a	E16.5	25	Transverse	0.433	H&E
Plate 37e image b	E16.5	25	Transverse	0.448	H&E
Plate 37e image c	E16.5	25	Transverse	0.456	H&E
Plate 37e image d	E16.5	25	Transverse	0.47	H&E
Plate 37f image a	E16.5	25	Transverse	0.485	H&E
Plate 37f image b	E16.5	25	Transverse	0.496	H&E
Plate 37f image c	E16.5	25	Transverse	0.511	H&E
Plate 37f image d	E16.5	25	Transverse	0.533	H&E
Plate 37g image a	E16.5	25	Transverse	0.559	H&E
Plate 37g image b	E16.5	25	Transverse	0.57	H&E
Plate 37g image c	E16.5	25	Transverse	0.581	H&E
Plate 37g image d	E16.5	25	Transverse	0.596	H&E
Plate 37h image a	E16.5	25	Transverse	0.607	H&E
Plate 37h image b	E16.5	25	Transverse	0.619	H&E
Plate 37h image c	E16.5	25	Transverse	0.637	H&E
Plate 37h image d	E16.5	25	Transverse	0.656	H&E
Plate 37i image a	E16.5	25	Transverse	0.674	H&E
Plate 37i image b	E16.5	25	Transverse	0.696	H&E
Plate 37i image c	E16.5	25	Transverse	0.719	H&E
Plate 37i image d	E16.5	25	Transverse	0.733	H&E
Plate 37j image a	E16.5	25	Transverse	0.759	H&E
Plate 37j image b	E16.5	25	Transverse	0.778	H&E
Plate 37j image c	E16.5	25	Transverse	0.793	H&E
Plate 37j image d	E16.5	25	Transverse	0.815	H&E
Plate 37k image a	E16.5	25	Transverse	0.837	H&E
Plate 37k image b	E16.5	25	Transverse	0.844	H&E
Plate 37k image c	E16.5	25	Transverse	0.856	H&E
Plate 37k image d	E16.5	25	Transverse	0.867	H&E

Plate 37l image a	E16.5	25	Transverse	0.878	H&E
Plate 37l image b	E16.5	25	Transverse	0.896	H&E
Plate 37l image c	E16.5	25	Transverse	0.919	H&E
Plate 37l image d	E16.5	25	Transverse	0.933	H&E
Plate 37l image e	E16.5	25	Transverse	0.952	H&E
Plate 38a image a	E16.5	25	Sagittal	0.368	H&E
Plate 38a image b	E16.5	25	Sagittal	0.43	H&E
Plate 38b image a	E16.5	25	Sagittal	0.465	H&E
Plate 38b image b	E16.5	25	Sagittal	0.518	H&E
Plate 38c image a	E16.5	25	Sagittal	0.553	H&E
Plate 38c image b	E16.5	25	Sagittal	0.632	H&E
Plate 39a image a	E16.5	25	Coronal	0.14	H&E
Plate 39a image b	E16.5	25	Coronal	0.207	H&E
Plate 39a image c	E16.5	25	Coronal	0.305	H&E
Plate 39a image d	E16.5	25	Coronal	0.335	H&E
Plate 39a image e	E16.5	25	Coronal	0.39	H&E
Plate 39a image f	E16.5	25	Coronal	0.457	H&E
Plate 39b image a	E16.5	25	Coronal	0.494	H&E
Plate 39b image b	E16.5	25	Coronal	0.53	H&E
Plate 39b image c	E16.5	25	Coronal	0.561	H&E
Plate 39b image d	E16.5	25	Coronal	0.591	H&E
Plate 39c image a	E16.5	25	Coronal	0.616	H&E
Plate 39c image b	E16.5	25	Coronal	0.646	H&E
Plate 39c image c	E16.5	25	Coronal	0.677	H&E
Plate 39c image d	E16.5	25	Coronal	0.713	H&E
Plate 39d image a	E16.5	25	Coronal	0.762	H&E
Plate 39d image b	E16.5	25	Coronal	0.799	H&E
Plate 39d image c	E16.5	25	Coronal	0.854	H&E
Plate 39d image d	E16.5	25	Coronal	0.921	H&E
Plate 40a image a	E17.5	26	Transverse	0.067	H&E
Plate 40a image b	E17.5	26	Transverse	0.09	H&E
Plate 40a image c	E17.5	26	Transverse	0.105	H&E
Plate 40a image d	E17.5	26	Transverse	0.124	H&E
Plate 40a image e	E17.5	26	Transverse	0.146	H&E
Plate 40b image a	E17.5	26	Transverse	0.161	H&E
Plate 40b image b	E17.5	26	Transverse	0.172	H&E
Plate 40b image c	E17.5	26	Transverse	0.195	H&E
Plate 40b image d	E17.5	26	Transverse	0.21	H&E
Plate 40c image a	E17.5	26	Transverse	0.225	H&E
Plate 40c image b	E17.5	26	Transverse	0.24	H&E
Plate 40c image c	E17.5	26	Transverse	0.255	H&E

Plate 40c image d	E17.5	26	Transverse	0.27	H&E
Plate 40d image a	E17.5	26	Transverse	0.288	H&E
Plate 40d image b	E17.5	26	Transverse	0.303	H&E
Plate 40d image c	E17.5	26	Transverse	0.322	H&E
Plate 40d image d	E17.5	26	Transverse	0.345	H&E
Plate 40e image a	E17.5	26	Transverse	0.356	H&E
Plate 40e image b	E17.5	26	Transverse	0.371	H&E
Plate 40e image c	E17.5	26	Transverse	0.39	H&E
Plate 40e image d	E17.5	26	Transverse	0.404	H&E
Plate 40f image a	E17.5	26	Transverse	0.423	H&E
Plate 40f image b	E17.5	26	Transverse	0.438	H&E
Plate 40f image c	E17.5	26	Transverse	0.457	H&E
Plate 40f image d	E17.5	26	Transverse	0.476	H&E
Plate 40g image a	E17.5	26	Transverse	0.498	H&E
Plate 40g image b	E17.5	26	Transverse	0.521	H&E
Plate 40g image c	E17.5	26	Transverse	0.543	H&E
Plate 40g image d	E17.5	26	Transverse	0.558	H&E
Plate 40g image e	E17.5	26	Transverse	0.577	H&E
Plate 40g image f	E17.5	26	Transverse	0.588	H&E
Plate 40h image a	E17.5	26	Transverse	0.603	H&E
Plate 40h image b	E17.5	26	Transverse	0.622	H&E
Plate 40h image c	E17.5	26	Transverse	0.637	H&E
Plate 40h image d	E17.5	26	Transverse	0.652	H&E
Plate 40i image a	E17.5	26	Transverse	0.67	H&E
Plate 40i image b	E17.5	26	Transverse	0.685	H&E
Plate 40i image c	E17.5	26	Transverse	0.7	H&E
Plate 40i image d	E17.5	26	Transverse	0.715	H&E
Plate 40j image a	E17.5	26	Transverse	0.73	H&E
Plate 40j image b	E17.5	26	Transverse	0.745	H&E
Plate 40j image c	E17.5	26	Transverse	0.76	H&E
Plate 40j image d	E17.5	26	Transverse	0.779	H&E
Plate 40k image a	E17.5	26	Transverse	0.801	H&E
Plate 40k image b	E17.5	26	Transverse	0.82	H&E
Plate 40k image c	E17.5	26	Transverse	0.839	H&E
Plate 40k image d	E17.5	26	Transverse	0.85	H&E
Plate 40l image a	E17.5	26	Transverse	0.869	H&E
Plate 40l image b	E17.5	26	Transverse	0.888	H&E
Plate 40l image c	E17.5	26	Transverse	0.903	H&E
Plate 40l image d	E17.5	26	Transverse	0.921	H&E
Plate 40l image e	E17.5	26	Transverse	0.948	H&E
Plate 41 image 41	E17.5	26	Sagittal	0.496	H&E

evelopment: Table 3

DOI
http://dx.doi.org/10.7488/ds/393
http://dx.doi.org/10.7488/ds/394
http://dx.doi.org/10.7488/ds/395
http://dx.doi.org/10.7488/ds/396
http://dx.doi.org/10.7488/ds/397
http://dx.doi.org/10.7488/ds/398
http://dx.doi.org/10.7488/ds/399
http://dx.doi.org/10.7488/ds/400
http://dx.doi.org/10.7488/ds/401
http://dx.doi.org/10.7488/ds/402
http://dx.doi.org/10.7488/ds/403
http://dx.doi.org/10.7488/ds/404
http://dx.doi.org/10.7488/ds/405
http://dx.doi.org/10.7488/ds/406
http://dx.doi.org/10.7488/ds/407
http://dx.doi.org/10.7488/ds/408
http://dx.doi.org/10.7488/ds/409
http://dx.doi.org/10.7488/ds/410
http://dx.doi.org/10.7488/ds/411
http://dx.doi.org/10.7488/ds/412
http://dx.doi.org/10.7488/ds/413
http://dx.doi.org/10.7488/ds/414
http://dx.doi.org/10.7488/ds/415
http://dx.doi.org/10.7488/ds/416
http://dx.doi.org/10.7488/ds/417
http://dx.doi.org/10.7488/ds/418
http://dx.doi.org/10.7488/ds/419
http://dx.doi.org/10.7488/ds/420
http://dx.doi.org/10.7488/ds/421
http://dx.doi.org/10.7488/ds/422
http://dx.doi.org/10.7488/ds/423
http://dx.doi.org/10.7488/ds/424
http://dx.doi.org/10.7488/ds/425
http://dx.doi.org/10.7488/ds/426
http://dx.doi.org/10.7488/ds/427
http://dx.doi.org/10.7488/ds/428
http://dx.doi.org/10.7488/ds/429

http://dx.doi.org/10.7488/ds/430
http://dx.doi.org/10.7488/ds/431
http://dx.doi.org/10.7488/ds/432
http://dx.doi.org/10.7488/ds/433
http://dx.doi.org/10.7488/ds/434
http://dx.doi.org/10.7488/ds/435
http://dx.doi.org/10.7488/ds/436
http://dx.doi.org/10.7488/ds/437
http://dx.doi.org/10.7488/ds/438
http://dx.doi.org/10.7488/ds/439
http://dx.doi.org/10.7488/ds/440
http://dx.doi.org/10.7488/ds/441
http://dx.doi.org/10.7488/ds/442
http://dx.doi.org/10.7488/ds/443
http://dx.doi.org/10.7488/ds/444
http://dx.doi.org/10.7488/ds/445
http://dx.doi.org/10.7488/ds/446
http://dx.doi.org/10.7488/ds/447
http://dx.doi.org/10.7488/ds/448
http://dx.doi.org/10.7488/ds/449
http://dx.doi.org/10.7488/ds/450
http://dx.doi.org/10.7488/ds/451
http://dx.doi.org/10.7488/ds/452
http://dx.doi.org/10.7488/ds/453
http://dx.doi.org/10.7488/ds/454
http://dx.doi.org/10.7488/ds/455
http://dx.doi.org/10.7488/ds/456
http://dx.doi.org/10.7488/ds/457
http://dx.doi.org/10.7488/ds/458
http://dx.doi.org/10.7488/ds/459
http://dx.doi.org/10.7488/ds/460
http://dx.doi.org/10.7488/ds/461
http://dx.doi.org/10.7488/ds/462
http://dx.doi.org/10.7488/ds/463
http://dx.doi.org/10.7488/ds/464
http://dx.doi.org/10.7488/ds/465
http://dx.doi.org/10.7488/ds/466
http://dx.doi.org/10.7488/ds/467
http://dx.doi.org/10.7488/ds/468
http://dx.doi.org/10.7488/ds/469
http://dx.doi.org/10.7488/ds/470

http://dx.doi.org/10.7488/ds/471
http://dx.doi.org/10.7488/ds/472
http://dx.doi.org/10.7488/ds/473
http://dx.doi.org/10.7488/ds/474
http://dx.doi.org/10.7488/ds/475
http://dx.doi.org/10.7488/ds/476
http://dx.doi.org/10.7488/ds/477
http://dx.doi.org/10.7488/ds/478
http://dx.doi.org/10.7488/ds/479
http://dx.doi.org/10.7488/ds/480
http://dx.doi.org/10.7488/ds/481
http://dx.doi.org/10.7488/ds/482
http://dx.doi.org/10.7488/ds/483
http://dx.doi.org/10.7488/ds/484
http://dx.doi.org/10.7488/ds/485
http://dx.doi.org/10.7488/ds/486
http://dx.doi.org/10.7488/ds/487
http://dx.doi.org/10.7488/ds/488
http://dx.doi.org/10.7488/ds/489
http://dx.doi.org/10.7488/ds/490
http://dx.doi.org/10.7488/ds/491
http://dx.doi.org/10.7488/ds/492
http://dx.doi.org/10.7488/ds/493
http://dx.doi.org/10.7488/ds/494
http://dx.doi.org/10.7488/ds/495
http://dx.doi.org/10.7488/ds/496
http://dx.doi.org/10.7488/ds/497
http://dx.doi.org/10.7488/ds/498
http://dx.doi.org/10.7488/ds/499
http://dx.doi.org/10.7488/ds/500
http://dx.doi.org/10.7488/ds/501
http://dx.doi.org/10.7488/ds/502
http://dx.doi.org/10.7488/ds/503
http://dx.doi.org/10.7488/ds/504
http://dx.doi.org/10.7488/ds/505
http://dx.doi.org/10.7488/ds/506
http://dx.doi.org/10.7488/ds/507
http://dx.doi.org/10.7488/ds/508
http://dx.doi.org/10.7488/ds/509
http://dx.doi.org/10.7488/ds/510
http://dx.doi.org/10.7488/ds/511

http://dx.doi.org/10.7488/ds/512
http://dx.doi.org/10.7488/ds/513
http://dx.doi.org/10.7488/ds/514
http://dx.doi.org/10.7488/ds/515
http://dx.doi.org/10.7488/ds/516
http://dx.doi.org/10.7488/ds/517
http://dx.doi.org/10.7488/ds/518
http://dx.doi.org/10.7488/ds/519
http://dx.doi.org/10.7488/ds/520
http://dx.doi.org/10.7488/ds/521
http://dx.doi.org/10.7488/ds/522
http://dx.doi.org/10.7488/ds/523
http://dx.doi.org/10.7488/ds/524
http://dx.doi.org/10.7488/ds/525
http://dx.doi.org/10.7488/ds/526
http://dx.doi.org/10.7488/ds/527
http://dx.doi.org/10.7488/ds/528
http://dx.doi.org/10.7488/ds/529
http://dx.doi.org/10.7488/ds/530
http://dx.doi.org/10.7488/ds/531
http://dx.doi.org/10.7488/ds/532
http://dx.doi.org/10.7488/ds/533
http://dx.doi.org/10.7488/ds/534
http://dx.doi.org/10.7488/ds/535
http://dx.doi.org/10.7488/ds/536
http://dx.doi.org/10.7488/ds/537
http://dx.doi.org/10.7488/ds/538
http://dx.doi.org/10.7488/ds/539
http://dx.doi.org/10.7488/ds/540
http://dx.doi.org/10.7488/ds/541
http://dx.doi.org/10.7488/ds/542
http://dx.doi.org/10.7488/ds/543
http://dx.doi.org/10.7488/ds/544
http://dx.doi.org/10.7488/ds/545
http://dx.doi.org/10.7488/ds/546
http://dx.doi.org/10.7488/ds/547
http://dx.doi.org/10.7488/ds/548
http://dx.doi.org/10.7488/ds/549
http://dx.doi.org/10.7488/ds/550
http://dx.doi.org/10.7488/ds/551
http://dx.doi.org/10.7488/ds/552

http://dx.doi.org/10.7488/ds/553
http://dx.doi.org/10.7488/ds/554
http://dx.doi.org/10.7488/ds/555
http://dx.doi.org/10.7488/ds/556
http://dx.doi.org/10.7488/ds/557
http://dx.doi.org/10.7488/ds/558
http://dx.doi.org/10.7488/ds/559
http://dx.doi.org/10.7488/ds/560
http://dx.doi.org/10.7488/ds/561
http://dx.doi.org/10.7488/ds/562
http://dx.doi.org/10.7488/ds/563
http://dx.doi.org/10.7488/ds/564
http://dx.doi.org/10.7488/ds/565
http://dx.doi.org/10.7488/ds/566
http://dx.doi.org/10.7488/ds/567
http://dx.doi.org/10.7488/ds/568
http://dx.doi.org/10.7488/ds/569
http://dx.doi.org/10.7488/ds/570
http://dx.doi.org/10.7488/ds/571
http://dx.doi.org/10.7488/ds/572
http://dx.doi.org/10.7488/ds/573
http://dx.doi.org/10.7488/ds/574
http://dx.doi.org/10.7488/ds/575
http://dx.doi.org/10.7488/ds/576
http://dx.doi.org/10.7488/ds/577
http://dx.doi.org/10.7488/ds/578
http://dx.doi.org/10.7488/ds/579
http://dx.doi.org/10.7488/ds/580
http://dx.doi.org/10.7488/ds/581
http://dx.doi.org/10.7488/ds/582
http://dx.doi.org/10.7488/ds/583
http://dx.doi.org/10.7488/ds/584
http://dx.doi.org/10.7488/ds/585
http://dx.doi.org/10.7488/ds/586
http://dx.doi.org/10.7488/ds/587
http://dx.doi.org/10.7488/ds/588
http://dx.doi.org/10.7488/ds/589
http://dx.doi.org/10.7488/ds/590
http://dx.doi.org/10.7488/ds/591
http://dx.doi.org/10.7488/ds/592
http://dx.doi.org/10.7488/ds/593

http://dx.doi.org/10.7488/ds/594
http://dx.doi.org/10.7488/ds/595
http://dx.doi.org/10.7488/ds/596
http://dx.doi.org/10.7488/ds/1322
http://dx.doi.org/10.7488/ds/1323
http://dx.doi.org/10.7488/ds/1324
http://dx.doi.org/10.7488/ds/1325
http://dx.doi.org/10.7488/ds/1326
http://dx.doi.org/10.7488/ds/1327
http://dx.doi.org/10.7488/ds/372
http://dx.doi.org/10.7488/ds/373
http://dx.doi.org/10.7488/ds/374
http://dx.doi.org/10.7488/ds/375
http://dx.doi.org/10.7488/ds/376
http://dx.doi.org/10.7488/ds/377
http://dx.doi.org/10.7488/ds/378
http://dx.doi.org/10.7488/ds/379
http://dx.doi.org/10.7488/ds/380
http://dx.doi.org/10.7488/ds/381
http://dx.doi.org/10.7488/ds/382
http://dx.doi.org/10.7488/ds/383
http://dx.doi.org/10.7488/ds/384
http://dx.doi.org/10.7488/ds/385
http://dx.doi.org/10.7488/ds/386
http://dx.doi.org/10.7488/ds/387
http://dx.doi.org/10.7488/ds/388
http://dx.doi.org/10.7488/ds/389
http://dx.doi.org/10.7488/ds/390
http://dx.doi.org/10.7488/ds/391
http://dx.doi.org/10.7488/ds/597
http://dx.doi.org/10.7488/ds/598
http://dx.doi.org/10.7488/ds/599
http://dx.doi.org/10.7488/ds/600
http://dx.doi.org/10.7488/ds/601
http://dx.doi.org/10.7488/ds/602
http://dx.doi.org/10.7488/ds/603
http://dx.doi.org/10.7488/ds/604
http://dx.doi.org/10.7488/ds/605
http://dx.doi.org/10.7488/ds/606
http://dx.doi.org/10.7488/ds/607
http://dx.doi.org/10.7488/ds/608

http://dx.doi.org/10.7488/ds/609
http://dx.doi.org/10.7488/ds/610
http://dx.doi.org/10.7488/ds/611
http://dx.doi.org/10.7488/ds/612
http://dx.doi.org/10.7488/ds/613
http://dx.doi.org/10.7488/ds/614
http://dx.doi.org/10.7488/ds/615
http://dx.doi.org/10.7488/ds/616
http://dx.doi.org/10.7488/ds/617
http://dx.doi.org/10.7488/ds/618
http://dx.doi.org/10.7488/ds/619
http://dx.doi.org/10.7488/ds/620
http://dx.doi.org/10.7488/ds/621
http://dx.doi.org/10.7488/ds/622
http://dx.doi.org/10.7488/ds/623
http://dx.doi.org/10.7488/ds/624
http://dx.doi.org/10.7488/ds/625
http://dx.doi.org/10.7488/ds/626
http://dx.doi.org/10.7488/ds/627
http://dx.doi.org/10.7488/ds/628
http://dx.doi.org/10.7488/ds/629
http://dx.doi.org/10.7488/ds/630
http://dx.doi.org/10.7488/ds/631
http://dx.doi.org/10.7488/ds/632
http://dx.doi.org/10.7488/ds/633
http://dx.doi.org/10.7488/ds/634
http://dx.doi.org/10.7488/ds/635
http://dx.doi.org/10.7488/ds/636
http://dx.doi.org/10.7488/ds/637
http://dx.doi.org/10.7488/ds/638
http://dx.doi.org/10.7488/ds/639
http://dx.doi.org/10.7488/ds/640
http://dx.doi.org/10.7488/ds/641
http://dx.doi.org/10.7488/ds/642
http://dx.doi.org/10.7488/ds/643
http://dx.doi.org/10.7488/ds/644
http://dx.doi.org/10.7488/ds/645
http://dx.doi.org/10.7488/ds/646
http://dx.doi.org/10.7488/ds/647
http://dx.doi.org/10.7488/ds/648
http://dx.doi.org/10.7488/ds/649

http://dx.doi.org/10.7488/ds/650
http://dx.doi.org/10.7488/ds/651
http://dx.doi.org/10.7488/ds/652
http://dx.doi.org/10.7488/ds/653
http://dx.doi.org/10.7488/ds/654
http://dx.doi.org/10.7488/ds/655
http://dx.doi.org/10.7488/ds/656
http://dx.doi.org/10.7488/ds/657
http://dx.doi.org/10.7488/ds/658
http://dx.doi.org/10.7488/ds/659
http://dx.doi.org/10.7488/ds/660
http://dx.doi.org/10.7488/ds/661
http://dx.doi.org/10.7488/ds/662
http://dx.doi.org/10.7488/ds/663
http://dx.doi.org/10.7488/ds/664
http://dx.doi.org/10.7488/ds/665
http://dx.doi.org/10.7488/ds/666
http://dx.doi.org/10.7488/ds/667
http://dx.doi.org/10.7488/ds/668
http://dx.doi.org/10.7488/ds/669
http://dx.doi.org/10.7488/ds/670
http://dx.doi.org/10.7488/ds/671
http://dx.doi.org/10.7488/ds/672
http://dx.doi.org/10.7488/ds/673
http://dx.doi.org/10.7488/ds/674
http://dx.doi.org/10.7488/ds/675
http://dx.doi.org/10.7488/ds/676
http://dx.doi.org/10.7488/ds/677
http://dx.doi.org/10.7488/ds/678
http://dx.doi.org/10.7488/ds/679
http://dx.doi.org/10.7488/ds/680
http://dx.doi.org/10.7488/ds/681
http://dx.doi.org/10.7488/ds/682
http://dx.doi.org/10.7488/ds/683
http://dx.doi.org/10.7488/ds/684
http://dx.doi.org/10.7488/ds/685
http://dx.doi.org/10.7488/ds/686
http://dx.doi.org/10.7488/ds/687
http://dx.doi.org/10.7488/ds/688
http://dx.doi.org/10.7488/ds/689
http://dx.doi.org/10.7488/ds/690

http://dx.doi.org/10.7488/ds/691
http://dx.doi.org/10.7488/ds/692
http://dx.doi.org/10.7488/ds/693
http://dx.doi.org/10.7488/ds/694
http://dx.doi.org/10.7488/ds/695
http://dx.doi.org/10.7488/ds/696
http://dx.doi.org/10.7488/ds/697
http://dx.doi.org/10.7488/ds/698
http://dx.doi.org/10.7488/ds/699
http://dx.doi.org/10.7488/ds/700
http://dx.doi.org/10.7488/ds/701
http://dx.doi.org/10.7488/ds/702
http://dx.doi.org/10.7488/ds/703
http://dx.doi.org/10.7488/ds/704
http://dx.doi.org/10.7488/ds/705
http://dx.doi.org/10.7488/ds/706
http://dx.doi.org/10.7488/ds/707
http://dx.doi.org/10.7488/ds/708
http://dx.doi.org/10.7488/ds/709
http://dx.doi.org/10.7488/ds/710
http://dx.doi.org/10.7488/ds/711
http://dx.doi.org/10.7488/ds/712
http://dx.doi.org/10.7488/ds/713
http://dx.doi.org/10.7488/ds/714
http://dx.doi.org/10.7488/ds/715
http://dx.doi.org/10.7488/ds/716
http://dx.doi.org/10.7488/ds/717
http://dx.doi.org/10.7488/ds/718
http://dx.doi.org/10.7488/ds/719
http://dx.doi.org/10.7488/ds/720
http://dx.doi.org/10.7488/ds/721
http://dx.doi.org/10.7488/ds/722
http://dx.doi.org/10.7488/ds/723
http://dx.doi.org/10.7488/ds/724
http://dx.doi.org/10.7488/ds/725
http://dx.doi.org/10.7488/ds/726
http://dx.doi.org/10.7488/ds/727
http://dx.doi.org/10.7488/ds/728
http://dx.doi.org/10.7488/ds/729
http://dx.doi.org/10.7488/ds/730
http://dx.doi.org/10.7488/ds/731

http://dx.doi.org/10.7488/ds/732
http://dx.doi.org/10.7488/ds/733
http://dx.doi.org/10.7488/ds/734
http://dx.doi.org/10.7488/ds/735
http://dx.doi.org/10.7488/ds/736
http://dx.doi.org/10.7488/ds/737
http://dx.doi.org/10.7488/ds/738
http://dx.doi.org/10.7488/ds/739
http://dx.doi.org/10.7488/ds/740
http://dx.doi.org/10.7488/ds/741
http://dx.doi.org/10.7488/ds/742
http://dx.doi.org/10.7488/ds/743
http://dx.doi.org/10.7488/ds/744
http://dx.doi.org/10.7488/ds/745
http://dx.doi.org/10.7488/ds/746
http://dx.doi.org/10.7488/ds/747
http://dx.doi.org/10.7488/ds/748
http://dx.doi.org/10.7488/ds/749
http://dx.doi.org/10.7488/ds/750
http://dx.doi.org/10.7488/ds/751
http://dx.doi.org/10.7488/ds/752
http://dx.doi.org/10.7488/ds/753
http://dx.doi.org/10.7488/ds/754
http://dx.doi.org/10.7488/ds/755
http://dx.doi.org/10.7488/ds/756
http://dx.doi.org/10.7488/ds/757
http://dx.doi.org/10.7488/ds/758
http://dx.doi.org/10.7488/ds/759
http://dx.doi.org/10.7488/ds/760
http://dx.doi.org/10.7488/ds/761
http://dx.doi.org/10.7488/ds/762
http://dx.doi.org/10.7488/ds/763
http://dx.doi.org/10.7488/ds/764
http://dx.doi.org/10.7488/ds/765
http://dx.doi.org/10.7488/ds/766
http://dx.doi.org/10.7488/ds/767
http://dx.doi.org/10.7488/ds/768
http://dx.doi.org/10.7488/ds/769
http://dx.doi.org/10.7488/ds/770
http://dx.doi.org/10.7488/ds/771
http://dx.doi.org/10.7488/ds/772

http://dx.doi.org/10.7488/ds/773
http://dx.doi.org/10.7488/ds/774
http://dx.doi.org/10.7488/ds/775
http://dx.doi.org/10.7488/ds/776
http://dx.doi.org/10.7488/ds/777
http://dx.doi.org/10.7488/ds/778
http://dx.doi.org/10.7488/ds/779
http://dx.doi.org/10.7488/ds/780
http://dx.doi.org/10.7488/ds/781
http://dx.doi.org/10.7488/ds/782
http://dx.doi.org/10.7488/ds/783
http://dx.doi.org/10.7488/ds/784
http://dx.doi.org/10.7488/ds/785
http://dx.doi.org/10.7488/ds/786
http://dx.doi.org/10.7488/ds/787
http://dx.doi.org/10.7488/ds/788
http://dx.doi.org/10.7488/ds/789
http://dx.doi.org/10.7488/ds/790
http://dx.doi.org/10.7488/ds/791
http://dx.doi.org/10.7488/ds/792
http://dx.doi.org/10.7488/ds/793
http://dx.doi.org/10.7488/ds/794
http://dx.doi.org/10.7488/ds/795
http://dx.doi.org/10.7488/ds/796
http://dx.doi.org/10.7488/ds/797
http://dx.doi.org/10.7488/ds/798
http://dx.doi.org/10.7488/ds/799
http://dx.doi.org/10.7488/ds/800
http://dx.doi.org/10.7488/ds/801
http://dx.doi.org/10.7488/ds/802
http://dx.doi.org/10.7488/ds/803
http://dx.doi.org/10.7488/ds/804
http://dx.doi.org/10.7488/ds/805
http://dx.doi.org/10.7488/ds/806
http://dx.doi.org/10.7488/ds/807
http://dx.doi.org/10.7488/ds/808
http://dx.doi.org/10.7488/ds/809
http://dx.doi.org/10.7488/ds/810
http://dx.doi.org/10.7488/ds/811
http://dx.doi.org/10.7488/ds/812
http://dx.doi.org/10.7488/ds/813

http://dx.doi.org/10.7488/ds/814
http://dx.doi.org/10.7488/ds/815
http://dx.doi.org/10.7488/ds/816
http://dx.doi.org/10.7488/ds/817
http://dx.doi.org/10.7488/ds/818
http://dx.doi.org/10.7488/ds/819
http://dx.doi.org/10.7488/ds/820
http://dx.doi.org/10.7488/ds/821
http://dx.doi.org/10.7488/ds/822
http://dx.doi.org/10.7488/ds/823
http://dx.doi.org/10.7488/ds/824
http://dx.doi.org/10.7488/ds/825
http://dx.doi.org/10.7488/ds/826
http://dx.doi.org/10.7488/ds/827
http://dx.doi.org/10.7488/ds/828
http://dx.doi.org/10.7488/ds/829
http://dx.doi.org/10.7488/ds/830
http://dx.doi.org/10.7488/ds/831
http://dx.doi.org/10.7488/ds/832
http://dx.doi.org/10.7488/ds/833
http://dx.doi.org/10.7488/ds/834
http://dx.doi.org/10.7488/ds/835
http://dx.doi.org/10.7488/ds/836
http://dx.doi.org/10.7488/ds/837
http://dx.doi.org/10.7488/ds/838
http://dx.doi.org/10.7488/ds/839
http://dx.doi.org/10.7488/ds/840
http://dx.doi.org/10.7488/ds/841
http://dx.doi.org/10.7488/ds/842
http://dx.doi.org/10.7488/ds/843
http://dx.doi.org/10.7488/ds/844
http://dx.doi.org/10.7488/ds/845
http://dx.doi.org/10.7488/ds/846
http://dx.doi.org/10.7488/ds/847
http://dx.doi.org/10.7488/ds/848
http://dx.doi.org/10.7488/ds/849
http://dx.doi.org/10.7488/ds/850
http://dx.doi.org/10.7488/ds/851
http://dx.doi.org/10.7488/ds/852
http://dx.doi.org/10.7488/ds/853
http://dx.doi.org/10.7488/ds/854

http://dx.doi.org/10.7488/ds/855
http://dx.doi.org/10.7488/ds/856
http://dx.doi.org/10.7488/ds/857
http://dx.doi.org/10.7488/ds/858
http://dx.doi.org/10.7488/ds/859
http://dx.doi.org/10.7488/ds/860
http://dx.doi.org/10.7488/ds/861
http://dx.doi.org/10.7488/ds/862
http://dx.doi.org/10.7488/ds/863
http://dx.doi.org/10.7488/ds/864
http://dx.doi.org/10.7488/ds/865
http://dx.doi.org/10.7488/ds/866
http://dx.doi.org/10.7488/ds/867
http://dx.doi.org/10.7488/ds/868
http://dx.doi.org/10.7488/ds/869
http://dx.doi.org/10.7488/ds/870
http://dx.doi.org/10.7488/ds/871
http://dx.doi.org/10.7488/ds/872
http://dx.doi.org/10.7488/ds/873
http://dx.doi.org/10.7488/ds/874
http://dx.doi.org/10.7488/ds/875
http://dx.doi.org/10.7488/ds/876
http://dx.doi.org/10.7488/ds/877
http://dx.doi.org/10.7488/ds/878
http://dx.doi.org/10.7488/ds/879
http://dx.doi.org/10.7488/ds/880
http://dx.doi.org/10.7488/ds/881
http://dx.doi.org/10.7488/ds/882
http://dx.doi.org/10.7488/ds/883
http://dx.doi.org/10.7488/ds/884
http://dx.doi.org/10.7488/ds/885
http://dx.doi.org/10.7488/ds/886
http://dx.doi.org/10.7488/ds/887
http://dx.doi.org/10.7488/ds/888
http://dx.doi.org/10.7488/ds/889
http://dx.doi.org/10.7488/ds/890
http://dx.doi.org/10.7488/ds/891
http://dx.doi.org/10.7488/ds/892
http://dx.doi.org/10.7488/ds/893
http://dx.doi.org/10.7488/ds/894
http://dx.doi.org/10.7488/ds/895

http://dx.doi.org/10.7488/ds/896
http://dx.doi.org/10.7488/ds/897
http://dx.doi.org/10.7488/ds/898
http://dx.doi.org/10.7488/ds/899
http://dx.doi.org/10.7488/ds/900
http://dx.doi.org/10.7488/ds/901
http://dx.doi.org/10.7488/ds/902
http://dx.doi.org/10.7488/ds/903
http://dx.doi.org/10.7488/ds/904
http://dx.doi.org/10.7488/ds/905
http://dx.doi.org/10.7488/ds/906
http://dx.doi.org/10.7488/ds/907
http://dx.doi.org/10.7488/ds/908
http://dx.doi.org/10.7488/ds/909
http://dx.doi.org/10.7488/ds/910
http://dx.doi.org/10.7488/ds/911
http://dx.doi.org/10.7488/ds/912
http://dx.doi.org/10.7488/ds/913
http://dx.doi.org/10.7488/ds/914
http://dx.doi.org/10.7488/ds/915
http://dx.doi.org/10.7488/ds/916
http://dx.doi.org/10.7488/ds/917
http://dx.doi.org/10.7488/ds/918
http://dx.doi.org/10.7488/ds/919
http://dx.doi.org/10.7488/ds/920
http://dx.doi.org/10.7488/ds/921
http://dx.doi.org/10.7488/ds/922
http://dx.doi.org/10.7488/ds/923
http://dx.doi.org/10.7488/ds/924
http://dx.doi.org/10.7488/ds/925
http://dx.doi.org/10.7488/ds/926
http://dx.doi.org/10.7488/ds/927
http://dx.doi.org/10.7488/ds/928
http://dx.doi.org/10.7488/ds/929
http://dx.doi.org/10.7488/ds/930
http://dx.doi.org/10.7488/ds/931
http://dx.doi.org/10.7488/ds/932
http://dx.doi.org/10.7488/ds/933
http://dx.doi.org/10.7488/ds/934
http://dx.doi.org/10.7488/ds/935
http://dx.doi.org/10.7488/ds/936

http://dx.doi.org/10.7488/ds/937
http://dx.doi.org/10.7488/ds/938
http://dx.doi.org/10.7488/ds/939
http://dx.doi.org/10.7488/ds/940
http://dx.doi.org/10.7488/ds/941
http://dx.doi.org/10.7488/ds/942
http://dx.doi.org/10.7488/ds/943
http://dx.doi.org/10.7488/ds/944
http://dx.doi.org/10.7488/ds/945
http://dx.doi.org/10.7488/ds/946
http://dx.doi.org/10.7488/ds/947
http://dx.doi.org/10.7488/ds/948
http://dx.doi.org/10.7488/ds/949
http://dx.doi.org/10.7488/ds/950
http://dx.doi.org/10.7488/ds/951
http://dx.doi.org/10.7488/ds/952
http://dx.doi.org/10.7488/ds/953
http://dx.doi.org/10.7488/ds/954
http://dx.doi.org/10.7488/ds/955
http://dx.doi.org/10.7488/ds/956
http://dx.doi.org/10.7488/ds/957
http://dx.doi.org/10.7488/ds/958
http://dx.doi.org/10.7488/ds/959
http://dx.doi.org/10.7488/ds/960
http://dx.doi.org/10.7488/ds/961
http://dx.doi.org/10.7488/ds/962
http://dx.doi.org/10.7488/ds/963
http://dx.doi.org/10.7488/ds/964
http://dx.doi.org/10.7488/ds/965
http://dx.doi.org/10.7488/ds/966
http://dx.doi.org/10.7488/ds/967
http://dx.doi.org/10.7488/ds/968
http://dx.doi.org/10.7488/ds/969
http://dx.doi.org/10.7488/ds/970
http://dx.doi.org/10.7488/ds/971
http://dx.doi.org/10.7488/ds/972
http://dx.doi.org/10.7488/ds/973
http://dx.doi.org/10.7488/ds/974
http://dx.doi.org/10.7488/ds/975
http://dx.doi.org/10.7488/ds/976
http://dx.doi.org/10.7488/ds/977

http://dx.doi.org/10.7488/ds/978
http://dx.doi.org/10.7488/ds/979
http://dx.doi.org/10.7488/ds/980
http://dx.doi.org/10.7488/ds/981
http://dx.doi.org/10.7488/ds/982
http://dx.doi.org/10.7488/ds/983
http://dx.doi.org/10.7488/ds/984
http://dx.doi.org/10.7488/ds/985
http://dx.doi.org/10.7488/ds/986
http://dx.doi.org/10.7488/ds/987
http://dx.doi.org/10.7488/ds/988
http://dx.doi.org/10.7488/ds/989
http://dx.doi.org/10.7488/ds/990
http://dx.doi.org/10.7488/ds/991
http://dx.doi.org/10.7488/ds/992
http://dx.doi.org/10.7488/ds/993
http://dx.doi.org/10.7488/ds/994
http://dx.doi.org/10.7488/ds/995
http://dx.doi.org/10.7488/ds/996
http://dx.doi.org/10.7488/ds/997
http://dx.doi.org/10.7488/ds/998
http://dx.doi.org/10.7488/ds/999
http://dx.doi.org/10.7488/ds/1000
http://dx.doi.org/10.7488/ds/1001
http://dx.doi.org/10.7488/ds/1002
http://dx.doi.org/10.7488/ds/1003
http://dx.doi.org/10.7488/ds/1004
http://dx.doi.org/10.7488/ds/1005
http://dx.doi.org/10.7488/ds/1006
http://dx.doi.org/10.7488/ds/1007
http://dx.doi.org/10.7488/ds/1008
http://dx.doi.org/10.7488/ds/1009
http://dx.doi.org/10.7488/ds/1010
http://dx.doi.org/10.7488/ds/1011
http://dx.doi.org/10.7488/ds/1012
http://dx.doi.org/10.7488/ds/1013
http://dx.doi.org/10.7488/ds/1014
http://dx.doi.org/10.7488/ds/1015
http://dx.doi.org/10.7488/ds/1016
http://dx.doi.org/10.7488/ds/1017
http://dx.doi.org/10.7488/ds/1018

http://dx.doi.org/10.7488/ds/1019
http://dx.doi.org/10.7488/ds/1020
http://dx.doi.org/10.7488/ds/1021
http://dx.doi.org/10.7488/ds/1022
http://dx.doi.org/10.7488/ds/1023
http://dx.doi.org/10.7488/ds/1024
http://dx.doi.org/10.7488/ds/1025
http://dx.doi.org/10.7488/ds/1026
http://dx.doi.org/10.7488/ds/1027
http://dx.doi.org/10.7488/ds/1028
http://dx.doi.org/10.7488/ds/1029
http://dx.doi.org/10.7488/ds/1030
http://dx.doi.org/10.7488/ds/1031
http://dx.doi.org/10.7488/ds/1032
http://dx.doi.org/10.7488/ds/1033
http://dx.doi.org/10.7488/ds/1034
http://dx.doi.org/10.7488/ds/1035
http://dx.doi.org/10.7488/ds/1036
http://dx.doi.org/10.7488/ds/1037
http://dx.doi.org/10.7488/ds/1038
http://dx.doi.org/10.7488/ds/1039
http://dx.doi.org/10.7488/ds/1040
http://dx.doi.org/10.7488/ds/1041
http://dx.doi.org/10.7488/ds/1042
http://dx.doi.org/10.7488/ds/1043
http://dx.doi.org/10.7488/ds/1044
http://dx.doi.org/10.7488/ds/1045
http://dx.doi.org/10.7488/ds/1046
http://dx.doi.org/10.7488/ds/1047
http://dx.doi.org/10.7488/ds/1048
http://dx.doi.org/10.7488/ds/1049
http://dx.doi.org/10.7488/ds/1050
http://dx.doi.org/10.7488/ds/1051
http://dx.doi.org/10.7488/ds/1052
http://dx.doi.org/10.7488/ds/1053
http://dx.doi.org/10.7488/ds/1054
http://dx.doi.org/10.7488/ds/1055
http://dx.doi.org/10.7488/ds/1056
http://dx.doi.org/10.7488/ds/1057
http://dx.doi.org/10.7488/ds/1058
http://dx.doi.org/10.7488/ds/1059

http://dx.doi.org/10.7488/ds/1060
http://dx.doi.org/10.7488/ds/1061
http://dx.doi.org/10.7488/ds/1062
http://dx.doi.org/10.7488/ds/1063
http://dx.doi.org/10.7488/ds/1064
http://dx.doi.org/10.7488/ds/1065
http://dx.doi.org/10.7488/ds/1066
http://dx.doi.org/10.7488/ds/1067
http://dx.doi.org/10.7488/ds/1068
http://dx.doi.org/10.7488/ds/1069
http://dx.doi.org/10.7488/ds/1070
http://dx.doi.org/10.7488/ds/1071
http://dx.doi.org/10.7488/ds/1072
http://dx.doi.org/10.7488/ds/1073
http://dx.doi.org/10.7488/ds/1074
http://dx.doi.org/10.7488/ds/1075
http://dx.doi.org/10.7488/ds/1076
http://dx.doi.org/10.7488/ds/1077
http://dx.doi.org/10.7488/ds/1078
http://dx.doi.org/10.7488/ds/1079
http://dx.doi.org/10.7488/ds/1080
http://dx.doi.org/10.7488/ds/1081
http://dx.doi.org/10.7488/ds/1082
http://dx.doi.org/10.7488/ds/1083
http://dx.doi.org/10.7488/ds/1084
http://dx.doi.org/10.7488/ds/1085
http://dx.doi.org/10.7488/ds/1086
http://dx.doi.org/10.7488/ds/1087
http://dx.doi.org/10.7488/ds/1088
http://dx.doi.org/10.7488/ds/1089
http://dx.doi.org/10.7488/ds/1090
http://dx.doi.org/10.7488/ds/1091
http://dx.doi.org/10.7488/ds/1092
http://dx.doi.org/10.7488/ds/1093
http://dx.doi.org/10.7488/ds/1094
http://dx.doi.org/10.7488/ds/1095
http://dx.doi.org/10.7488/ds/1096
http://dx.doi.org/10.7488/ds/1097
http://dx.doi.org/10.7488/ds/1098
http://dx.doi.org/10.7488/ds/1099
http://dx.doi.org/10.7488/ds/1100

http://dx.doi.org/10.7488/ds/1101
http://dx.doi.org/10.7488/ds/1102
http://dx.doi.org/10.7488/ds/1103
http://dx.doi.org/10.7488/ds/1104
http://dx.doi.org/10.7488/ds/1105
http://dx.doi.org/10.7488/ds/1106
http://dx.doi.org/10.7488/ds/1107
http://dx.doi.org/10.7488/ds/1108
http://dx.doi.org/10.7488/ds/1109
http://dx.doi.org/10.7488/ds/1110
http://dx.doi.org/10.7488/ds/1111
http://dx.doi.org/10.7488/ds/1112
http://dx.doi.org/10.7488/ds/1113
http://dx.doi.org/10.7488/ds/1114
http://dx.doi.org/10.7488/ds/1115
http://dx.doi.org/10.7488/ds/1116
http://dx.doi.org/10.7488/ds/1117
http://dx.doi.org/10.7488/ds/1118
http://dx.doi.org/10.7488/ds/1119
http://dx.doi.org/10.7488/ds/1120
http://dx.doi.org/10.7488/ds/1121
http://dx.doi.org/10.7488/ds/1122
http://dx.doi.org/10.7488/ds/1123
http://dx.doi.org/10.7488/ds/1124
http://dx.doi.org/10.7488/ds/1125
http://dx.doi.org/10.7488/ds/1126
http://dx.doi.org/10.7488/ds/1127
http://dx.doi.org/10.7488/ds/1128
http://dx.doi.org/10.7488/ds/1129
http://dx.doi.org/10.7488/ds/1130
http://dx.doi.org/10.7488/ds/1131
http://dx.doi.org/10.7488/ds/1132
http://dx.doi.org/10.7488/ds/1133
http://dx.doi.org/10.7488/ds/1134
http://dx.doi.org/10.7488/ds/1135
http://dx.doi.org/10.7488/ds/1136
http://dx.doi.org/10.7488/ds/1137
http://dx.doi.org/10.7488/ds/1138
http://dx.doi.org/10.7488/ds/1139
http://dx.doi.org/10.7488/ds/1140
http://dx.doi.org/10.7488/ds/1141

http://dx.doi.org/10.7488/ds/1142
http://dx.doi.org/10.7488/ds/1143
http://dx.doi.org/10.7488/ds/1144
http://dx.doi.org/10.7488/ds/1145
http://dx.doi.org/10.7488/ds/1146
http://dx.doi.org/10.7488/ds/1147
http://dx.doi.org/10.7488/ds/1148
http://dx.doi.org/10.7488/ds/1149
http://dx.doi.org/10.7488/ds/1150
http://dx.doi.org/10.7488/ds/1151
http://dx.doi.org/10.7488/ds/1152
http://dx.doi.org/10.7488/ds/1153
http://dx.doi.org/10.7488/ds/1154
http://dx.doi.org/10.7488/ds/1155
http://dx.doi.org/10.7488/ds/1156
http://dx.doi.org/10.7488/ds/1157
http://dx.doi.org/10.7488/ds/1158
http://dx.doi.org/10.7488/ds/1159
http://dx.doi.org/10.7488/ds/1160
http://dx.doi.org/10.7488/ds/1161
http://dx.doi.org/10.7488/ds/1162
http://dx.doi.org/10.7488/ds/1163
http://dx.doi.org/10.7488/ds/1164
http://dx.doi.org/10.7488/ds/1165
http://dx.doi.org/10.7488/ds/1166
http://dx.doi.org/10.7488/ds/1167
http://dx.doi.org/10.7488/ds/1168
http://dx.doi.org/10.7488/ds/1169
http://dx.doi.org/10.7488/ds/1170
http://dx.doi.org/10.7488/ds/1171
http://dx.doi.org/10.7488/ds/1172
http://dx.doi.org/10.7488/ds/1173
http://dx.doi.org/10.7488/ds/1174
http://dx.doi.org/10.7488/ds/1175
http://dx.doi.org/10.7488/ds/1176
http://dx.doi.org/10.7488/ds/1177
http://dx.doi.org/10.7488/ds/1178
http://dx.doi.org/10.7488/ds/1179
http://dx.doi.org/10.7488/ds/1180
http://dx.doi.org/10.7488/ds/1181
http://dx.doi.org/10.7488/ds/1182

http://dx.doi.org/10.7488/ds/1183
http://dx.doi.org/10.7488/ds/1184
http://dx.doi.org/10.7488/ds/1185
http://dx.doi.org/10.7488/ds/1186
http://dx.doi.org/10.7488/ds/1187
http://dx.doi.org/10.7488/ds/1188
http://dx.doi.org/10.7488/ds/1189
http://dx.doi.org/10.7488/ds/1190
http://dx.doi.org/10.7488/ds/1191
http://dx.doi.org/10.7488/ds/1192
http://dx.doi.org/10.7488/ds/1193
http://dx.doi.org/10.7488/ds/1194
http://dx.doi.org/10.7488/ds/1195
http://dx.doi.org/10.7488/ds/1196
http://dx.doi.org/10.7488/ds/1197
http://dx.doi.org/10.7488/ds/1198
http://dx.doi.org/10.7488/ds/1199
http://dx.doi.org/10.7488/ds/1200
http://dx.doi.org/10.7488/ds/1201
http://dx.doi.org/10.7488/ds/1202
http://dx.doi.org/10.7488/ds/1203
http://dx.doi.org/10.7488/ds/1204
http://dx.doi.org/10.7488/ds/1205
http://dx.doi.org/10.7488/ds/1206
http://dx.doi.org/10.7488/ds/1207
http://dx.doi.org/10.7488/ds/1208
http://dx.doi.org/10.7488/ds/1209
http://dx.doi.org/10.7488/ds/1210
http://dx.doi.org/10.7488/ds/1211
http://dx.doi.org/10.7488/ds/1212
http://dx.doi.org/10.7488/ds/1213
http://dx.doi.org/10.7488/ds/1214
http://dx.doi.org/10.7488/ds/1215
http://dx.doi.org/10.7488/ds/1216
http://dx.doi.org/10.7488/ds/1217
http://dx.doi.org/10.7488/ds/1218
http://dx.doi.org/10.7488/ds/1219
http://dx.doi.org/10.7488/ds/1220
http://dx.doi.org/10.7488/ds/1221
http://dx.doi.org/10.7488/ds/1222
http://dx.doi.org/10.7488/ds/1223

http://dx.doi.org/10.7488/ds/1224
http://dx.doi.org/10.7488/ds/1225
http://dx.doi.org/10.7488/ds/1226
http://dx.doi.org/10.7488/ds/1227
http://dx.doi.org/10.7488/ds/1228
http://dx.doi.org/10.7488/ds/1229
http://dx.doi.org/10.7488/ds/1230
http://dx.doi.org/10.7488/ds/1231
http://dx.doi.org/10.7488/ds/1232
http://dx.doi.org/10.7488/ds/1233
http://dx.doi.org/10.7488/ds/1234
http://dx.doi.org/10.7488/ds/1235
http://dx.doi.org/10.7488/ds/1236
http://dx.doi.org/10.7488/ds/1237
http://dx.doi.org/10.7488/ds/1238
http://dx.doi.org/10.7488/ds/1239
http://dx.doi.org/10.7488/ds/1240
http://dx.doi.org/10.7488/ds/1241
http://dx.doi.org/10.7488/ds/1242
http://dx.doi.org/10.7488/ds/1243
http://dx.doi.org/10.7488/ds/1244
http://dx.doi.org/10.7488/ds/1245
http://dx.doi.org/10.7488/ds/1246
http://dx.doi.org/10.7488/ds/1247
http://dx.doi.org/10.7488/ds/1248
http://dx.doi.org/10.7488/ds/1249
http://dx.doi.org/10.7488/ds/1250
http://dx.doi.org/10.7488/ds/1251
http://dx.doi.org/10.7488/ds/1252
http://dx.doi.org/10.7488/ds/1253
http://dx.doi.org/10.7488/ds/1254
http://dx.doi.org/10.7488/ds/1255
http://dx.doi.org/10.7488/ds/1256
http://dx.doi.org/10.7488/ds/1257
http://dx.doi.org/10.7488/ds/1258
http://dx.doi.org/10.7488/ds/1259
http://dx.doi.org/10.7488/ds/1260
http://dx.doi.org/10.7488/ds/1261
http://dx.doi.org/10.7488/ds/1262
http://dx.doi.org/10.7488/ds/1263
http://dx.doi.org/10.7488/ds/1264

http://dx.doi.org/10.7488/ds/1265
http://dx.doi.org/10.7488/ds/1266
http://dx.doi.org/10.7488/ds/1267
http://dx.doi.org/10.7488/ds/1268
http://dx.doi.org/10.7488/ds/1269
http://dx.doi.org/10.7488/ds/1270
http://dx.doi.org/10.7488/ds/1271
http://dx.doi.org/10.7488/ds/1272
http://dx.doi.org/10.7488/ds/1273
http://dx.doi.org/10.7488/ds/1274
http://dx.doi.org/10.7488/ds/1275
http://dx.doi.org/10.7488/ds/1276
http://dx.doi.org/10.7488/ds/1277
http://dx.doi.org/10.7488/ds/1278
http://dx.doi.org/10.7488/ds/1279
http://dx.doi.org/10.7488/ds/1280
http://dx.doi.org/10.7488/ds/1281
http://dx.doi.org/10.7488/ds/1282
http://dx.doi.org/10.7488/ds/1283
http://dx.doi.org/10.7488/ds/1284
http://dx.doi.org/10.7488/ds/1285
http://dx.doi.org/10.7488/ds/1286
http://dx.doi.org/10.7488/ds/1287
http://dx.doi.org/10.7488/ds/1288
http://dx.doi.org/10.7488/ds/1289
http://dx.doi.org/10.7488/ds/1290
http://dx.doi.org/10.7488/ds/1291
http://dx.doi.org/10.7488/ds/1292
http://dx.doi.org/10.7488/ds/1293
http://dx.doi.org/10.7488/ds/1294
http://dx.doi.org/10.7488/ds/1295
http://dx.doi.org/10.7488/ds/1296
http://dx.doi.org/10.7488/ds/1297
http://dx.doi.org/10.7488/ds/1298
http://dx.doi.org/10.7488/ds/1299
http://dx.doi.org/10.7488/ds/1300
http://dx.doi.org/10.7488/ds/1301
http://dx.doi.org/10.7488/ds/1302
http://dx.doi.org/10.7488/ds/1303
http://dx.doi.org/10.7488/ds/1304
http://dx.doi.org/10.7488/ds/1305
