# Supplementary material for: eHistology image and annotation data from the Kaufman Atlas of Mouse Development
Source: Gigascience. 2017 Dec 20;7(2):gix131. doi: 10.1093/gigascience/gix131 (PMC5827353; doi:10.1093/gigascience/gix131)
Supplement: GIGA-D-17-00086_Revision_1.pdf [file gix131_giga-d-17-00086_revision_1.pdf]

GigaScience

eHistology image and annotation data from the Kaufman Atlas of Mouse Development

--Manuscript Draft--

Manuscript Number:	GIGA-D-17-00086R1
Full Title:	eHistology image and annotation data from the Kaufman Atlas of Mouse Development
Article Type:	Data Note
Funding Information:	Medical Research Council — Prof Richard A Baldock
Abstract:	"The Atlas of Mouse Development" by Kaufman is a classic paper atlas that is the de facto standard for the definition of mouse embryo anatomy in the context of standard histological images. We have re-digitised the original H&E stained tissue sections used for the book at high resolution and transferred the hand-drawn annotations to digital form. We have augmented the annotations with standard ontological assignments (EMAPA anatomy) and made the data freely available via an online viewer (eHistology) and from the University of Edinburgh DataShare archive. The dataset captures and preserves the definitive anatomical knowledge of the original atlas, provides a core image set for deeper community annotation and teaching, and delivers a unique high-quality set of high-resolution histological images through mammalian development for manual and automated analysis.
Corresponding Author:	Chris Armit<br><br>UNITED KINGDOM
Corresponding Author Secondary Information:	
Corresponding Author's Institution:	
Corresponding Author's Secondary Institution:	
First Author:	Chris Armit
First Author Secondary Information:	
Order of Authors:	Chris Armit<br>Richard A Baldock
Order of Authors Secondary Information:	
Response to Reviewers:	Dear GigaScience,<br><br>Thank you for considering our manuscript 'eHistology image and annotation data from the Kaufman Atlas of Mouse Development' (GIGA-D_17_00086) as a Data Note. We have revised the manuscript to accommodate the comments from the editorial board and the reviewers. We address each of these comments below.<br><br>Editorial board comment 1:<br><br>I can support publication, as this is the first time the annotations, coordinates, and images themselves have been published in a semi-structured form (e.g., EMAPA:XXXXX) that others could reuse.<br><br>Response to editorial board:<br><br>We are grateful of your support for this publication.<br><br>Editorial board comment 2:<br><br>I'd at least question whether the University of Edinburgh provides the best, most reliable repository for these data.

Powered by Editorial Manager® and ProduXion Manager® from Aries Systems Corporation

Response to editorial board:

The editorial board make a very good point and we have removed the statement “We suggest that the longevity provided by the University of Edinburgh will exceed any other option not associated with a similar institution.”

We see considerable value in having the data additionally hosted in the GigaDB repository, and we have added the statement ‘In addition and for convenience these data are also hosted in the GigaDB repository.’ If GigaDB were willing to host these data then would be delighted to additionally archive this dataset with you.

Editorial board comment 3:

The description of the ma-tech github repo needs much more detail.

Response to editorial board:

We have extended the ‘Code availability’ section of the manuscript and now refer to the WlzIIPSrv tiled image server and the eAtlasViewer javascript application. In addition, the github repository has been updated to include these applications.

Editorial board comment 4:

A clearer competing interest statement and information on the commercially annotations would also be useful, as well as more rationale on how this open resource differs.

Response to editorial board:

We have extended the competing interest statement to emphasise the open access nature of this dataset. Furthermore, we have revised the main body of the text to include examples of how the high-resolution images that we captured differ from those available in the print-version of the atlas. Specifically, we illustrate how the high-resolution atlas images can be used to morphologically identify mitotic and apoptotic cells in compartments of the developing embryo. This is not possible with the print-version of the atlas. To further emphasise this point, we now include an additional figure that details the rich information that is to be gained from capturing images at cellular-resolution.

Reviewer 1 comment 1:
The eHistology Viewer is mentioned in the abstract and shown in the figure. It would be helpful to include a URL link to that tool. (Right now there is only the link to the GitHub repository.)

Response to reviewer:

We now include a section on the ‘eHistology viewer’. This includes URLs, and additionally describes linking between Edinburgh Datashare and eHistology.

Reviewer 1 comment 2:

In part b of the figure (showing the pop-up box), taking that screenshot over white background -- instead of showing the semi-transparent view of the image in the background -- would clarify what is in that box.

Response to reviewer:

The reviewer makes a very good point and we have revised the figure to accommodate this change.

Reviewer 1 comment 3:

Powered by Editorial Manager® and ProduXion Manager® from Aries Systems Corporation

In Background & Summary, the wording "Providing for a secure (in terms of data preservation) and long-term accessibility…" is a bit awkward.

Response to reviewer:

We agree with the reviewer and we have revised this sentence to read:
Providing secure and long-term accessibility for research data is a difficult problem.

Reviewer 1 comment 4:

In Usage Notes, there appears to be some miswording, or perhaps a missing word, in "…which can be used to identify the set of physical glass slides help by the University of Edinburgh on which each histological section can be found."

Response to reviewer:

This was a type error and has been corrected. Furthermore, we have revised this section to expand on the physical location of the original glass slides. The amended sentence now reads as follows:
The 'source' can be used to identify the set of physical glass slides, archived with the Centre for Research Collections of the University of Edinburgh, on which each histological section can be found.

Reviewer 2 comment 1:

The annotation technology is not new and has been used and published for other histological images. Therefore, I am not sure what the point of this manuscript is other than that this technology has been applied to a popular, previously published resource. However, that has already been reported by Graham et al in a descriptive 2015 paper in the journal Development.

Response to reviewer:

The reviewer makes a very good point. However, there are significant differences between the original print-version of Kaufman's 'The Atlas of Mouse Development' and the image set that we have delivered. In the original version Kaufman chose a series of sections to be photographed, mounted on board and hand annotated using Letraset, then re-photographed to produce the grayscale plates used in the book. We obtained the original glass slides that were used in the Kaufman's 'The Atlas of Mouse Development' and we digitized these slides at high-resolution as there is added value in being able to visualise these sections in colour and at cellular-resolution. To emphasise the importance of the high-resolution version of the atlas, we now include an additional figure to illustrate how the cellular-resolution images that we have generated can be used to morphologically identify mitotic and apoptotic cells in compartments of the developing embryo. This is not possible with the print-version of the atlas.

The WlzIIPSrv tiled image server and the eAtlasViewer javascript application that we developed enabled point annotations to be added to the high-resolution images that we captured. The Graham et al (2015) Spotlight article in Development announced the eHistology resource and provided an overview of the eHistology viewer. However it was beyond the scope of this article to describe secure and long-term accessibility for image data that has been annotated in this way. In the current manuscript, we describe long-term accessibility to high-resolution images and supporting annotation of a highly accessed de facto atlas of mouse development, and we have described the approach we have adopted for data provenance. Furthermore, we now make available additional information that was not discussed in the Spotlight article. These include details of source, position, and pixel resolution that would be needed for automated analysis of this image dataset. We have revised the 'Usage notes' to accommodate the reviewer's comments, and we additionally include these data in a revised supplementary file. We hope this satisfies the reviewer.

Reviewer 2 comment 2:

	One of the stated reasons for publishing the manuscript is the author's concern that data published online by public institutions likes universities might become lost if funding for maintaining the resource is no longer available. However, the high-resolution scans of Kaufman's images and the annotations have also been published commercially and, therefore, this concern does not apply to this work.<br>As the described data and work is available for sale by Elsevier, I need to question the author's statement that "they have no competing interests in the publication" of this manuscript.<br><br>Response to reviewer:<br><br>The reviewer makes a very good point and we have removed the statement “We suggest that the longevity provided by the University of Edinburgh will exceed any other option not associated with a similar institution.” However, the statement by the reviewer that “the high-resolution scans of Kaufman's images and the annotations have also been published commercially” is incorrect. The high-resolution digitized images that we have generated and the associated image-coordinates for each annotation are fully freely available under a Creative Commons CC BY 4.0 licence and nowhere else. We have an agreement with Elsevier to present the images in a form similar to the original atlas plate layout on the eHistology web resource. We have revised the main body of the text to emphasise the open access nature of the digitized high-resolution image dataset, and we have extended our ‘Competing interests’ statement to accommodate the reviewer’s concerns.
**Additional Information:**	
**Question**	**Response**
Are you submitting this manuscript to a special series or article collection?	No
**Experimental design and statistics**<br><br>Full details of the experimental design and statistical methods used should be given in the Methods section, as detailed in our Minimum Standards Reporting Checklist. Information essential to interpreting the data presented should be made available in the figure legends.<br><br>Have you included all the information requested in your manuscript?	Yes
**Resources**<br><br>A description of all resources used, including antibodies, cell lines, animals and software tools, with enough information to allow them to be uniquely identified, should be included in the Methods section. Authors are strongly encouraged to cite Research Resource Identifiers (RRIDs) for antibodies, model organisms and tools, where possible.<br><br>Have you included the information	Yes

requested as detailed in our Minimum Standards Reporting Checklist?	
**Availability of data and materials**<br><br>All datasets and code on which the conclusions of the paper rely must be either included in your submission or deposited in publicly available repositories (where available and ethically appropriate), referencing such data using a unique identifier in the references and in the “Availability of Data and Materials” section of your manuscript.<br><br>Have you have met the above requirement as detailed in our Minimum Standards Reporting Checklist?	Yes

Powered by Editorial Manager® and ProduXion Manager® from Aries Systems Corporation

Manuscript
Click here to download Manuscript GigaScience_GIGA_D_17_00086_revised_13Dec2017.docx

Title

eHistology image and annotation data from the Kaufman Atlas of Mouse Development

Authors

Richard A Baldock[1] [ORCID:0000-0003-0332-6877] and Chris Armit[1] [0000-0002-9952-8141]

Affiliations

1. MRC Human Genetics Unit, Institute of Genomic and Molecular Medicine, University of Edinburgh, Crewe Road, Edinburgh EH4 2XU, UK

corresponding author: Richard Baldock (Richard.Baldock@igmm.ed.ac.uk)

Abstract

"The Atlas of Mouse Development" by Kaufman is a classic paper atlas that is the *de facto* standard for the definition of mouse embryo anatomy in the context of standard histological images. We have re-digitised the original H&E stained tissue sections used for the book at high resolution and transferred the hand-drawn annotations to digital form. We have augmented the annotations with standard ontological assignments (EMAPA anatomy) and made the data freely available via an online viewer (eHistology) and from the University of Edinburgh DataShare archive. The dataset captures and preserves the definitive anatomical knowledge of the original atlas, provides a core image set for deeper community annotation and teaching, and delivers a unique high-quality set of high-resolution histological images through mammalian development for manual and automated analysis.

Keywords

Embryo; Mouse; Section; Atlas; Anatomy; Imaging

Data Description

"The Atlas of Mouse Development"[1] is a book detailing the anatomy of mouse embryo development and stands as the definitive work in the field. The atlas is based on a lifetime of work by Kaufman who established a unique set of histological sections of about 450 mouse embryos, many of which are full serial section-series, from which he selected carefully staged samples for the histological images within the book. The combination of the histological section series and the printed book represent a unique resource and captures the current understanding of classical mouse anatomy. In taxonomic terms these physical sections are the *reference specimens* for the definition of mouse embryo anatomy and the digitised images with the associated annotations are a digital *holotype* for the definition of anatomical terms and the progression of mouse embryo development. In addition the paper atlas has given rise to the Mouse Atlas programme in Edinburgh [2] and to the EMAPA mouse anatomy ontology [3,4]. The original index for the book was used to develop the primary list of anatomical terms in the ontology, and EMAPA is now recognised as the standard mouse embryo ontology used to annotate mouse embryo data including embryo phenotype data [5,6].

In generating the eHistology Atlas, new images of the histological sections were acquired at high resolution, and the annotations have been transferred to a database. These images and annotations are now freely available from the eMouseAtlas web resource as eHistology (see figure 1) and have been described by Graham et al. [7]. The new high-resolution images and the associated image-coordinates for each annotation are fully freely available under a Creative Commons CC BY 4.0 licence. In addition we have an agreement with Elsevier to present the images in a form similar to the original atlas plate layout (the web resource) and Elsevier are able to use the new images for their own version of an online version if they want to. Here we describe the dataset of the 937 high-resolution histology images with anatomy annotations and how they have been made available for further study and analysis.

The motivation for the eHistology resource was to capture the anatomical knowledge in a permanently accessible open and digital form delivered with a viewer providing a view of the underlying histology data not possible in the printed atlas. The high-resolution images provide a rich resource of carefully staged mouse histology, which could be used for deeper analysis of tissue development and as a teaching resource. See figure 1 for an illustration of the resolution now available for these images. Embryogenesis is a highly dynamic process and in figure 2 we highlight some of the advantages of capturing images at cellular-resolution, for example the ability to zoom in and morphologically identify mitotically dividing cells and apoptotic cells undergoing programmed cell. This is simply not possible in the print-version of the atlas and represents a significant contribution to the community.

All the data is available under a creative commons licence (CC BY 4.0). In the future, we envisage the annotations being extended on a tissue-by-tissue basis through community curation. The eHistology viewer is open-source and is available from the Mouse Atlas technical GitHub repository (github.com/ma-tech) (eHistology ; RRID:SCR_015887).

Providing secure and long-term accessibility for research data is a difficult problem. A recent study of the longevity of 375 biomedical resources/databases [8] available on the web in 1997 found that 62.3% had ceased to be available, 14.4% were static and only 23.3% available as an active resource. The authors concluded that survival depended primarily on Institutional interest and that a strategy dependent on external funding will very likely fail. To ensure long-term preservation of the image data and supporting annotations, we have therefore registered this dataset with the University of Edinburgh *DataShare* (http://datashare.is.ed.ac.uk/) repository [9] with policies registered in OpenDOAR

(Directory of Open Access Repositories, http://www.opendoar.org/find.php?rID=1176&format=full). Specifically the preservation policy includes indefinite preservation of the original data with format migration to ensure continued readability and accessibility. In addition and for convenience these data are also hosted in the GigaDB repository[10].

Methods

Histology

Details of the mouse strains used, histological sectioning and staining are provided by Kaufman (1994)[1]. Briefly the embryos were "isolated from spontaneously cycling (C57BL X CBA) F1 hybrid females that had been previously mated to genetically similar F1 hybrid males". The embryos were fixed, dehydrated, embedded in paraffin wax and sectioned at seven-micron thickness. The mounted sections were then stained with haematoxylin and eosin.

Slide digitization

Digitization of the original histology slides was accomplished using the Olympus DotSlide slide scanner system. Using a ×20 objective lens, this generated full colour images with a pixel resolution of 0.34 microns. Calibration was accomplished as part of the digitization process, allowing the inclusion of scale-bars and the option to measure the distance between two points. In two instances (Plate 5 and Plate 14) the original sections could not be sourced and were presumed lost. In these instances the original photographic negatives were used in place of the original slides to generate cellular-resolution grey-scale images.

Annotation and linking to the EMAPA ontology

Annotation was accomplished using a manual procedure whereby "flags" were positioned on points corresponding to the matching points as used in each plate in the book. The flags were placed using an editors version of the eHistology interface [7]. Each flag was linked to the anatomical term or phrase used in the book and also an EMAPA ontology term and an associated Wikipedia link. There were over 10,000 flag labels used to annotate the eHistology sections, and linking them to EMAPA IDs was achieved through a combination of string matching and manual assignment of terms [11]. Linking to Wikipedia was accomplished using a manual process that utilized parent terms in the *partonomic* ontology tree to find the closest match for a given anatomical term or tissue.

eHistology viewer

Each eHistology image is described in an Edinburgh DataShare DOI, and this description includes the URL link to the eHistology viewer for that image. In this way, we provide a persistent means of accessing the zoom viewer for that image. An example DOI for a single high-resolution image is: dx.doi.org/10.7488/ds/1232. This link resolves to a specific page at the Edinburgh Datashare web resource, datashare.is.ed.ac.uk/handle/10283/1807 [12], which in turn provides a link through to the current URL for the eHistology viewer www.emouseatlas.org/eAtlasViewer_ema/application/ema/kaufman/plate_38b.php?image=b. By starting with a fully persistent DOI the user will always be able to locate the data and is protected from any change to the hosting domain and URL of the eHistology viewer. For convenience we also provide an interactive index to the new images based on the plate and image designations of the original atlas.

Code availability

The data is provided in open-standard tif or jpeg image formats. All metadata is in plain txt format and the supplementary file is in Microsoft Excel the open xml format xlsx. Code used for the online histology viewer is provided at the ma-tech GitHub archive and specifically we use the WlzIIPSrv tiled image server and the eAtlasViewer javascript application.

Data Records

Each record has an assigned Digital Object Identifier (DOI) that resolves to a set of data files comprising a jpeg or tiff encoded image, Dublin core and other metadata files and the set of annotations associated with the image. The image data volumes range up to 2Gb with a total volume of 118GB for the full series in compressed “zip” format. Table 1 lists the files with each dataset. Each University of Edinburgh DataShare submission requires a subset of the Dublin Core [dublincore.org] data elements to be completed and allows a further set of optional elements these are detailed in table 2. Table 3 provides a partial listing of the datasets available as an example of the data content. The full listing of all 937 images is provided in the supplementary Excel formatted data file SciDataKaufmanTable3.xlsx and corresponds to all of the histology section images of the original atlas for Plate numbers 2-41.

Table 1. Listing of the data files available with each dataset.

File	Description
**license.txt**	Licence agreement for the data deposited at DataShare providing permissions for distribution and migration as needed - CC BY 4.0.
**README.txt**	Short description of the data and data files.
**citation.txt**	How to cite use of this particular image.
**details.txt**	The text describing the embryo taken from the matching page of the printed atlas and provided in tab-delimited form for reading into a spreadsheet programme.
**image.tif/image.jpg**	Full resolution tiff or jpeg formatted image of the histological section.
**Image.txt**	Image pixel dimensions and pixel size in microns.
**terms.txt**	A tab-delimited table of the annotations for this image providing the Kaufman annotations, location in the image, annotation number, EMAPA ID and EMAPA term with synonyms in brackets.
**url.txt**	Text providing the URL for the image on the emousetlas.org web resource.

Table 2. DataShare Dublin Core elements used for the Kaufman datasets. The DCMI column provides the official Dublin Core term for the element, the Label is the heading for these data on the DataShare metadata listing.

Element	Qualifier	DCMI	Label	Input Type	Mandatory
**Contributor**		contributor	Depositor	name	true
**Contributor**	other	contributor	Funder	name	true
**Creator**		creator	Data Creator	name	false
**Date**	accessioned	Date	Date Accessioned	date	true
**date**	available	Date	Date Available	date	true
**Identifier**	citation	identifier	Citation	citation	false
**Identifier**	uri	identifier	Persistent Identifier	DOI/handle	true
**Description**	abstract	decription	Data Description (abstract)	text	false
**Description**	tableofcontents	decription	Data Description (TOC)	text	false
**Publisher**		publisher	Publisher	text	true
**Relation**	isversionof	isVersionOf	Relation (Is Version Of)	text	false
**Relation**	isreferencedby	isReferencedBy	Relation (Is Referenced By)	text	false
**Subject**		subject	Subject Keywords	text	false
**Subject**	classification	subject	Subject Classification	Controlled text	false
**Title**		Title	Title	text	true
**Title**	alternative	Title	Alternative Title	text	false
**Type**		Type	Type	Controlled text	true

Table 3. Partial list of data records for the Kaufman Atlas image set. The DOI resolves to a data set of image data, metadata and annotations, which can be downloaded individually or combined. Image volumes range up to about 2Gb with a total volume for the full set of compressed zip files 118Gb. The Position column gives an estimate of the relative distance through the embryo of the individual histology section. The values are between 0 (zero) and 1 (one) corresponding to the proportionate distance left-right (sagittal sections), cranial-caudal (transverse sections) and dorsal-ventral (coronal sections).

Kaufman Image	Age	Stage	Orientation	Position	Stain	DOI
Plate 02 image a	E5.5	7-8	Sagittal		H&E	http://dx.doi.org/10.7488/ds/393
Plate 02 image b	E5.5	7-8	Sagittal		H&E	http://dx.doi.org/10.7488/ds/394
Plate 02 image c	E5.5	7-8	Sagittal		H&E	http://dx.doi.org/10.7488/ds/395
Plate 02 image d	E5.5	7-8	Sagittal		H&E	http://dx.doi.org/10.7488/ds/396
Plate 03 image a	E6.5,7	9,10	Transverse	0.210	H&E	http://dx.doi.org/10.7488/ds/397
Plate 03 image b	E6.5,7	9,10	Transverse	0.370	H&E	http://dx.doi.org/10.7488/ds/398
Plate 03 image c	E6.5,7	9,10	Transverse	0.590	H&E	http://dx.doi.org/10.7488/ds/399
.	.	.	.	.	.	.
.	.	.	.	.	.	.
Full table in supplementary Excel file SciDataKaufmanTable3.xlsx						
.	.	.	.	.	.	.
.	.	.	.	.	.	.
.	.	.	.	.	.	.
Plate 40l image b	E17.5	26	Transverse	0.888	H&E	http://dx.doi.org/10.7488/ds/1301
Plate 40l image c	E17.5	26	Transverse	0.903	H&E	http://dx.doi.org/10.7488/ds/1302
Plate 40l image d	E17.5	26	Transverse	0.921	H&E	http://dx.doi.org/10.7488/ds/1303
Plate 40l image e	E17.5	26	Transverse	0.948	H&E	http://dx.doi.org/10.7488/ds/1304
Plate 41 image 41	E17.5	26	Sagittal	0.496	H&E	http://dx.doi.org/10.7488/ds/1305

Technical Validation

The Images and associated data are all validated against the published atlas, which provides the detail of the genotype, defines the histological protocols, and establishes the correct staging of each embryo against the Theiler criteria. The section images used in the book are from specific tissue sections identified on the sets of microscope slides stored at the MRC Human Genetics Unit at the IGMM, University of Edinburgh. Each section was scanned digitally then checked by a second curator to ensure validity. The annotations were originally captured using optical character recognition (OCR) and the text and spelling checked by a second curator. All the end-point locations for the annotation terms have been double-checked and a series of quality control steps have meant that inspection of the whole data set has not revealed any errors.

Usage Notes

There are no constraints on the use of the images and associated data. The supplementary file lists all samples and assays - one for each section image - and also a ‘source’, which is the embryonic mouse specimen used by Kaufman in producing the histological sections. The ‘source’ can be used to identify the set of physical glass slides, archived with the Centre for Research Collections of the University of Edinburgh, on which each histological section can be found. In principle it is possible to obtain further images of the same or other sections in the series. ‘Age’ is defined in embryonic days post-coitum, and ‘stage’ refers to Theiler stage, a morphological staging system used to further define mouse embryo development. ‘Position’ describes the relative position of the section in the embryo, with 0 representing, for example, the most cranial section in a transverse series and 1 denoting the most caudal

section. We additionally include details of the pixel resolution of each image, enabling accurate measurements to be made on each high-resolution embryo atlas image.

Availability of source code and requirements

Project name: eHistology
Project home page: http://www.emouseatlas.org/emap/eHistology/ [RRID:SCR_015887]; https://github.com/ma-tech/eHistologyWebapp
Operating system(s): Platform independent
Programming language: The IIP Image server is a Fast CGI module written in C++
Other requirements: The IIP Image server can be embedded within a host web server such as Apache, Lighttpd, MyServer or Nginx.
License: Software - GNU General Public License v2.0; Image Data - CC BY 4.0

Availability of supporting data

The image datasets described in this article are available at "Edinburgh DataShare" repository as Plates 02-15 (early gestation; E5.5-E8.5), Plates 16-29 (mid-gestation; E8.5-E13.0); Plates 30-41 (late gestation; E13.5-E17.5); and Plates S1-S6 (coronal supplement; E11.5-E15.5)[13]. Each image dataset is provided with all the information needed to generate the annotated views shown on the eHistology website. The data are provided as a series of files, including: details.txt – a tab-delimited file with the embryo text description that appears at the top of the printed atlas page; image.jpg - full resolution jpeg image of the histological section (in some cases this is a tif image); terms.txt – a tab-delimited file with the annotations for this image, including location of point annotation (x, y coordinates), and EMAPA ID and EMAPA terms associated with that point. Edinburgh DataShare has provided data DOIs for each of these image datasets, and links to these data DOIs are additionally hosted in GigaDB. An archival copy of the GitHub repository with software tools for the eHistology application is additionally available in GigaDB[10].

List of abbreviations

H&E – haematoxylin and eosin
EMAPA – Edinburgh Mouse Atlas Project Anatomy

Competing interests

The authors declare they have no competing interests in the publication of this data and manuscript. The new high-resolution images and the associated image-coordinates for each annotation are fully freely available under a Creative Commons CC BY 4.0 licence. We have an agreement with Elsevier to present the images in a form similar to the original atlas plate layout on the eHistology web resource. However, this does not impact on re-use of the image data and annotation described in this manuscript.

Funding

The MRC Human Genetics Unit Mouse Atlas Programme was core-funded by the Medical Research Council (Awardee RA Baldock).

Authors' contributions

RAB leads the Mouse Atlas programme that generated these datasets, designed the dataset submissions to the DataShare system and wrote the scripts providing the upload formats needed for batch ingest to DataShare. He also wrote the first draft of the manuscript.

CA is the senior editor for the Mouse Atlas databases, co-wrote the manuscript and has performed much of the quality control on the datasets.

Acknowledgements

The authors would like to acknowledge the critical contribution to the collection, collation and curation of these data by Liz Graham and Julie Moss and to the development of the data visualisation aspects by Nick Burton. All these are co-authors on the key citation for this work [7]. The authors would also like to thank the support of the Edina DataShare team, in particular Pauline Ward and Robin Rice. Finally this work has been funded as part of the MRC core-funded Mouse Atlas Programme at the MRC Human Genetics Unit, Edinburgh.

References

1. Kaufman, MH. The Atlas of Mouse Development**.** *Elsevier Academic Press, Amsterdam, The Netherlands*. (1994).
2. Richardson, L., Venkataraman, S., Stevenson, P., Yang, Y., Moss, J., Graham, L., Burton, N., Hill, B., Rao, J., Baldock, RA. & Armit, C. EMAGE mouse embryo spatial gene expression database: (2014 update). *Nucleic Acids Res*. **42(1)** D835-44 (2014). doi: 10.1093/nar/gkt1155
3. Hayamizu, TF., Wicks, MN., Davidson, DR., Burger, A., Ringwald, M. & Baldock, RA. EMAP/EMAPA ontology of mouse developmental anatomy: 2013 update*, Journal of Biomedical Semantics.* **4:15**. (2013).
4. Hayamizu, TF., Baldock, RA. & Ringwald, M., Mouse anatomy ontologies: enhancements and tools for exploring and integrating biomedical data, *Mamm Genome*. **26**: 422–430 (2015).
5. Brown, SD. & Moore, MW. Towards an encyclopaedia of mammalian gene function: the International Mouse Phenotyping Consortium. *Dis Model Mech* **5**(3), 289–92 (2012).
6. Mohun, T., Adams, DJ., Baldock, R., Bhattacharya, S., Copp, AJ., Hemberger, M., Houart, C., Hurles, ME., Robertson, E., Smith, JC., Weaver, T. & Weninger, W. Deciphering the Mechanisms of Developmental Disorders (DMDD): a new programme for phenotyping embryonic lethal mice. *Disease Models & Mechanisms* **6**, 562-566 (2013).
7. Graham, E., Moss, J., Burton, N., Roochun, Y., Armit, C., Lorna Richardson, L. & Baldock, R. The atlas of mouse development eHistology resource. *Development* **142**, 1909-1911 (2015).
8. Attwood, TK., Agit, B. & Ellis, ABM. Longevity of Biological Databases. *EMBnet.journal* **21,** e803 (2015). http://dx.doi.org/10.14806/ej.21.0.803
9. Rice, R. Edinburgh datashare – reflections from a data repository manager. *Bull. Assoc. Information Science and technology* **40(2)** 39-40 (2014). DOI: 10.1002/bult.2014.1720400212
10. Baldock, R, A; Armit, C (2017): Supporting data for "eHistology image and annotation data from the Kaufman Atlas of Mouse Development" GigaScience Database. http://dx.doi.org/10.5524/100365
11. Richardson L, Moss J, Graham E, Burton N, Armit C, Baldock R (2015) Developing the eHistology Atlas. *Database (Oxford) 2015.* . **bav105** doi: 10.1093/database/bav105

12. Elizabeth Graham; Julie Moss; Nick Burton; Yogmatee Roochun; Chris Armit; Lorna Richardson; Richard Baldock. (2015). eHistology Kaufman Atlas Plate 38b image b, [image]. University of Edinburgh. College of Medicine and Veterinary Medicine. http://dx.doi.org/10.7488/ds/1232.
13. eHistology Atlas with Kaufman Annotations https://datashare.is.ed.ac.uk/handle/10283/822

Figure Legends

Figure 1. Kaufman Atlas eHistology Viewer. a) screen shot showing the user interface with annotation list. The 3 selected terms showing as a blue “flags” number 37, 38, 39 are over the developing kidney. b) The pop-up dialog with extra information on selecting the “37”. c), d), e) progressively higher resolution images corresponding to zooming-in on the image. At full resolution the pixel spacing is 0.34 x 0.34 microns and reveals cellular architecture and arrangements.

Figure 2. Observing mitosis and apoptosis in cellular-resolution eHistology atlas images. A key advantage of capturing histology images at high-resolution is the ability to morphologically identify mitotically dividing cells and apoptotic cells in embryo atlas images. a) Zoomed-out view of a coronal image of a E14.5 embryo. b) On the zoomed-in view, neuroblasts in the ventricular zone of the cerebral cortex show intense haematoxylin staining (white arrows), a morphological feature associated with chromosome condensation in mitotically dividing cells. c) On the zoomed-in view, scattered cells in the interdigital zone of the forelimb are pyknotic (black arrows), a morphological feature associated with nuclear condensation. The pyknotic cells additionally show signs of cell shrinkage. These are morphological hallmarks of apoptosis.

Tables

Table legends from above:

Table 1. Listing of the data files available with each dataset.

Table 2. DataShare Dublin Core elements used for the Kaufman datasets. The DCMI column provides the official Dublin Core term for the element, the Label is the heading for this data on the DataShare metadata listing.

Table 3. Complete list of data records for the Kaufman Atlas image set. The DOI resolves to a data set of image data, metadata and annotations, which can be downloaded individually or combined. Image volumes range up to about 2Gb with a total volume for the full set of compressed zip files 118Gb. The Position column gives an estimate of the relative distance through the embryo of the individual histology section. The values are between 0 (zero) and 1 (one) corresponding to the proportionate distance left-right (sagittal sections), cranial-caudal (transverse sections) and dorsal-ventral (coronal sections).

Figure 1

Click here to download Figure Fig_01.tif

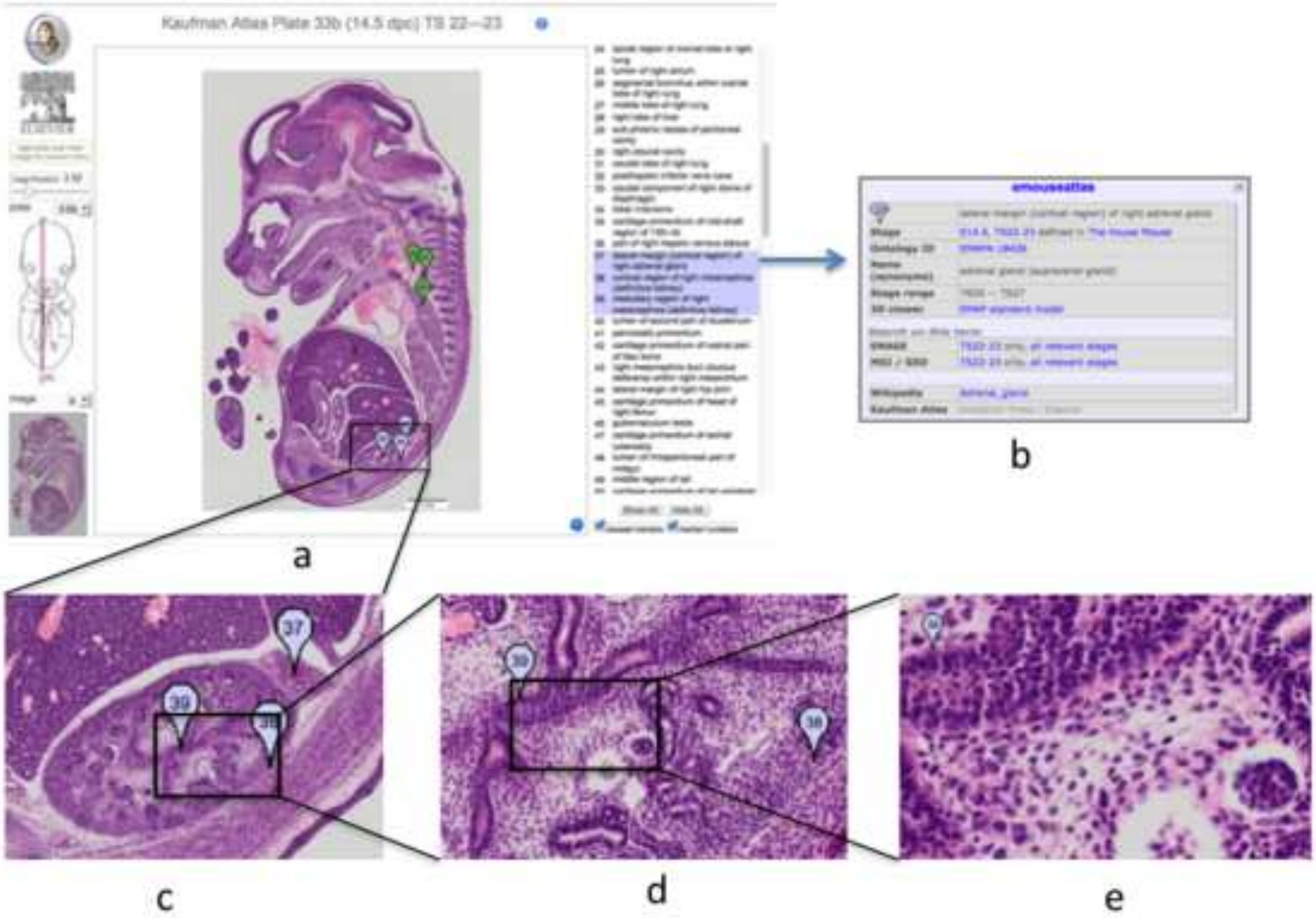

Figure 2

Click here to download Figure Fig_02.tif

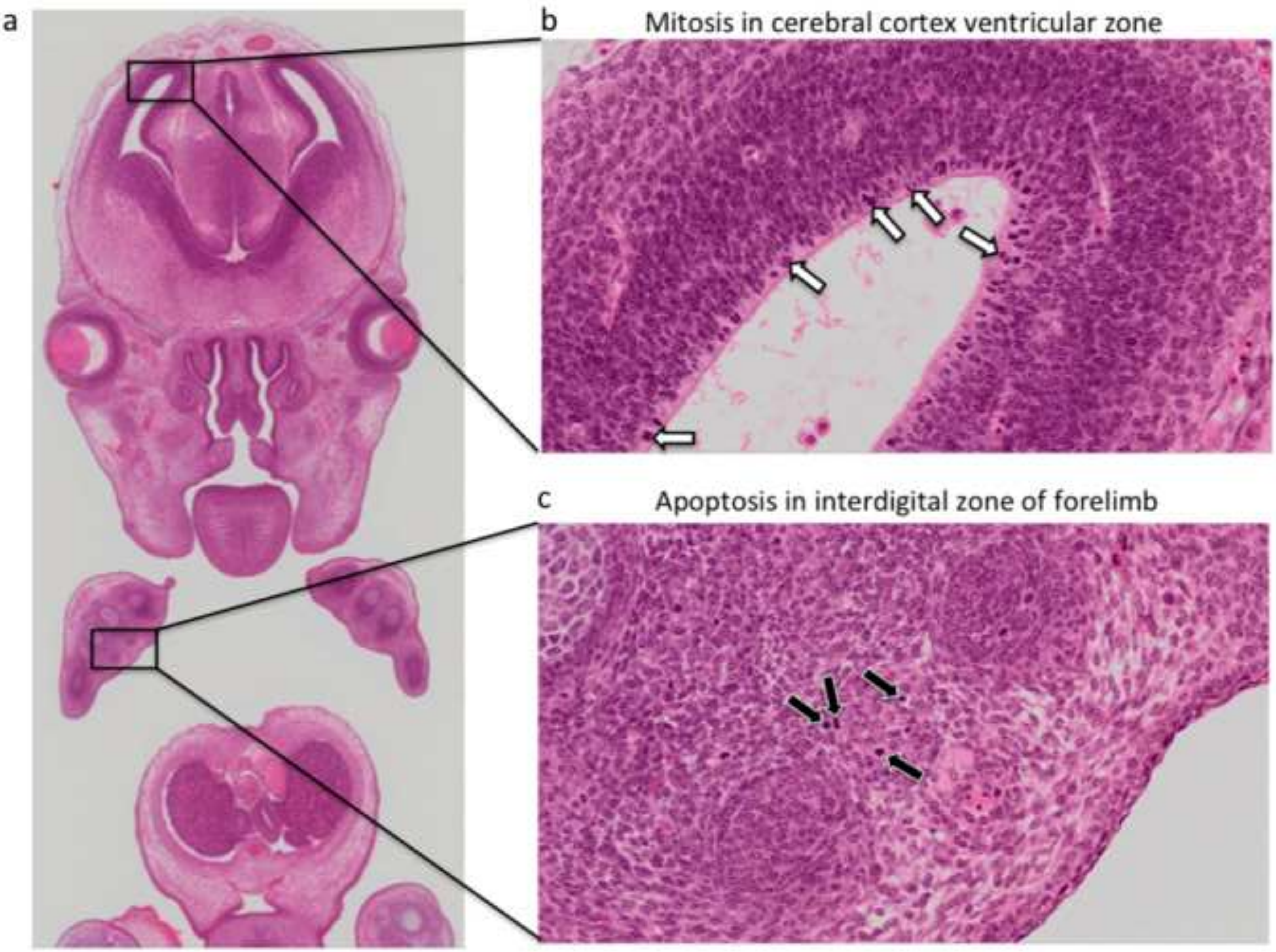

Click here to access/download
Supplementary Material
SciDataKaufmanTable3.xlsx
