# Supplementary material for: eHistology image and annotation data from the Kaufman Atlas of Mouse Development
Source: Gigascience. 2017 Dec 20;7(2):gix131. doi: 10.1093/gigascience/gix131 (PMC5827353; doi:10.1093/gigascience/gix131)
Supplement: Response_to_Reviewer_Comments_Original_Submission.pdf [file gix131_response_to_reviewer_comments_original_submission.pdf]

## Author's Response To Reviewer Comments

Dear GigaScience,

Thank you for considering our manuscript ‘eHistology image and annotation data from the Kaufman Atlas of Mouse Development’ (GIGA-D_17_00086) as a Data Note. We have revised the manuscript to accommodate the comments from the editorial board and the reviewers. We address each of these comments below.

Editorial board comment 1:

I can support publication, as this is the first time the annotations, coordinates, and images themselves have been published in a semi-structured form (e.g., EMAPA:XXXXX) that others could reuse.

Response to editorial board:

We are grateful of your support for this publication.

Editorial board comment 2:

I'd at least question whether the University of Edinburgh provides the best, most reliable repository for these data.

Response to editorial board:

The editorial board make a very good point and we have removed the statement “We suggest that the longevity provided by the University of Edinburgh will exceed any other option not associated with a similar institution.”

We see considerable value in having the data additionally hosted in the GigaDB repository, and we have added the statement ‘In addition and for convenience these data are also hosted in the GigaDB repository.’ If GigaDB were willing to host these data then would be delighted to additionally archive this dataset with you.

Editorial board comment 3:

The description of the ma-tech github repo needs much more detail.

Response to editorial board:

We have extended the ‘Code availability’ section of the manuscript and now refer to the WlzIIPSrv tiled image server and the eAtlasViewer javascript application. In addition, the github repository has been updated to include these applications.

Editorial board comment 4:

A clearer competing interest statement and information on the commercially annotations would also be useful, as well as more rationale on how this open resource differs.

Response to editorial board:

We have extended the competing interest statement to emphasise the open access nature of this dataset. Furthermore, we have revised the main body of the text to include examples of how the high-resolution images that we captured differ from those available in the print-version of the atlas. Specifically, we illustrate how the high-resolution atlas images can be used to morphologically identify mitotic and apoptotic cells in compartments of the developing embryo. This is not possible with the print-version of the atlas. To further emphasise this point, we now include an additional figure that details the rich information that is to be gained from capturing images at cellular-resolution.

Reviewer 1 comment 1:
The eHistology Viewer is mentioned in the abstract and shown in the figure. It would be helpful to include a URL link to that tool. (Right now there is only the link to the GitHub repository.)

Response to reviewer:

We now include a section on the ‘eHistology viewer’. This includes URLs, and additionally describes linking between Edinburgh Datashare and eHistology.

Reviewer 1 comment 2:

In part b of the figure (showing the pop-up box), taking that screenshot over white background -- instead of showing the semi-transparent view of the image in the background -- would clarify what is in that box.

Response to reviewer:

The reviewer makes a very good point and we have revised the figure to accommodate this change.

Reviewer 1 comment 3:

In Background & Summary, the wording "Providing for a secure (in terms of data preservation) and long-term accessibility…" is a bit awkward.

Response to reviewer:

We agree with the reviewer and we have revised this sentence to read:
Providing secure and long-term accessibility for research data is a difficult problem.

Reviewer 1 comment 4:

In Usage Notes, there appears to be some miswording, or perhaps a missing word, in "…which can be used to identify the set of physical glass slides help by the University of Edinburgh on which each histological section can be found."

Response to reviewer:

This was a type error and has been corrected. Furthermore, we have revised this section to expand on the physical location of the original glass slides. The amended sentence now reads as follows:
The 'source' can be used to identify the set of physical glass slides, archived with the Centre for Research Collections of the University of Edinburgh, on which each histological section can be found.

Reviewer 2 comment 1:

The annotation technology is not new and has been used and published for other histological images. Therefore, I am not sure what the point of this manuscript is other than that this technology has been applied to a popular, previously published resource. However, that has already been reported by Graham et al in a descriptive 2015 paper in the journal Development.

Response to reviewer:

The reviewer makes a very good point. However, there are significant differences between the original print-version of Kaufman's 'The Atlas of Mouse Development' and the image set that we have delivered. In the original version Kaufman chose a series of sections to be photographed, mounted on board and hand annotated using Letraset, then re-photographed to produce the grayscale plates used in the book. We obtained the original glass slides that were used in the Kaufman's 'The Atlas of Mouse Development' and we digitized these slides at high-resolution as there is added value in being able to visualise these sections in colour and at cellular-resolution. To emphasise the importance of the high-resolution version of the atlas, we now include an additional figure to illustrate how the cellular-resolution images that we have generated can be used to morphologically identify mitotic and apoptotic cells in compartments of the developing embryo. This is not possible with the print-version of the atlas.

The WlzIIPSrv tiled image server and the eAtlasViewer javascript application that we developed enabled point annotations to be added to the high-resolution images that we captured. The Graham et al (2015) Spotlight article in Development announced the eHistology resource and provided an overview of the eHistology viewer. However it was beyond the scope of this article to describe secure and long-term accessibility for image data that has been annotated in this way. In the current manuscript, we describe long-term accessibility to high-resolution images and supporting annotation of a highly accessed de facto atlas of mouse development, and we have described the approach we have adopted for data provenance. Furthermore, we now make available additional information that was not discussed in the Spotlight article. These include

details of source, position, and pixel resolution that would be needed for automated analysis of this image dataset. We have revised the ‘Usage notes’ to accommodate the reviewer’s comments, and we additionally include these data in a revised supplementary file. We hope this satisfies the reviewer.

Reviewer 2 comment 2:

One of the stated reasons for publishing the manuscript is the author's concern that data published online by public institutions likes universities might become lost if funding for maintaining the resource is no longer available. However, the high-resolution scans of Kaufman's images and the annotations have also been published commercially and, therefore, this concern does not apply to this work.
As the described data and work is available for sale by Elsevier, I need to question the author's statement that "they have no competing interests in the publication" of this manuscript.

Response to reviewer:

The reviewer makes a very good point and we have removed the statement “We suggest that the longevity provided by the University of Edinburgh will exceed any other option not associated with a similar institution.” However, the statement by the reviewer that “the high-resolution scans of Kaufman's images and the annotations have also been published commercially” is incorrect. The high-resolution digitized images that we have generated and the associated image-coordinates for each annotation are fully freely available under a Creative Commons CC BY 4.0 licence and nowhere else. We have an agreement with Elsevier to present the images in a form similar to the original atlas plate layout on the eHistology web resource. We have revised the main body of the text to emphasise the open access nature of the digitized high-resolution image dataset, and we have extended our ‘Competing interests’ statement to accommodate the reviewer’s concerns.
