# Supplementary material for: eHistology image and annotation data from the Kaufman Atlas of Mouse Development
Source: Gigascience. 2017 Dec 20;7(2):gix131. doi: 10.1093/gigascience/gix131 (PMC5827353; doi:10.1093/gigascience/gix131)
Supplement: Reviewer_1_Report_(Original_Submission).pdf -- Melissa Clarkson — 03 May 2017 Reviewed [file gix131_reviewer_1_report_(original_submission).pdf]

Reviewer Report

Title: eHistology image and annotation data from the Kaufman Atlas of Mouse Development

Version: Original Submission **Date:** 03 May 2017

Reviewer name: Melissa Clarkson

Reviewer Comments to Author:

Summary of paper: This paper describes work to bring Kaufman's classic atlas of mouse anatomy into a digital form. The methods used (including annotation with EMAPA IDs, use of DOIs, and Dublin Core elements) will preserve this information for future uses.

I have only a few minor suggestions for improving the manuscript:

1. The eHistology Viewer is mentioned in the abstract and shown in the figure. It would be helpful to include a URL link to that tool. (Right now there is only the link to the GitHub repository.)

2. In part b of the figure (showing the pop-up box), taking that screenshot over white background -- instead of showing the semi-transparent view of the image in the background -- would clarify what is in that box.

3. In Background & Summary, the wording "Providing for a secure (in terms of data preservation) and long-term accessibility..." is a bit awkward.

4. In Usage Notes, there appears to be some miswording, or perhaps a missing word, in "...which can be used to identify the set of physical glass slides help by the University of Edinburgh on which each histological section can be found."

Level of Interest

Please indicate how interesting you found the manuscript: An article of importance in its field

Quality of Written English

Please indicate the quality of language in the manuscript: Acceptable

Declaration of Competing Interests

Please complete a declaration of competing interests, considering the following questions:

- Have you in the past five years received reimbursements, fees, funding, or salary from an organisation that may in any way gain or lose financially from the publication of this manuscript, either now or in the future?
- Do you hold any stocks or shares in an organisation that may in any way gain or lose financially from the publication of this manuscript, either now or in the future?
- Do you hold or are you currently applying for any patents relating to the content of the manuscript?
- Have you received reimbursements, fees, funding, or salary from an organization that holds or has applied for patents relating to the content of the manuscript?
- Do you have any other financial competing interests?
- Do you have any non-financial competing interests in relation to this paper?

If you can answer no to all of the above, write 'I declare that I have no competing interests' below. If your reply is yes to any, please give details below.

I declare that I have no competing interests.

I agree to the open peer review policy of the journal. I understand that my name will be included on my report to the authors and, if the manuscript is accepted for publication, my named report including any attachments I upload will be posted on the website along with the authors' responses. I agree for my report to be made available under an Open Access Creative Commons CC-BY license (http://creativecommons.org/licenses/by/4.0/). I understand that any comments which I do not wish to be included in my named report can be included as confidential comments to the editors, which will not be published.

I agree to the open peer review policy of the journal
