# Supplementary material for: eHistology image and annotation data from the Kaufman Atlas of Mouse Development
Source: Gigascience. 2017 Dec 20;7(2):gix131. doi: 10.1093/gigascience/gix131 (PMC5827353; doi:10.1093/gigascience/gix131)
Supplement: Reviewer_2_Report_(Original_Submission).pdf -- Michael Hortsch — 09 May 2017 Reviewed [file gix131_reviewer_2_report_(original_submission).pdf]

Reviewer Report

Title: eHistology image and annotation data from the Kaufman Atlas of Mouse Development

Version: Original Submission **Date:** 09 May 2017

Reviewer name: Michael Hortsch

Reviewer Comments to Author:

In their manuscript "eHistology image and annotation data from the Kaufman Atlas of Mouse Development" the authors describe their work of digitalizing the images of the original book published by Matthew Kaufmann (first edition as a hardcover in 1992 published by Academic Press).
The annotation technology is not new and has been used and published for other histological images. Therefore, I am not sure what the point of this manuscript is other than that this technology has been applied to a popular, previously published resource. However, that has already been reported by Graham et al in a descriptive 2015 paper in the journal Development.
One of the stated reasons for publishing the manuscript is the author's concern that data published online by public institutions likes universities might become lost if funding for maintaining the resource is no longer available. However, the high-resolution scans of Kaufman's images and the annotations have also been published commercially and, therefore, this concern does not apply to this work.
As the described data and work is available for sale by Elsevier, I need to question the author's statement that "they have no competing interests in the publication" of this manuscript.

Level of Interest

Please indicate how interesting you found the manuscript: An article of limited interest

Quality of Written English

Please indicate the quality of language in the manuscript: Acceptable

Declaration of Competing Interests

Please complete a declaration of competing interests, considering the following questions:

- Have you in the past five years received reimbursements, fees, funding, or salary from an organisation that may in any way gain or lose financially from the publication of this manuscript, either now or in the future?

- Do you hold any stocks or shares in an organisation that may in any way gain or lose financially from the publication of this manuscript, either now or in the future?
- Do you hold or are you currently applying for any patents relating to the content of the manuscript?
- Have you received reimbursements, fees, funding, or salary from an organization that holds or has applied for patents relating to the content of the manuscript?
- Do you have any other financial competing interests?
- Do you have any non-financial competing interests in relation to this paper?

If you can answer no to all of the above, write 'I declare that I have no competing interests' below. If your reply is yes to any, please give details below.

None of the above apply.

I agree to the open peer review policy of the journal. I understand that my name will be included on my report to the authors and, if the manuscript is accepted for publication, my named report including any attachments I upload will be posted on the website along with the authors' responses. I agree for my report to be made available under an Open Access Creative Commons CC-BY license (http://creativecommons.org/licenses/by/4.0/). I understand that any comments which I do not wish to be included in my named report can be included as confidential comments to the editors, which will not be published.

I agree to the open peer review policy of the journal
